# ML212: A small-molecule probe for investigating fluconazole resistance mechanisms in *Candida albicans*

**DOI:** 10.3762/bjoc.9.171

**Published:** 2013-07-26

**Authors:** Willmen Youngsaye, Cathy L Hartland, Barbara J Morgan, Amal Ting, Partha P Nag, Benjamin Vincent, Carrie A Mosher, Joshua A Bittker, Sivaraman Dandapani, Michelle Palmer, Luke Whitesell, Susan Lindquist, Stuart L Schreiber, Benito Munoz

**Affiliations:** 1 Chemical Biology Platform and Probe Development Center, Broad Institute of MIT and Harvard, 7 Cambridge Center, Cambridge, MA 02142, USA; 2 Whitehead Institute for Biomedical Research, 9 Cambridge Center, Cambridge, MA 02142, USA; 3 Microbiology Graduate Program, Massachusetts Institute of Technology, 77 Massachusetts Avenue, Cambridge, MA 02139, USA; 4 Department of Biology and Howard Hughes Medical Institute, Massachusetts Institute of Technology, 77 Massachusetts Avenue, Cambridge, MA 02139, USA; 5 Howard Hughes Medical Institute, Broad Institute of Harvard and MIT, 7 Cambridge Center, Cambridge, MA 02142, USA

**Keywords:** antifungal, *Candida albicans*, chemosensitizer, fluconazole, Molecular Libraries Probe Production Center Network (MLPCN)

## Abstract

The National Institutes of Health Molecular Libraries and Probe Production Centers Network (NIH-MLPCN) screened >300,000 compounds to evaluate their ability to restore fluconazole susceptibility in resistant *Candida albicans* isolates. Additional counter screens were incorporated to remove substances inherently toxic to either mammalian or fungal cells. A substituted indazole possessing the desired bioactivity profile was selected for further development, and initial investigation of structure–activity relationships led to the discovery of ML212.

## Introduction

Discovery of antimicrobial agents possessing unique structural motifs or a novel mechanism of action is critical to counter and control the rising incidence of drug-resistant pathogens [1–6]. Chemosensitization of resistant organisms is a complementary approach that capitalizes upon the existing arsenal of antimicrobials to combat this medical dilemma [7–10]. By undermining the resistance mechanisms of the target pathogen, it is possible to restore efficacy to previously ineffective drugs thereby prolonging their status as frontline treatments. This, in turn, affords critical lead-time towards the development of novel antimicrobial drugs.

The National Institutes of Health Molecular Libraries and Probe Production Centers Network (NIH-MLPCN) recently performed a high-throughput screening (HTS) campaign to search for potential chemosensitizers of the pathogenic fungus *Candida albicans* [11]. The *C. albicans* clinical isolates used in this study demonstrate a range of resistance to the widely prescribed triazole antimycotic fluconazole (Flc) [12], and the objective was to identify novel small molecules capable of surmounting this inherent resistance [13–16]. The screen was conducted by using a cell-based assay with integrated counter screens to remove compounds acting through previously established methods for overturning drug resistance in *C. albicans*. In addition, substances intrinsically toxic to either mammalian or fungal cells were eliminated.

## Results and Discussion

### Screening results

**Figure 1:**
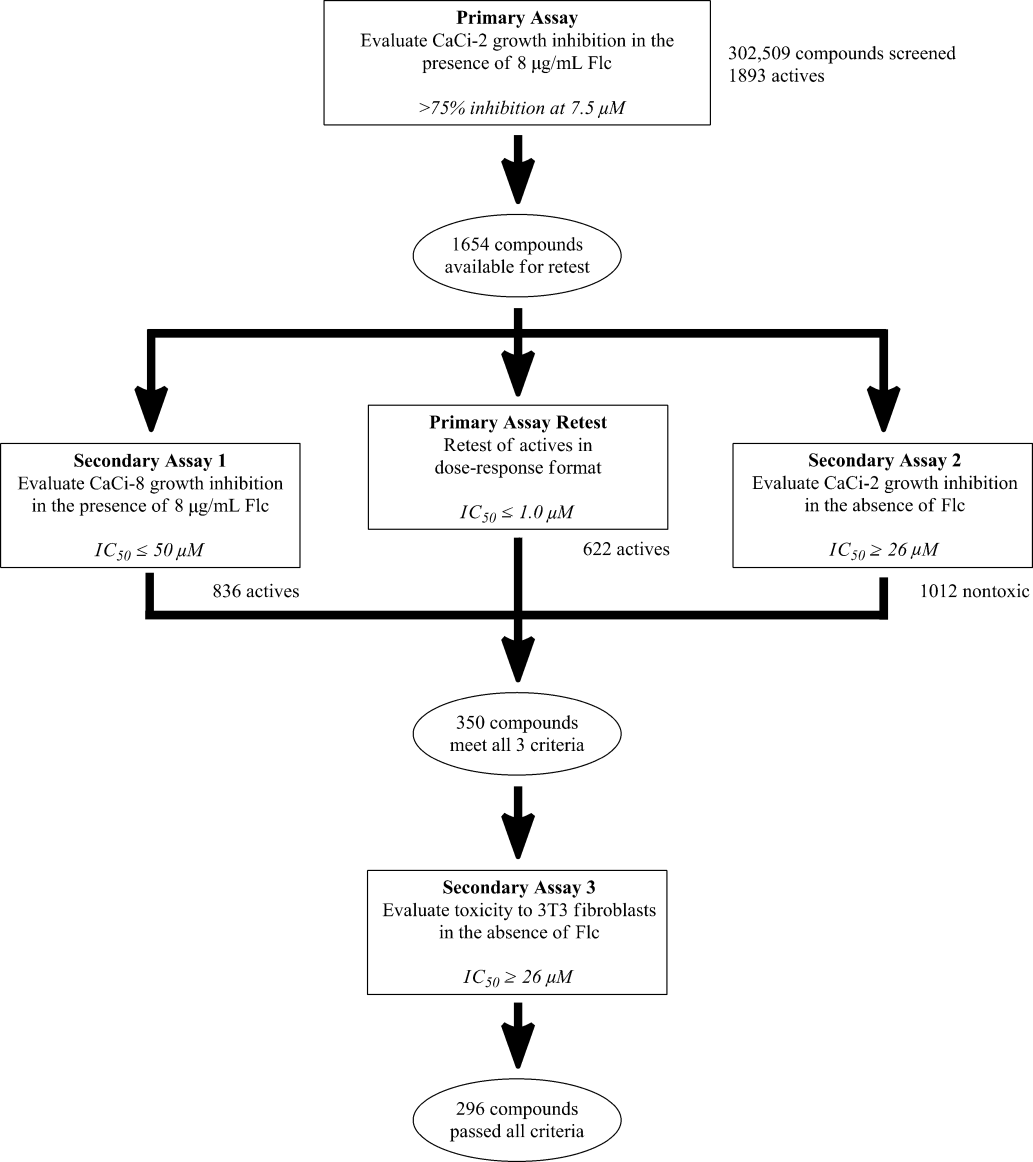
Assay pipeline for triaging hits. Individual assay cut-offs are given in italics.

In order to identify nonfungitoxic chemosensitizers of *Candida albicans*, compounds from the NIH’s Molecular Libraries Small Molecule Repository (MLSMR) [17] were evaluated in the screening cascade summarized in Figure 1. The *C. albicans* strains used in the primary screen and secondary assay 1 (CaCi-2 and CaCi-8, respectively) are both clinical isolates that partially respond to fluconazole treatment [12]. The minimum inhibitory concentration (MIC) of fluconazole against CaCi-2 and CaCi-8 was reported to be 2 µg/mL and 8 µg/mL, respectively [12]. However in our hands, these strains continue to proliferate steadily (albeit at a reduced rate) when treated with fluconazole at or above the reported MIC. This behavior may contribute to the inability of fluconazole therapy to effectively clear the infection and allows for the further development of resistance [12].

A total of 302,509 compounds from the MLSMR were tested at 7.5 µM for their ability to inhibit completely the growth of CaCi-2 cells that are concurrently treated with 8 µg/mL fluconazole (Figure 1). With a minimum requirement of 75% inhibition, 1893 actives were recorded, corresponding to an overall hit rate of 0.6%. Of the active compounds, 1654 were available for retesting in a dose-response format. This subset was resubjected to the primary assay and was also tested concurrently in two secondary assays. Secondary assay 1 measured chemosensitization of the more resistant CaCi-8 strain. Secondary assay 2 eliminated anything with inherently antifungal activity. These three assays cooperatively removed almost 80% of the original hits, leaving 350 candidates. A final assay (secondary assay 3) was incorporated to discard any compound displaying toxicity to mammalian fibroblasts. The fibroblast toxicity assay removed another 54 compounds to leave a total of 296 hits.

**Figure 2:**
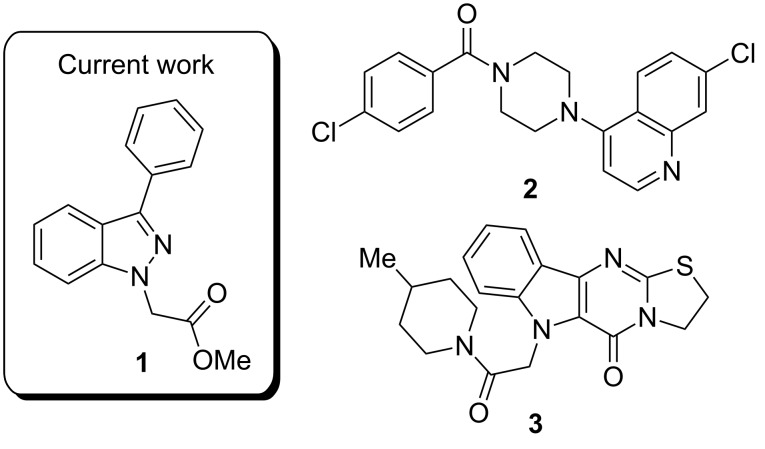
Hit compounds selected for further optimization.

From the 296 hits remaining, 29 were selected for revalidation from dry powders obtained from commercial vendors or resynthesis. Once their identity and purity was ascertained by LCMS and ^1^H NMR analysis, these 29 candidates were tested once more in the entire assay tree outlined previously in Figure 1. Following this re-evaluation, methyl 3-phenyl-1*H*-indazole acetate (**1**, Figure 2) emerged as an attractive candidate for further development. A commercial sample of compound **1** shows good activity against CaCi-2 and CaCi-8 (IC_50_ = 0.8 and 2.3 μM, respectively) with no apparent effect on 3T3 fibroblasts (IC_50_ > 26 μM). Compound **1** also possesses good solubility in PBS (79 μM) and is synthetically tractable with several points of diversity readily accessible. Consequently, compound **1** and a collection of closely related analogues were prepared to enable investigation of possible structure–activity relationships (SAR) [18]. In addition to indazole **1**, two other structurally distinct scaffolds (**2** and **3**) were also selected for follow-up studies and those works are communicated elsewhere [19–20].

### Chemistry

**Scheme 1:**
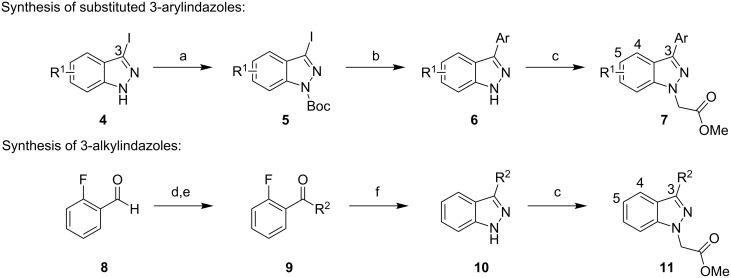
Preparation of substituted methyl 2-(1*H*-indazol-1-yl)acetates. Reagents and conditions: (a) Et_3_N, Boc_2_O, DMAP, tetrahydrofuran; (b) ArB(OH)_2_, Pd(PPh_3_)_4_, aqueous Na_2_CO_3_, 1,4-dioxane, 120 °C; (c) methyl bromoacetate, K_2_CO_3_, acetone, 60 °C; (d) alkylmagnesium bromide, Et_2_O, 0 °C; (e) Dess–Martin periodinane, CH_2_Cl_2_; (f) hydrazine hydrate, 175 °C.

Two different routes were adopted to access the various functionalized indazoles required to evaluate the SAR associated with hit compound **1** (Scheme 1). The robust Suzuki–Miyaura reaction was selected for the preparation of analogues bearing substituents around the central indazole core. This approach also permitted rapid replacement of the phenyl ring at C3 with functionalized phenyl rings and alternative heterocycles. Preliminary attempts to couple substituted 3-iodoindazoles **4** failed to produce isolable amounts of the desired product directly. Subsequently, the indazoles were protected as their *tert*-butyl carbamates **5** prior to undergoing palladium-mediated Suzuki reactions with various boronic acid partners. Under the reaction conditions, the carbamate-protecting group was also cleaved to afford the desired 3-arylindazoles, albeit as the unprotected systems **6**. Alkylation with methyl bromoacetate and potassium carbonate in hot acetone completed the synthesis of compounds **7**.

In order to prepare 3-alkylindazoles, 2-fluorobenzaldehyde was first treated with alkylmagnesium bromides, and the resulting benzyl alcohols were immediately oxidized with Dess–Martin reagent. Alkyl phenyl ketones **9** and hydrazine hydrate were then reacted under microwave conditions to assemble the indazole ring **10**. The ester side chain of **11** was installed under the same conditions described above for the alkylation of 3-arylindazoles.

### In vitro activity and SAR

All of the compounds prepared above were evaluated for their ability to chemosensitize the *C. albicans* test strains CaCi-2 and CaCi-8 towards fluconazole. As described above, the fungi were simultaneously incubated with test compounds and fluconazole (8 µg/mL) for 48 hours to determine if any combinations could fully inhibit fungal growth. The DMSO/fluconazole combination served as an internal control.

Substitution of the indazole core generally leads to a potency reduction to varying degrees (Table 1). The 5- and 6-positions can accommodate smaller functionalities such as methyl or chloro groups (**11**, **15**, and **17**) and still retain modest efficacy. The inactivity of **14** compared to **17** suggests that the 6-position may occupy a slightly larger pocket. Electron-withdrawing substituents (**13**, **18**, and **19**) appear detrimental to activity regardless of their position, while the weak potency of the methoxy compounds (**12** and **16**) suggests that there may be a limit to how far the western region may be extended. Pyridylindazole **20**, wherein a nitrogen atom is inserted in the place of a carbon, displays no cellular activity. Based on these preliminary results, a more extensive SAR investigation of this region was postponed in order to explore other regions of the scaffold.

**Table 1:** Activity of substituted indazole cores.^a^

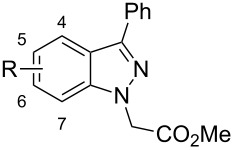			

Cpd	R	CaCi-2 IC_50_ (µM)^b^	CaCi-8 IC_50_ (µM)^b^

**1**	H	2.2 ± 1.0	3.5 ± 1.9
**11**	5-Me	5.2 ± 2.2	8.2 ± 3.2
**12**	5-OMe	14.9 ± 5.7	22.1 ± 6.2
**13**	5-F	11.2 ± 7.2	inactive
**14**	5-Cl	inactive	inactive
**15**	6-Me	5.9 ± 3.1	inactive
**16**	6-OMe	9.5 ± 2.5	12.4 ± 0.5
**17**	6-Cl	4.0 ± 1.3	8.4 ± 0.5
**18**	6-CF_3_	inactive	inactive
**19**	7-CF_3_	inactive	inactive
**20**	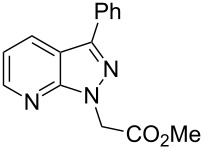	inactive	inactive

^a^CaCi-2 and CaCi-8 cells were incubated at 37 °C for 48 hours with test compound and 8 µg/mL (26 µM) fluconazole. ^b^Average of at least three independent experiments, performed in duplicate. Inactive compounds displayed negligible activity at concentrations below 26 µM.

We proceeded to evaluate the SAR of the C3 substituent next (Table 2). Removal of the original benzene ring produces the inactive analogue **21**. Similarly, installing acyclic alkyl systems such as an ethyl (**22**) or *tert*-butyl group (**23**) does not yield active compounds. Proceeding to cycloalkanes, a SAR trend begins to emerge. With the smallest cycloalkane replacement, the cyclopropyl derivative **24** is inactive whereas the larger cyclohexane of **25** yields an active chemosensitizer (IC_50_ = 2.3 μM). When alternative heteroaromatic rings (**26**–**29**) were prepared, none of the examined systems possess any significant cellular activity.

**Table 2:** Investigation of 3-substituted indazoles.^a^

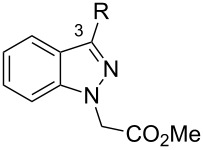			

Cpd	R	CaCi-2 IC_50_ (µM)^b^	CaCi-8 IC_50_ (µM)^b^

**21**	-H	inactive	inactive
**22**	-Et	inactive	inactive
**23**	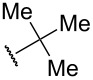	inactive	inactive
**24**	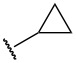	inactive	inactive
**25**	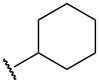	2.3 ± 0.3	6.0 ± 1.7
**26**	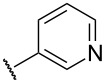	inactive	inactive
**27**	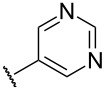	inactive	21.1 ± 3.3
**28**	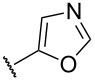	inactive	inactive
**29**	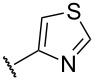	12.9 ± 1.7	inactive

^a^CaCi-2 and CaCi-8 cells were incubated at 37 °C for 48 hours with test compound and 8 µg/mL (26 µM) fluconazole. ^b^Average of at least three independent experiments, performed in duplicate. Inactive compounds displayed negligible activity at concentrations below 26 µM.

One possible explanation for the observed activity is that the binding pocket includes a small cleft that is best occupied by flat structures. Compounds **21**, **22**, and **24** are presumably too small to fit properly into this crevice, while the *tert*-butyl derivative **23** may simply be too large. However, the inactivity of the heteroaromatic counterparts **26**–**29** may imply that more than a steric constraint is operative within this putative groove; a possible electronic requirement for this substituent may exist as well.

Returning to the original phenyl ring, various substituents were introduced at different positions about the ring to probe for further electronic and steric effects associated with this region (Table 3). With regards to steric requirements, it appears that neither the *para*- (compounds **30**–**34**) nor *ortho*-positions (compounds **39** and **40**) are particularly amenable to functionalization. While bioactivity is still observed with these substances, they all appear to be only weakly active (IC_50_ = 2–8 μM). Conversely, the *meta*-substituted systems **35**–**38** prove to be the most conducive to potency. The methyl ether **36** is potent against CaCi-2 (IC_50_ = 0.7 μM) as is the *N*,*N*-dimethylamine variant (**37**, IC_50_ = 0.8 μM). The weakest compound of this series, 3-fluoro derivative **38**, still shows low micromolar activity (IC_50_ = 1.7 μM). The weak activity of **38** may be tied to the electronegativity of fluorine. This is best illustrated with compounds **32**–**34**. While the 4-fluoro analogue is a weak chemosensitizer of CaCi-2 and CaCi-8, introducing more electron-withdrawing substituents, such as trifluoromethyl (**33**) and cyano (**34**) groups, results in complete inactivity (IC_50_ > 26 µM). The activity of *p*-tolyl **30** and *p*-anisoyl **31** indicates that the inefficacy of **33** and **34** cannot be solely attributed to steric considerations. The inactivity of the 3,5-dimethoxyphenyl ring (**41**) suggests the indazole’s C3 substituent may reside in an asymmetric pocket.

**Table 3:** Substituent effects associated with the 3-phenyl ring.^a^

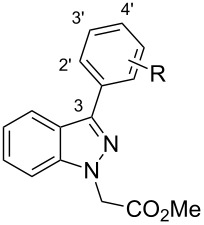			

Cpd	R	CaCi-2 IC_50_ (µM)^b^	CaCi-8 IC_50_ (µM)^b^

**1**	H	2.2 ± 1.0	3.5 ± 1.9
**30**	4′-Me	4.8 ± 2.5	19.5 ± 14.2
**31**	4′-OMe	4.2 ± 1.7	14.1 ± 6.4
**32**	4′-F	8.3 ± 2.8	12.3 ± 3.9
**33**	4′-CF_3_	inactive	inactive
**34**	4′-CN	inactive	20.5 ± 0.3
**35**	3′-Me	1.1 ± 0.6	1.9 ± 0.9
**36**	3′-OMe	0.7 ± 0.3	1.5 ± 0.6
**37**	3′-NMe_2_	0.8 ± 0.1	2.0 ± 0.2
**38**	3′-F	1.7 ± 0.4	4.2 ± 4.1
**39**	2′-Me	5.3 ± 0.5	7.8 ± 2.6
**40**	2′-OMe	9.3 ± 3.6	11.4 ± 1.7
**41**	3′,5′-di-OMe	inactive	inactive

^a^CaCi-2 and CaCi-8 cells were incubated at 37 °C for 48 hours with test compound and 8 µg/mL (26 µM) fluconazole. ^b^Average of at least three independent experiments, performed in duplicate. Inactive compounds displayed negligible activity at concentrations below 26 µM.

As the most potent chemosensitizer of both *C. albicans* CaCi-2 and CaCi-8 (IC_50_ = 0.7 and 1.5 µM, respectively) with good solubility (55 µM in PBS), compound **36** was nominated as MLPCN probe ML212. Exposure to human or murine plasma revealed significant chemical instability (<10% remaining after 5 h incubation), and this is attributed to the ester hydrolysis of the side chain. Follow-up studies investigating the SAR of the side chain and addressing this liability will be reported shortly. Additional profiling of ML212 determined that the probe is nontoxic to *C. albicans* in the absence of fluconazole (IC_50_ > 26 µM after 48 h incubation), neither does it show any toxicity towards murine 3T3 fibroblasts (IC_50_ > 26 µM). Hsp90-dependent and calcineurin-dependent signaling pathways have been previously implicated in maintaining fluconazole resistance in *C. albicans* [21], but ML212 does not inhibit these pathways in yeast-reporter assays (IC_50_ > 26 µM). Identification of ML212’s molecular target is ongoing, as well as efforts to determine the efficacy of ML212 against diverse mechanisms of fluconazole resistance, including biofilm formation, drug-target mutations, and efflux-pump amplification.

## Conclusion

High-throughput screening of 300,000 compounds from the NIH’s MLSMR collection identified several substances that potentiate the effect of fluconazole in fluconazole-resistant *Candida albicans* clinical isolates. Among the numerous hits, 3-phenylindazole **1** was selected for chemical optimization, resulting in the identification of 3-(3-anisoyl)indazole **36** as new small-molecule probe (ML212) to facilitate investigation of the various mechanisms used by *C. albicans* to withstand fluconazole. Elucidation of ML212’s mechanism of action may afford new targets to exploit in the continuing efforts to develop novel antimycotics and combat increasingly prevalent drug-resistance. Samples of ML212 are available free of charge, on request.

## Supporting Information

Detailed experimental protocols for cellular assays and for the preparation of representative compounds **25** and **36** are provided. Proton NMR spectra for all prepared compounds are also available.

File 1 Detailed assay protocols and compound synthesis.

File 2 NMR spectra of reported compounds.
