# Supplementary material for: ML212: A small-molecule probe for investigating fluconazole resistance mechanisms in Candida albicans
Source: Beilstein J Org Chem. 2013 Jul 26;9:1501–7. doi: 10.3762/bjoc.9.171 (PMC3740683; doi:10.3762/bjoc.9.171)
Supplement: File 1 — Detailed assay protocols and compound synthesis. [file Beilstein_J_Org_Chem-09-1501-s001.pdf]

Supporting Information

for

ML212: A small-molecule probe for investigating fluconazole resistance mechanisms in *Candida albicans*

Willmen Youngsaye[1], Cathy L. Hartland[1], Barbara J. Morgan[1], Amal Ting[1], Partha P. Nag[1], Benjamin Vincent[2,3], Carrie A. Mosher[1], Joshua A. Bittker[1], Sivaraman Dandapani[1], Michelle Palmer[1], Luke Whitesell[2], Susan Lindquist[2,4], Stuart L. Schreiber[1,5] and Benito Munoz[1,*]

Address: [1]Chemical Biology Platform and Probe Development Center, Broad Institute of MIT and Harvard, 7 Cambridge Center, Cambridge, MA 02142, USA, [2]Whitehead Institute for Biomedical Research, 9 Cambridge Center, Cambridge, MA 02142, USA, [3]Microbiology Graduate Program, Massachusetts Institute of Technology, 77 Massachusetts Avenue, Cambridge, MA 02139, USA, [4]Department of Biology and Howard Hughes Medical Institute, Massachusetts Institute of Technology, 77 Massachusetts Avenue, Cambridge, MA 02139, USA and [5]Howard Hughes Medical Institute, Broad Institute of Harvard and MIT, 7 Cambridge Center, Cambridge, MA 02142, USA

Email: Benito Munoz - bmunoz@broadinstitute.org

* Corresponding author

Detailed assay protocols and compound synthesis

Protocols for cellular assays S2

Compound synthesis S9

NMR and MS data for reported compounds S15

Protocols for cellular assays

Primary screen and dose-response retest: CaCi-2 with 8 μg/mL fluconazole

Materials and reagents:

Clear, flat-bottom, black, 384-well plates (Corning Catalog no. 3712BC; Lot no. 35808016); geldanamycin (AG Scientific, Catalog no. G-1047) 15 mM stock solution in DMSO; Fluconazole (Sequoia Research Products Ltd) 2 mg/mL stock solution in PBS; Pen/Strep (Gibco Catalog no.10378-016; Lot no. 21040170) 100× in PBS; Alamar Blue (AG Scientific Catalog no. DAL 1100; Lot no.151016SA); PBS without calcium and magnesium (Cellgro Catalog no. 21-040-CV)

Synthetic defined growth medium

RPMI 1640 medium, (powder without sodium bicarbonate; Invitrogen Catalog no. 31800-089; Lot no. 648072); uridine 8 mg/mL in water (Sigma Catalog no. U3750; Lot no. 028KO760); Glucose 40% (w/v) in water (Sigma Catalog no. G-5400); MOPS buffer (Sigma Catalog no. M-1254; Lot no. 098K0033)

1) Prepare 1× RPMI medium by dissolving 10.4 grams powdered medium in 800 mL water.
2) Add 34.52 g MOPS. While stirring, adjust pH to 7.0 with 10 N NaOH.
3) Add 10 mL uridine solution, 50 mL glucose solution, adjust final volume to 1000 mL. Filter sterilize.

Fungal Inoculum

Test Strain: *C. albicans* CaCi-2

1) Inoculate 500 µL of strain from cryopreserved stock into a 250 mL shaker flask containing 30 mL growth medium. Shake at 30 °C overnight.
2) Read OD 600 of 1 mL fungal culture in a cuvette using a standard optical density reader (Eppendorf BioPhotometer Plus), with growth medium as a background blank.

3) Dilute to desired volume of fungal inoculums according to the following formula: (1/OD measured) × (desired final volume of inoculum) × 0.3 = volume of fungal culture (μL) to add to desired volume of growth medium. When added to media in wells, this yields a calculated starting OD of the fungal inoculum of 0.00015.

Procedures:

1) Add fluconazole stock solution to fungal inoculum to achieve a final concentration of 8 μg/mL.
2) Add Pen/Strep at 0.1 mL per 10 mL media (1% v/v).
3) Use a Thermo Combi nL to dispense 20 μL/well of assay media into all wells.
4) Pin 25 nL test compound from compound plates into assay plates using CyBi-Well pin tool.
5) Dispense 20 μL/well of culture into the assay media in all wells.
6) Incubate plates in a humidified (90% humidity) Liconic incubator at 37 °C without agitation for 48 hours.
7) Dilute Alamar Blue Reagent 1:40 in Ca/Mg-free PBS.
8) To all plates, add 5 μL/well of the diluted Alamar to a final dilution factor of 1:200.
9) Incubate the plates for an additional 2 hours.
10) Read the relative fluorescence intensity (RFU) of wells on a standard plate reader as a measure of relative fungal growth. EnVision (Perkin Elmer) plate reader setup: Ex 544 nm, Em 590 nm, bandwidth 12 nm, top read.

Secondary assay 1: CaCi-8 with 8 μg/mL fluconazole

Materials and reagents:

Clear, flat-bottom, black, 384-well plates (Corning Catalog no. 3712BC; Lot no. 35808016); geldanamycin (AG Scientific Catalog no. G-1047) 15 mM stock solution in DMSO; Pen/Strep (Gibco Catalog no.10378-016;Lot no.21040170) 100× in PBS; fluconazole (Sigma Catalog no.F829-100MG; Lot no. 098K4715) 2 mg/mL stock solution in PBS; Alamar Blue (AG Scientific Catalog no. DAL1100; Lot no.151016SA); PBS w/o calcium and magnesium (Cellgro Catalog no. 21-040-CV)

Synthetic defined growth medium

RPMI 1640 medium, (powder without sodium bicarbonate; Invitrogen Catalog no. 31800-089, Lot no.648072); uridine 8 mg/mL in water (Sigma Catalog no. U3750; Lot no. 028K0760); glucose 40% (w/v) in water (Sigma Catalog no. G-5400); MOPS buffer (Sigma Catalog no. M-1254; Lot no. 098K0033)

1) Prepare 1× RPMI medium by dissolving 10.4 g powdered medium in 800 mL water.
2) Add 34.52 g MOPS. While stirring, adjust pH to 7.0 with 10 N NaOH.
3) Add 10 mL uridine solution, 50 mL glucose solution, adjust final volume to 1000 mL. Filter sterilize.

Fungal inoculum

Test strain: *C. albicans* CaCi-8

1) Inoculate 500 µL of strain from cryopreserved stock into a 250 mL shaker flask containing 30 mL growth medium. Shake at 30 °C overnight (16 hours).
2) Read OD 600 of 1 mL of fungal culture in a cuvette using a standard optical density reader (Eppendorf BioPhotometer Plus), with growth medium as a background blank.
3) Dilute to a desired volume of fungal inoculum according to following formula: (1/OD measured) × (desired final volume of inoculum) × 0.3 = volume of fungal culture (µL) to add to desired volume of growth medium. When added to media in wells, this yields a calculated starting OD of the fungal inoculum of 0.00015.

Procedures:

1) Add fluconazole stock solution to fungal inoculum to achieve 8 µg/mL.
2) Add Pen/Strep to media to 1% concentration.
3) Use a Thermo Combi nL to dispense 20 µL/well of assay media into all wells.
4) Dispense geldanamycin in positive control wells using Thermo Combi nL for a final concentration of 3 µM.

5) Then, pin 100 nL of test compound from compound plates into assay plates using a CyBi-Well pin tool.
6) Dispense 20 µL/well of culture into the assay media in all wells.
7) Incubate the plates in a humidified (90% humidity) Liconic incubator at 37 °C without agitation for 48 hours.
8) Dilute Alamar Blue 1:40 in Ca/Mg-free PBS.
9) To all plates, add 5 µL/well of the diluted Alamar Blue to plates to a final dilution factor of 1:200.
10) Incubate the plates for 2 hours.
11) Read the relative fluorescence intensity (RFU) of wells on a standard plate reader as a measure of relative fungal growth. EnVision (Perkin Elmer) plate reader setup: Ex 544 nm, Em 590 nm, bandwidth 12 nm, top read.

Secondary assay 2: CaCi-2 in the absence of fluconazole

Materials and reagents:

Clear, flat-bottom, black 384-well plates (Corning Catalog no. 3712BC; Lot no. 35808016); geldanamycin (AG Scientific Catalog no. G-1047) 15 mM stock solution in DMSO; Pen/Strep (Gibco Catalog no. 10378-016; Lot no21040170) 100× in PBS; Fluconazole (Sigma Catalog no. F829-100MG; Lot no. 098K4715) 2 mg/mL stock solution in PBS; Alamar Blue (AG Scientific Catalog no. DAL1100; Lot no.151016SA);PBS w/o calcium and magnesium (Cellgro Catalog no. 21-040-CV)

Synthetic defined growth medium

RPMI 1640 medium, (powder without sodium bicarbonate; Invitrogen 31800-089, Lot 648072); uridine 8 mg/mL in water (Sigma Catalog no. U3750; Lot no. 028K0760); Glucose 40% (w/v) in water (Sigma Catalog no. G-5400); MOPS Buffer (Sigma Catalog no. M-1254; Lot no. 098K0033)

1) Prepare 1X RPMI medium by dissolving 10.4 g powdered medium in 800 mL water.

2) Add 34.52 g MOPS. While stirring, adjust pH to 7.0 with 10 N NaOH.
3) Add 10 mL uridine solution, 50 mL glucose solution, adjust final volume to 1000 mL. Filter sterilize.

Fungal inoculum

Test strain: *C. albicans* CaCi-2

1) Inoculate 500 µL of yeast from cryopreserved stock into a 250 mL shaker flask containing 30 mL growth medium. Shake at 30 °C overnight (16 hours).
2) Read OD 600 of 1 mL of fungal culture in a cuvette using a standard optical density reader (Eppendorf BioPhotometer Plus), with growth medium as a background blank.
3) Dilute to desired volume of fungal inoculum according to following formula: (1/OD measured) × (desired final volume of inoculum) × 0.3 = volume of fungal culture (µL) to add to desired volume of growth medium. When added to media in wells, this yields a calculated starting OD of the fungal inoculum of 0.00015.

Procedures:

1) Add Pen/Strep to the media to a final 1% concentration.
2) Use a Thermo Combi nL to dispense 20 µL/well of assay media into all wells.
3) Mix geldanamycin and fluconazole for positive control.
4) Dispense positive control solution into the positive control wells using Thermo Combi nL for a final concentration of 3 µM geldanamycin, and 8 µg/mL fluconazole.
5) Then, pin 100 nL of test compound from compound plates into assay plates using a CyBi-Well pin tool.
6) Dispense 20 µL/well of culture into the assay media in all wells.
7) Incubate the plates in a humidified (90% humidity) Liconic incubator at 37 °C without agitation for 48 hours.
8) Dilute Alamar Blue 1:40 in Ca/Mg-free PBS.
9) To all plates, add 5 µL/well of the diluted Alamar Blue to the plates to a final dilution factor 1:200.
10) Incubate the plates for 2 hours.

11) Read relative fluorescence intensity (RFU) of wells on a standard plate reader as measure of relative fungal growth. EnVision (Perkin Elmer) plate reader settings: Ex 544 nm, Em 590 nm, bandwidth 12 nm, top read.

Secondary assay 3: fibroblast toxicity

Materials and reagents:

Clear, flat-bottom, black, 384-well plates (Corning Catalog no. 3712BC Lot no. 35808016); geldanamycin (AG Scientific Catalog no. G-1047) 15 mM stock solution in DMSO; fluconazole (Sequoia Research Ltd.) 2 mg/mL stock solution in PBS; Alamar Blue (AG Scientific Catalog no. DAL1100, Lot no. 151016SA); PBS w/o calcium and magnesium (Cellgro Catalog no. 21-040-CV)

Assay medium

Optimem medium (Invitrogen Catalog no. 31985-070; Lot no. 548536); 2.5% (v/v) fetal bovine serum (Hyclone Catalog no.30071.03; Lot no. ARF26748); 1% (v/v) Pen/Strep solution (Invitrogen Catalog no.15140-122; Lot no. 529891)

Cell inoculum

Test strain: NIH-3T3 mammalian fibroblasts (ATCC CRL No. 1658)

1) Plate cells in 384-well plates at 6,000 cells/well in 20 µL assay medium.
2) Incubate plates overnight at 37 °C under 5% CO_2.

Procedures:

1) After overnight culture, pin compounds into wells at 100 nL/well using the CyBio CyBi-Well pinning instrument.
2) After pinning compounds, add 20 µL of assay medium supplemented with fluconazole to each well. To a final nominal concentration of 8 µg/ml fluconazole.
3) Return the plates to the tissue culture incubator and incubate the culture for an additional 48 hours at 37 °C under 5% CO_2.

4) At the completion of this incubation, add Alamar Blue solution diluted 1:40 in PBS to each well (10 µL/well) to achieve a final dilution of 1:200.
5) Incubate the plates for an additional 2–3 hours at 37 °C under 5% CO_2.
6) Read the relative fluorescence intensity (RFU) of wells on a standard plate reader as a measure of relative cell growth. EnVision (Perkin Elmer) plate reader setup: Ex 544 nm, Em 590 m, bandwidth12 nm, top read.

Compound synthesis

General details. All reagents and solvents were purchased from commercial vendors and used as received. NMR spectra were recorded on a Bruker 300 MHz spectrometer. Proton and carbon chemical shifts are reported in ppm (δ) relative to tetramethylsilane (^{1}H δ 0.00) or residual chloroform in $CDCl_3$ solvent (^{1}H δ 7.24, ^{13}C δ 77.0). NMR data are reported as follows: chemical shifts, multiplicity (obs. = obscured, br = broad, s = singlet, d = doublet, t = triplet, q = quartet, m = multiplet); coupling constant(s) in Hz; integration.

Unless otherwise indicated, NMR data were collected at 25 °C. Flash chromatography was performed using 40–60 µm silica gel (60 Å mesh) on a Teledyne Isco Combiflash Rf system. Tandem liquid chromatography/mass spectrometry (LC/MS) was performed on a Waters 2795 separations module and 3100 mass detector. Analytical thin layer chromatography (TLC) was performed on EM Reagent 0.25 mm silica gel 60-F plates. Visualization was accomplished with UV light and aqueous potassium permanganate ($KMnO_4$) stain followed by heating. Microwave reactions were performed with a Biotage Initiator 2.5 Microwave Synthesizer. High-resolution mass spectra were obtained at the MIT Mass Spectrometry Facility with a Bruker Daltonics APEXIV 4.7 Tesla Fourier Transform Ion Cyclotron Resonance mass spectrometer. Compound purity and identity were determined by UPLC–MS (Waters, Milford, MA). Purity was measured by UV absorbance at 210 nm. Identity was determined on an SQ mass spectrometer by positive electrospray ionization. Mobile Phase A consisted of either 0.1% ammonium hydroxide or 0.1% trifluoroacetic acid in water, while mobile Phase B consisted of the same additives in acetonitrile. The gradient ran from 5% to 95% mobile Phase B over 0.8 minutes at 0.45 mL/min. An Acquity BEH C18, 1.7 µm, 1.0 × 50 mm column was used with column temperature maintained at 65 °C. Compounds were dissolved in DMSO at a nominal concentration of 1 mg/mL, and 0.25 µL of this solution was injected.

Representative preparation of substituted methyl 3-arylindazolyl acetates:

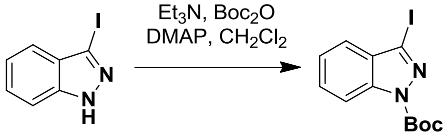

S1 S2

***tert*-Butyl 3-iodo-1*H*-indazole-1-carboxylate (S2):** 3-Iodo-1*H*-indazole (**S1**, 5.00 g, 19.5 mmol) was placed in a round-bottom flask and dissolved in tetrahydrofuran (100 mL). 4-Dimethylaminopyridine (0.24 g, 1.9 mmol, 0.1 equiv) was then added, followed by di-*tert*-butyl dicarbonate (5.4 mL, 24 mmol, 1.2 equiv). Triethylamine (5.4 mL, 39 mmol, 2.0 equiv) was slowly added to the clear, brown solution by syringe. The resulting solution was stirred at room temperature until it was complete as determined by TLC. The reaction was then diluted with water (75 mL) and ethyl acetate (50 mL). After separating the layers, the aqueous phase was extracted with additional ethyl acetate (3 × 50 mL). The combined organic layers were washed with brine (100 mL), then shaken over magnesium sulfate, filtered, and concentrated under reduced pressure to give the crude product. This material was purified by column chromatography over silica gel (hexanes/ethyl acetate: 100/0 to 90/10) to give the title compound as an orange solid (6.20 g, 93%). ^{1}H NMR (300 MHz, $CDCl_3$): δ 8.12 (d, *J* = 8.4 Hz, 1H), 7.59 (t, *J* = 7.7 Hz, 1H), 7.50 (d, *J* = 7.9 Hz, 1H), 7.37 (t, *J* = 7.5 Hz, 1H), 1.73 (s, 9H); ^{13}C NMR (75 MHz, $CDCl_3$): δ 148.3, 139.6, 130.2, 129.9, 124.1, 121.9, 114.5, 102.8, 85.4, 28.1; ESI–MS ($M-C_4H_9$): m/z 288.

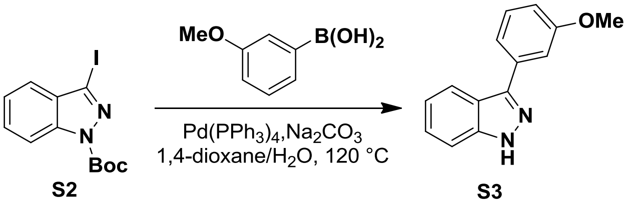

S2 S3

3-(3-methoxyphenyl)-1*H*-indazole (S3): *tert*-butyl 3-iodo-1*H*-indazole-1-carboxylate (**S2**, 100 mg, 0.29 mmol) was placed in a microwave vial and dissolved in 1,4-dioxane (11.5 mL). 3-Methoxyphenyl boronic acid (88 mg, 0.58 mmol, 2.0 equiv) and tetrakis(triphenylphosphine) palladium (20 mg, 0.017 mmol, 0.06 equiv) were added, and the resulting mixture was sparged thoroughly with nitrogen**.** An aqueous solution of

sodium carbonate (2.0 M, 0.65 mL, 1.3 mmol, 4.5 equiv) was then added. The biphasic mixture was microwaved for 1 hour at a reaction temperature of 120 °C. After cooling to room temperature, the reaction was diluted with ethyl acetate (2 mL), and then filtered through a celite pad with additional ethyl acetate. The filtrate was concentrated under reduced pressure to give an oil. The crude material was purified by column chromatography over silica gel (hexanes/ethyl acetate: 100/0 to 30/70) to give the title compound as an oil (58.0 mg, 89%). ^{1}H NMR (300 MHz, $CDCl_3$): δ 12.72 (s, 1H), 8.00 (d, J = 8.2 Hz, 1H), 7.67–7.55 (m, 2H), 7.44 (t, J = 7.9 Hz, 1H), 7.31–7.22 (m, 1H), 7.20–7.12 (m, 1H), 7.05 (d, J = 8.3 Hz, 1H), 7.03–6.97 (m, 1H), 3.79 (s, 3H); ^{13}C NMR (75 MHz, $CDCl_3$): δ 160.1, 145.4, 141.7, 134.8, 130.0, 126.7, 121.3, 120.9, 120.8, 120.3, 114.2, 113.0, 110.5, 55.3; ESI–MS: *m/z* 224.

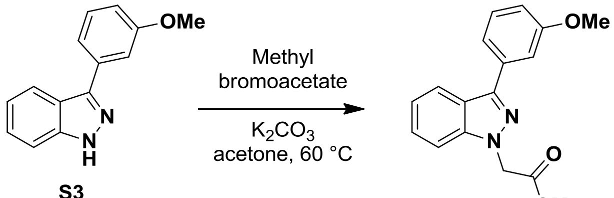

S3

ML212 (36)

ML212; methyl 2-(3-(3-methoxyphenyl)-1*H*-indazol-1-yl)acetate (36): 3-(3-methoxyphenyl)-1*H*-indazole (**S3**, 163 mg, 0.73 mmol) was dissolved in acetone (2.2 mL) and treated with methyl bromoacetate (0.21 mL, 2.2 mmol, 3.0 equiv). Finely powdered potassium carbonate (720 mg, 2.2 mmol, 3.0 equiv) was added in a single portion, and the resulting suspension was stirred overnight at 60 °C. The reaction was then cooled to room temperature and filtered through celite with acetone. The clear filtrate was concentrated under reduced pressure to give the crude product as an oil. This material was purified by column chromatography over silica gel (hexanes/ethyl acetate: 100/0 to 70/30) to give the probe ML212 as an oil (186 mg, 86%). ^{1}H NMR (300 MHz, $CDCl_3$): δ 8.03 (d, J = 8.2 Hz, 1H), 7.57–7.49 (m, 2H), 7.42 (obs. q, J = 7.7 Hz, 2H), 7.36 (obs. t, J = 8.1 Hz, 1H), 7.24 (t, J = 7.5 Hz, 1H), 6.98–6.93 (m, 1H), 5.22 (s, 2H), 3.89 (s, 3H), 3.75 (s, 3H); ^{13}C NMR (75 MHz, $CDCl_3$): δ 168.4, 160.0, 145.1, 141.7, 134.6, 129.8, 126.9, 122.2, 121.6, 121.5, 120.1, 114.2, 112.8, 109.0, 55.4, 52.6, 50.3; HRMS (ESI): calculated mass for $C_{17}H_{16}N_2O_3$ [M+H] 297.1234, found 297.1246.

Representative preparation of methyl 3-alkylindazolyl acetates:

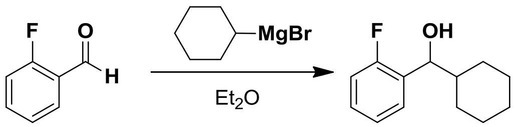

Cyclohexyl (2-fluorophenyl)methanol (S4): 2-Fluorobenzaldehyde (0.25 g, 2.0 mmol) was dissolved in diethyl ether (5.7 mL) and cooled to 0 °C. A 2.0 M solution of cyclohexylmagnesium chloride (1.1 mL, 2.2 mmol, 1.1 equiv) in diethyl ether was added dropwise by syringe, and the resulting clear yellow solution was stirred at room temperature until the reaction was complete by TLC (10 % v/v ethyl acetate/hexanes). The reaction was quenched with a saturated solution of ammonium chloride (aqueous, 2 mL) and further diluted with water (2 mL). The resulting mixture was stirred at room temperature until both layers were clear. The lower aqueous layer was separated and extracted with diethyl ether (2 × 3 mL). The combined organic layers were shaken over magnesium sulfate, filtered, and concentrated under reduced pressure to give a clear, colorless oil, which was used without further purification.

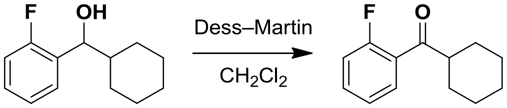

Cyclohexyl (2-fluorophenyl) ketone (S5): The crude cyclohexyl (2-fluorophenyl)methanol **S4** prepared above was dissolved in dichloromethane (2.2 mL), and cooled to 0 °C. The clear, colorless solution was then treated with a 15% w/w solution of Dess-Martin periodinane in dichloromethane (5.9 mL, 2.5 mmol, 1.25 equiv). The reaction was slowly warmed to room temperature and stirred until complete as indicated by TLC (10% v/v ethyl acetate/hexanes). The reaction was quenched with 2 mL saturated, aqueous solution of sodium thiosulfate ($Na_2S_2O_3$) and saturated sodium bicarbonate (aqueous, 2 mL). The layers were separated and the upper aqueous layer was extracted with dichloromethane (2 × 3 mL). The combined organic layers were carefully washed with a saturated solution of sodium bicarbonate (aqueous, 5 mL) then with brine (5 mL). The organic layer was then shaken over magnesium sulfate, filtered, and concentrated under reduced pressure to give an oil. The crude material was

purified by column chromatography over silica gel (hexanes/ethyl acetate: 100/0 to 95/5) to give the title compound as a clear oil (106.2 mg, 26% yield over two steps). ^{1}H NMR (300 MHz, $CDCl_3$): δ 7.74 (td, *J* = 7.6, 1.7 Hz, 1H), 7.53–7.44 (m, 1H), 7.25–7.18 (m, 1H), 7.15–7.07 (m, 1H), 3.18–3.07 (m, 1H), 2.00–1.66 (m, 5H), 1.50–1.23 (m, 5H); ESI–MS: *m/z* 207.

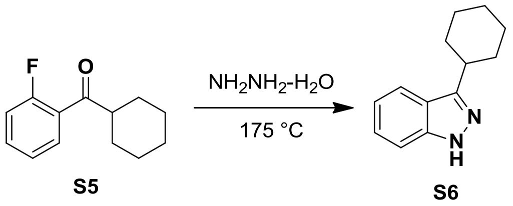

3-Cyclohexyl-1*H*-indazole (S6): Cyclohexyl (2-fluorophenyl)methanone (**S5**, 25 mg, 0.12 mmol) was combined with hydrazine hydrate (120 µL, 2.4 mmol, 20.0 equiv). The resulting clear solution was reacted in the microwave for 4 hours at 175 °C. The reaction mixture was poured into a small quantity of ice (~5 mL) and stirred for 10 minutes at room temperature. The aqueous layer was extracted with dichloromethane (3 × 2 mL). The combined extracts were shaken over magnesium sulfate, filtered, and concentrated under reduced pressure to give a clear oil (16.1 mg, 66%). This material was used immediately without further purification. ^{1}H NMR (300 MHz, $CDCl_3$): δ 10.08 (s, 1H), 7.78 (dd, *J* = 8.1, 0.8 Hz, 1H), 7.42 (dd, *J* = 8.4, 0.8 Hz, 1H), 7.35 (t, *J* = 7.1 Hz, 1H), 7.12 (t, *J* = 7.4 Hz, 1H), 3.09 (tt, *J* = 11.8, 3.4 Hz, 1H), 2.10 (d, *J* = 13.2 Hz, 2H), 1.93–1.86 (m, 2H), 1.84–1.69 (m, 3H), 1.56–1.31 (m, 3H); ESI–MS: *m/z* 201.

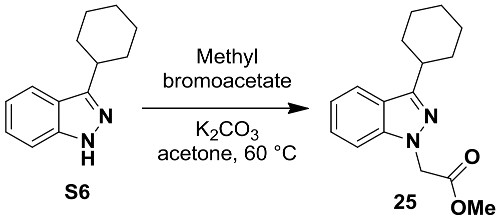

Methyl 2-(3-cyclohexyl-1*H*-indazol-1-yl)acetate (25): Crude 3-cyclohexyl-1*H*-indazole (**S6**, 16 mg, 0.08 mmol) was dissolved in acetone (0.5 mL) and methyl bromoacetate (23 µL, 0.24 mmol, 3.0 equiv). Finely powdered anhydrous potassium carbonate (80 mg, 0.24 mmol, 3.0 equiv) was then added to the clear solution. The mixture was

heated to 60 °C and stirred for 40 hours. The reaction was then cooled to room temperature and filtered through celite with acetone. The clear filtrate was concentrated under reduced pressure to give the crude product as an oil. This material was purified by column chromatography over silica gel (hexanes/ethyl acetate: 100/0 to 85/15) to give the title compound as a white solid (16 mg, 71%). ^{1}H NMR (300 MHz, $CDCl_3$): δ 7.77 (dd, *J* = 8.1, 0.8 Hz, 1H), 7.41–7.33 (m, 1H), 7.28–7.23 (m, 1H), 7.16–7.08 (m, 1H), 5.10 (s, 2H), 3.73 (s, 3H), 3.05 (ddd, *J* = 11.9, 7.7, 3.4 Hz, 1H), 2.08 (d, *J* = 12.4 Hz, 2H), 1.88 (d, *J* = 11.7 Hz, 2H), 1.78 (dd, *J* = 21.6, 9.4 Hz, 3H), 1.54–1.30 (m, 3H); ^{13}C NMR (75 MHz, $CDCl_3$): δ 168.7, 151.4, 141.3, 126.6, 122.5, 121.1, 119.9, 108.7, 52.4, 50.0, 37.7, 32.6, 26.7, 26.2; ESI–MS: *m/z* 273.

NMR and MS data for reported compounds

General details: NMR spectra were recorded on a Bruker 300 MHz spectrometer. Proton and carbon chemical shifts are reported in ppm (δ) relative to tetramethylsilane (^{1}H δ 0.00) or residual chloroform in $CDCl_3$ solvent (^{1}H δ 7.24, ^{13}C δ 77.0). NMR data are reported as follows: chemical shifts, multiplicity (obs. = obscured, br = broad, s = singlet, d = doublet, t = triplet, q = quartet, m = multiplet); coupling constant(s) in Hz; integration.

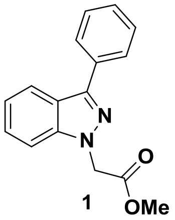

1

^{1}H NMR (300 MHz, $CDCl_3$): δ 8.00 (d, *J* = 8.2 Hz, 1H), 7.97–7.92 (m, 2H), 7.47 (t, *J* = 7.4 Hz, 2H), 7.37 (ddt, *J* = 5.2, 4.4, 3.1 Hz, 2H), 7.29 (d, *J* = 8.4 Hz, 1H), 7.23–7.16 (m, 1H), 5.16 (s, 2H), 3.69 (s, 3H); ^{13}C NMR (75 MHz, $CDCl_3$): δ 168.3, 145.0, 141.6, 133.2, 128.7, 128.0, 127.5, 126.7, 122.0, 121.4, 121.3, 108.9, 52.4, 50.1; ESI–MS: *m/z* 267.

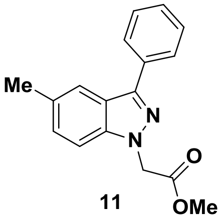

11

^{1}H NMR (300 MHz, $CDCl_3$): δ 7.94 (d, *J* = 7.6 Hz, 2H), 7.79 (s, 1H), 7.50 (t, *J* = 7.6 Hz, 2H), 7.39 (t, *J* = 7.3 Hz, 1H), 7.26 (d, *J* = 3.0 Hz, 2H), 5.19 (s, 2H), 3.74 (s, 3H), 2.49 (s, 3H); ESI–MS: *m/z* 281.

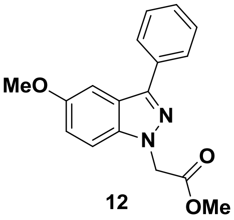

12

^{1}H NMR (300 MHz, $CDCl_3$): δ 7.96–7.88 (m, 2H), 7.54–7.46 (m, 2H), 7.44–7.36 (m, 1H), 7.34 (d, *J* = 1.6 Hz, 1H), 7.25 (d, *J* = 9.0 Hz, 1H), 7.11 (dd, *J* = 9.0, 2.2 Hz, 1H), 5.18 (s, 2H), 3.87 (s, 3H), 3.74 (s, 3H); ^{13}C NMR (75 MHz, $CDCl_3$): δ 168.4, 155.3, 144.5, 137.5, 133.4, 128.8, 127.9, 127.4, 122.3, 118.9, 110.0, 100.9, 55.8, 52.6, 50.4; ESI–MS: *m/z* 297.

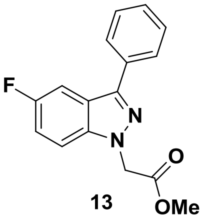

13

^{1}H NMR (300 MHz, $CDCl_3$): δ 7.94–7.86 (m, 2H), 7.71–7.61 (m, 1H), 7.55–7.45 (m, 2H), 7.45–7.37 (m, 1H), 7.30 (dd, *J* = 9.0, 4.2 Hz, 1H), 7.25–7.18 (m, 1H), 5.20 (s, 2H), 3.76 (s, 3H); ESI–MS: *m/z* 285.

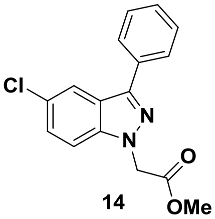

14

^{1}H NMR (300 MHz, $CDCl_3$): δ 8.00–7.98 (m, 1H), 7.92–7.88 (m, 2H), 7.54–7.47 (m, 2H), 7.45–7.36 (m, 2H), 7.28 (d, *J* = 8.9 Hz, 1H), 5.19 (s, 2H), 3.76 (s, 3H); ^{13}C NMR (75 MHz, $CDCl_3$): δ 168.1, 144.8, 140.2, 132.6, 128.9, 128.4, 127.5, 127.5, 127.3, 123.0, 120.8, 110.2, 52.7, 50.4; ESI–MS: *m/z* 301.

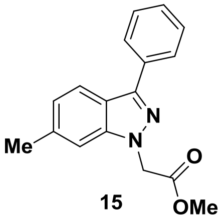

15

^{1}H NMR (300 MHz, $CDCl_3$): δ 7.95 (d, *J* = 8.1 Hz, 2H), 7.89 (d, *J* = 8.3 Hz, 1H), 7.49 (t, *J* = 7.6 Hz, 2H), 7.39 (t, *J* = 7.3 Hz, 1H), 7.12 (s, 1H), 7.07 (d, *J* = 8.4 Hz, 1H), 5.18 (s, 2H), 3.76 (s, 3H), 2.51 (s, 3H); ESI–MS: *m/z* 281.

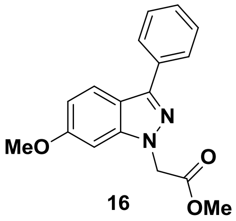

16

^{1}H NMR (300 MHz, $CDCl_3$): δ 7.96–7.88 (m, 2H), 7.51 (t, *J* = 7.4 Hz, 2H), 7.43–7.36 (m, 1H), 7.34 (d, *J* = 2.0 Hz, 1H), 7.25 (d, *J* = 9.0 Hz, 1H), 7.12 (dd, *J* = 9.0, 2.3 Hz, 1H), 5.18 (s, 2H), 3.88 (s, 3H), 3.75 (s, 3H); ESI–MS: *m/z* 297.

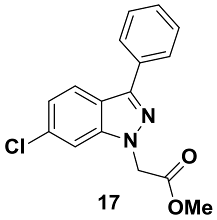

17

^{1}H NMR (300 MHz, $CDCl_3$): δ 7.96–7.88 (m, 3H), 7.50 (t, *J* = 7.3 Hz, 2H), 7.42 (dd, *J* = 10.4, 4.2 Hz, 1H), 7.36 (s, 1H), 7.21 (dd, *J* = 8.7, 1.5 Hz, 1H), 5.17 (s, 2H), 3.78 (s, 3H); ESI–MS: *m/z* 301.

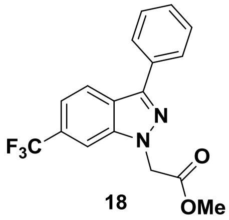

18

^{1}H NMR (300 MHz, $CDCl_3$): δ 8.13 (d, *J* = 8.5 Hz, 1H), 7.93 (d, *J* = 7.4 Hz, 2H), 7.66 (s, 1H), 7.56–7.40 (m, 4H), 5.26 (s, 2H), 3.79 (s, 3H); ^{13}C NMR (75 MHz, $CDCl_3$): δ 167.9, 145.5, 140.7, 132.5, 129.1 (q, *J* = 32.4 Hz), 128.9, 128.6, 127.6, 126.1, 123.9, 122.6, 118.0 (q, *J* = 3.2 Hz), 106.9 (q, *J* = 4.5 Hz), 52.7, 50.4; ESI–MS: *m/z* 335.

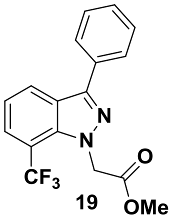

19

^{1}H NMR (300 MHz, $CDCl_3$): δ 8.21 (d, *J* = 8.2 Hz, 1H), 7.92–7.86 (m, 2H), 7.78 (d, *J* = 7.4 Hz, 1H), 7.57–7.41 (m, 3H), 7.29 (t, *J* = 7.8 Hz, 1H), 5.42 (s, 2H), 3.73 (s, 3H); ^{13}C NMR (75 MHz, $CDCl_3$): δ 168.6, 146.4, 137.1, 132.1, 128.9, 128.7, 128.1, 126.3, 125.6 (q, *J* = 6.0 Hz), 125.1, 122.1, 120.4, 112.6 (q, *J* = 33.0 Hz), 52.7 (q, *J* = 4.6 Hz), 52.4; ESI–MS: *m/z* 335.

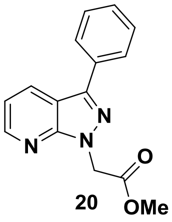

20

^{1}H NMR (300 MHz, $CDCl_3$): δ 8.58 (dd, *J* = 4.5, 1.3 Hz, 1H), 8.37 (dd, *J* = 8.1, 1.3 Hz, 1H), 8.01–7.93 (m, 2H), 7.52 (t, *J* = 7.4 Hz, 2H), 7.44 (d, *J* = 7.3 Hz, 1H), 7.23 (dd, *J* = 8.1, 4.6 Hz, 1H), 5.39 (s, 2H), 3.77 (s, 3H); ESI–MS: *m/z* 268.

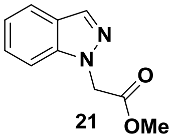

21

^{1}H NMR (300 MHz, $CDCl_3$): δ 8.05 (s, 1H), 7.74 (d, *J* = 8.1 Hz, 1H), 7.43–7.35 (m, 1H), 7.31 (d, *J* = 8.4 Hz, 1H), 7.17 (ddd, *J* = 7.7, 6.8, 0.9 Hz, 1H), 5.16 (s, 2H), 3.72 (s, 3H); ^{13}C NMR (75 MHz, $CDCl_3$): δ 168.4, 140.1, 134.3, 126.8, 124.3, 121.2, 120.9, 108.6, 52.4, 50.1; ESI–MS: *m/z* 191.

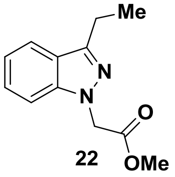

22

^{1}H NMR (300 MHz, $CDCl_3$): δ 7.71 (d, *J* = 7.3 Hz, 1H), 7.39 (dd, *J* = 8.2, 7.1 Hz, 1H), 7.27 (obs. d, *J* = 7.9 Hz, 1H), 7.15 (t, *J* = 7.4 Hz, 1H), 5.11 (s, 2H), 3.74 (s, 3H), 3.01 (q, *J* = 7.6 Hz, 2H), 1.41 (t, *J* = 7.6 Hz, 3H); ^{13}C NMR (75 MHz, $CDCl_3$): δ 168.7, 148.3, 141.2, 126.7, 123.1, 120.7, 120.2, 108.7, 52.5, 49.9, 20.5, 13.5; ESI–MS: *m/z* 219.

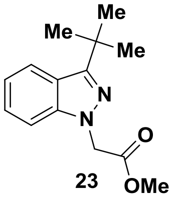

23

^{1}H NMR (300 MHz, $CDCl_3$): δ 7.89 (d, *J* = 8.2 Hz, 1H), 7.36 (dd, *J* = 8.1, 7.1 Hz, 1H), 7.25–7.23 (m, 1H), 7.12 (t, *J* = 7.5 Hz, 1H), 5.10 (s, 2H), 3.74 (s, 3H), 1.53 (s, 9H); ESI–MS: *m/z* 247.

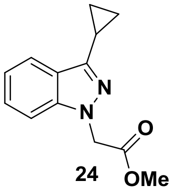

24

^{1}H NMR (300 MHz, $CDCl_3$): δ 7.74 (dd, *J* = 8.1, 0.7 Hz, 1H), 7.37 (dd, *J* = 8.1, 7.2 Hz, 1H), 7.24 (d, *J* = 9.4 Hz, 1H), 7.13 (t, *J* = 7.5 Hz, 1H), 5.06 (s, 2H), 3.72 (s, 3H), 2.31–

2.13 (m, 1H), 1.11–0.99 (m, 4H); ^{13}C NMR (75 MHz, $CDCl_3$): δ 168.7, 148.0, 141.3, 126.8, 123.4, 120.6, 120.1, 108.7, 52.4, 49.9, 8.2, 7.0; ESI–MS: *m/z* 231.

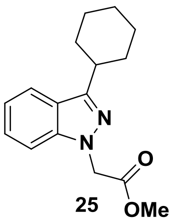

25

^{1}H NMR (300 MHz, $CDCl_3$): δ 7.77 (dd, *J* = 8.1, 0.8 Hz, 1H), 7.41–7.33 (m, 1H), 7.28–7.23 (m, 1H), 7.16–7.08 (m, 1H), 5.10 (s, 2H), 3.73 (s, 3H), 3.05 (ddd, *J* = 11.9, 7.7, 3.4 Hz, 1H), 2.08 (d, *J* = 12.4 Hz, 2H), 1.88 (d, *J* = 11.7 Hz, 2H), 1.78 (dd, *J* = 21.6, 9.4 Hz, 3H), 1.54–1.30 (m, 3H); ^{13}C NMR (75 MHz, $CDCl_3$): δ 168.7, 151.4, 141.3, 126.6, 122.5, 121.1, 119.9, 108.7, 52.4, 50.0, 37.7, 32.6, 26.7, 26.2; ESI–MS: *m/z* 273.

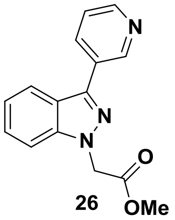

26

^{1}H NMR (300 MHz, $CDCl_3$): δ 9.23 (d, *J* = 1.5 Hz, 1H), 8.64 (dd, *J* = 4.8, 1.6 Hz, 1H), 8.29–8.23 (m, 1H), 8.01 (d, *J* = 8.2 Hz, 1H), 7.51–7.36 (m, 3H), 7.32–7.26 (m, 1H), 5.24 (s, 2H), 3.77 (s, 3H); ESI–MS: *m/z* 268.

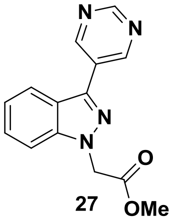

27

^{1}H NMR (300 MHz, $CDCl_3$): δ 9.34 (s, 2H), 9.25 (s, 1H), 7.98 (d, *J* = 8.2 Hz, 1H), 7.51 (t, *J* = 7.5 Hz, 1H), 7.42 (d, *J* = 8.5 Hz, 1H), 7.33 (ddd, *J* = 7.8, 7.2, 0.8 Hz, 1H), 5.26 (s, 2H), 3.79 (s, 3H); ^{13}C NMR (75 MHz, $CDCl_3$): δ 168.0, 157.8, 154.9, 141.7, 138.9, 127.7, 127.5, 122.5, 121.9, 120.6, 109.5, 52.7, 50.4; ESI–MS: *m/z* 269.

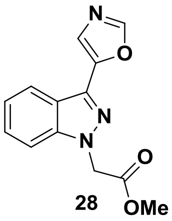

28

^{1}H NMR (300 MHz, $CDCl_3$): δ 8.03 (s, 1H), 8.02 (obs. d, *J* = 6.0 Hz, 1H), 7.63 (s, 1H), 7.49 (dd, *J* = 11.1, 4.2 Hz, 1H), 7.41–7.28 (m, 2H), 5.23 (s, 2H), 3.77 (s, 3H); ESI–MS: *m/z* 258.

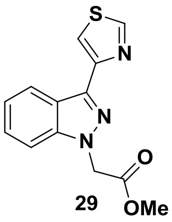

29

^{1}H NMR (300 MHz, $CDCl_3$): δ 8.97 (dd, *J* = 2.0, 0.8 Hz, 1H), 8.38 (dd, *J* = 8.2, 0.9 Hz, 1H), 7.91 (dd, *J* = 1.9, 0.7 Hz, 1H), 7.53–7.41 (m, 1H), 7.38–7.28 (m, 2H), 5.24 (s, 2H), 3.76 (s, 3H); ESI–MS: *m/z* 274.

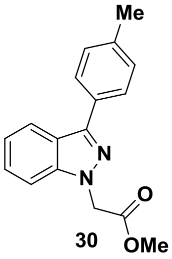

30

^{1}H NMR (300 MHz, $CDCl_3$): δ 8.01 (d, *J* = 8.2 Hz, 1H), 7.85 (d, *J* = 8.1 Hz, 2H), 7.47–7.38 (m, 1H), 7.33 (d, *J* = 8.9 Hz, 2H), 7.27–7.18 (m, 1H), 5.21 (s, 2H), 3.75 (s, 3H), 2.42 (s, 3H); ^{13}C NMR (75 MHz, $CDCl_3$): δ 168.5, 145.3, 141.6, 137.9, 130.4, 129.5, 127.5, 126.8, 122.2, 121.6, 121.3, 108.9, 52.5, 50.2, 21.3; ESI–MS: *m/z* 281.

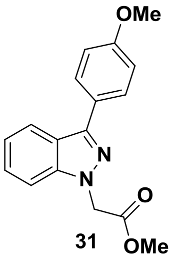

31

^{1}H NMR (300 MHz, $CDCl_3$): δ 7.99 (d, *J* = 8.2 Hz, 1H), 7.89 (d, *J* = 8.7 Hz, 2H), 7.42 (t, *J* = 7.5 Hz, 1H), 7.32 (d, *J* = 8.4 Hz, 1H), 7.22 (t, *J* = 7.5 Hz, 1H), 7.03 (d, *J* = 8.7 Hz, 2H), 5.19 (s, 2H), 3.86 (s, 3H), 3.74 (s, 3H); ^{13}C NMR (75 MHz, $CDCl_3$): δ 168.5, 159.6, 145.0, 141.6, 128.8, 126.8, 125.9, 122.1, 121.6, 121.2, 114.2, 108.9, 55.3, 52.5, 50.2; ESI–MS: *m/z* 297.

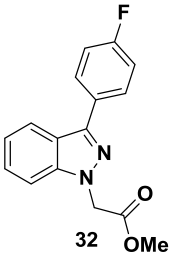

32

^{1}H NMR (300 MHz, $CDCl_3$): δ 8.00–7.87 (m, 3H), 7.43 (dd, *J* = 8.1, 7.1 Hz, 1H), 7.34 (d, *J* = 8.4 Hz, 1H), 7.28–7.11 (m, 3H), 5.20 (s, 2H), 3.75 (s, 3H); ^{13}C NMR (75 MHz, $CDCl_3$): δ 168.3, 164.4, 161.1, 144.2, 141.6, 129.4 (d, *J* = 3.3 Hz), 129.3, 129.2, 126.9, 121.9, 121.4 (d, *J* = 19.2 Hz), 115.7 (d, *J* = 21.6 Hz), 109.0, 52.5, 50.2; ESI–MS: *m/z* 285.

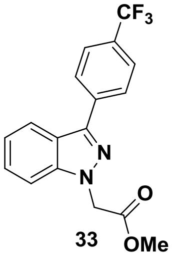

33

^{1}H NMR (300 MHz, $CDCl_3$): δ 8.08 (d, *J* = 8.0 Hz, 2H), 8.01 (d, *J* = 8.2 Hz, 1H), 7.74 (d, *J* = 8.1 Hz, 2H), 7.50–7.42 (m, 1H), 7.37 (d, *J* = 8.4 Hz, 1H), 7.32–7.25 (m, 1H), 5.23 (s, 2H), 3.76 (s, 3H); ^{13}C NMR (75 MHz, $CDCl_3$): δ 168.2, 143.6, 141.8, 136.9, 129.9 (q, *J* =

32.4 Hz), 127.6, 127.1, 126.0, 125.7 (q, J = 3.8 Hz), 122.4, 122.0, 121.1, 109.2, 52.6, 50.3; ESI–MS: *m/z* 335.

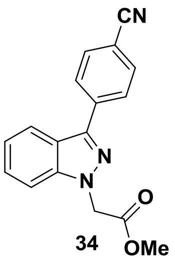

34

^{1}H NMR (300 MHz, $CDCl_3$): δ 8.10 (d, J = 8.0 Hz, 2H), 8.00 (d, J = 8.2 Hz, 1H), 7.77 (d, J = 8.0 Hz, 2H), 7.51–7.45 (m, 1H), 7.39 (d, J = 8.4 Hz, 1H), 7.30 (dd, J = 7.9, 7.0 Hz, 1H), 5.24 (s, 2H), 3.78 (s, 3H); ESI–MS: *m/z* 292.

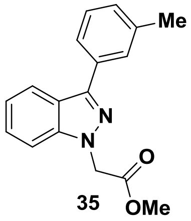

35

^{1}H NMR (300 MHz, $CDCl_3$): δ 8.03 (d, J = 8.2 Hz, 1H), 7.78–7.72 (m, 2H), 7.49–7.33 (m, 3H), 7.30–7.19 (m, 2H), 5.22 (s, 2H), 3.75 (s, 3H), 2.45 (s, 3H); ^{13}C NMR (75 MHz, $CDCl_3$): δ 168.4, 145.4, 141.7, 138.5, 133.2, 128.9, 128.6, 128.2, 126.8, 124.8, 122.2, 121.7, 121.4, 108.9, 52.5, 50.3, 21.5; ESI–MS: *m/z* 281.

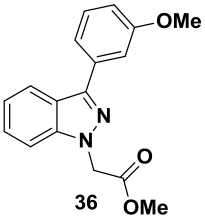

36

^{1}H NMR (300 MHz, $CDCl_3$): δ 8.03 (d, J = 8.2 Hz, 1H), 7.57–7.49 (m, 2H), 7.42 (obs. q, J = 7.7 Hz, 2H), 7.36 (obs. t, J = 8.1 Hz, 1H), 7.24 (t, J = 7.5 Hz, 1H), 6.98–6.93 (m, 1H), 5.22 (s, 2H), 3.89 (s, 3H), 3.75 (s, 3H); ^{13}C NMR (75 MHz, $CDCl_3$): δ 168.4, 160.0, 145.1, 141.7, 134.6, 129.8, 126.9, 122.2, 121.6, 121.5, 120.1, 114.2, 112.8, 109.0, 55.4, 52.6, 50.3; ESI–MS: *m/z* 297.

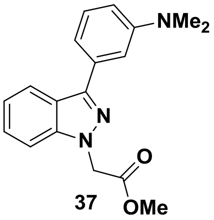

37

^{1}H NMR (300 MHz, $CDCl_3$): δ 8.04 (d, *J* = 8.2 Hz, 1H), 7.49–7.17 (m, 6H), 6.89–6.74 (m, 1H), 5.22 (s, 2H), 3.75 (s, 3H), 3.03 (s, 6H); ^{13}C NMR (75 MHz, $CDCl_3$): δ 168.5, 151.0, 146.1, 141.6, 133.8, 129.4, 126.7, 122.4, 121.8, 121.2, 116.3, 112.7, 111.8, 108.9, 52.5, 50.3, 40.7; ESI–MS: *m/z* 310.

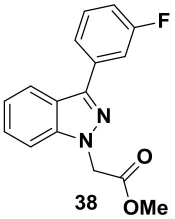

38

^{1}H NMR (300 MHz, $CDCl_3$): δ 8.01 (d, *J* = 8.2 Hz, 1H), 7.75 (d, *J* = 7.7 Hz, 1H), 7.71–7.64 (m, 1H), 7.49–7.40 (m, 2H), 7.35 (d, *J* = 8.4 Hz, 1H), 7.30–7.21 (m, 1H), 7.14–7.04 (m, 1H), 5.21 (s, 2H), 3.75 (s, 3H); ^{13}C NMR (75 MHz, $CDCl_3$): δ 168.2, 163.1 (d, *J* = 243.75 Hz), 143.9, 143.9, 141.7, 135.4 (d, *J* = 8.3 Hz), 130.3 (d, *J* = 8.4 Hz), 127.0, 123.1 (d, *J* = 2.9 Hz), 121.9, 121.7, 121.2, 114.8 (d, *J* = 21.2 Hz), 114.3 (d, *J* = 22.7 Hz), 109.1, 52.6, 50.3; ESI–MS: *m/z* 285.

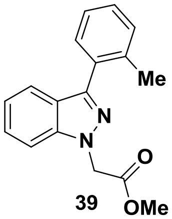

39

^{1}H NMR (300 MHz, $CDCl_3$): δ 7.66 (d, *J* = 8.1 Hz, 1H), 7.53 (d, *J* = 7.2 Hz, 1H), 7.48–7.30 (m, 5H), 7.19 (t, *J* = 7.4 Hz, 1H), 5.22 (s, 2H), 3.75 (s, 3H), 2.39 (s, 3H); ^{13}C NMR (75 MHz, $CDCl_3$): δ 168.5, 145.9, 141.0, 137.4, 131.8, 130.7, 130.4, 128.3, 126.9, 125.6, 123.5, 121.6, 121.1, 108.8, 52.5, 50.2, 20.4; ESI–MS: *m/z* 281.

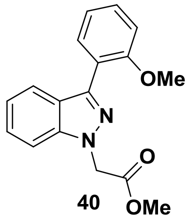

40

^{1}H NMR (300 MHz, $CDCl_3$): δ 7.75 (d, *J* = 8.2 Hz, 1H), 7.66–7.61 (m, 1H), 7.44–7.35 (m, 2H), 7.32 (d, *J* = 8.4 Hz, 1H), 7.21–7.12 (m, 1H), 7.11–7.00 (m, 2H), 5.21 (s, 2H), 3.82 (s, 3H), 3.73 (s, 3H); ^{13}C NMR (75 MHz, $CDCl_3$): δ 168.5, 157.3, 143.7, 141.1, 131.5, 129.8, 126.6, 123.6, 122.8, 122.1, 120.8, 120.7, 111.3, 108.6, 55.5, 52.5, 50.3; ESI–MS: *m/z* 297.

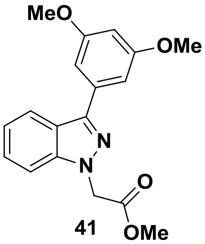

41

^{1}H NMR (300 MHz, $CDCl_3$): δ 8.03 (dd, *J* = 8.2, 0.8 Hz, 1H), 7.48–7.39 (m, 1H), 7.34 (dd, *J* = 8.4, 0.8 Hz, 1H), 7.24 (ddd, *J* = 8.0, 6.1, 0.9 Hz, 1H), 7.14–7.09 (m, 2H), 6.52 (t, *J* = 2.2 Hz, 1H), 5.21 (s, 2H), 3.87 (s, 6H), 3.75 (s, 3H); ^{13}C NMR (75 MHz, $CDCl_3$): δ 168.4, 161.1, 145.0, 141.7, 135.0, 126.9, 122.1, 121.6, 121.5, 109.0, 105.7, 100.6, 55.5, 52.5, 50.3; ESI–MS: *m/z* 327.
