# Supplementary material for: ML212: A small-molecule probe for investigating fluconazole resistance mechanisms in Candida albicans
Source: Beilstein J Org Chem. 2013 Jul 26;9:1501–7. doi: 10.3762/bjoc.9.171 (PMC3740683; doi:10.3762/bjoc.9.171)
Supplement: File 2 — NMR spectra of reported compounds. [file Beilstein_J_Org_Chem-09-1501-s002.pdf]

Supporting Information

for

ML212: A small-molecule probe for investigating fluconazole resistance mechanisms in *Candida albicans*

Willmen Youngsaye[1], Cathy L. Hartland[1], Barbara J. Morgan[1], Amal Ting[1], Partha P. Nag[1], Benjamin Vincent[2,3], Carrie A. Mosher[1], Joshua A. Bittker[1], Sivaraman Dandapani[1], Michelle Palmer[1], Luke Whitesell[2], Susan Lindquist[2,4], Stuart L. Schreiber[1,5] and Benito Munoz[1,*]

Address: [1]Chemical Biology Platform and Probe Development Center, Broad Institute of MIT and Harvard, 7 Cambridge Center, Cambridge, MA 02142, USA, [2]Whitehead Institute for Biomedical Research, 9 Cambridge Center, Cambridge, MA 02142, USA, [3]Microbiology Graduate Program, Massachusetts Institute of Technology, 77 Massachusetts Avenue, Cambridge, MA 02139, USA, [4]Department of Biology and Howard Hughes Medical Institute, Massachusetts Institute of Technology, 77 Massachusetts Avenue, Cambridge, MA 02139, USA and [5]Howard Hughes Medical Institute, Broad Institute of Harvard and MIT, 7 Cambridge Center, Cambridge, MA 02142, USA

Email: Benito Munoz - bmunoz@broadinstitute.org

* Corresponding author

NMR spectra of reported compounds

^1H NMR (300 MHz, $CDCl_3$)

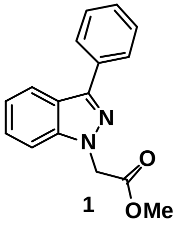

1

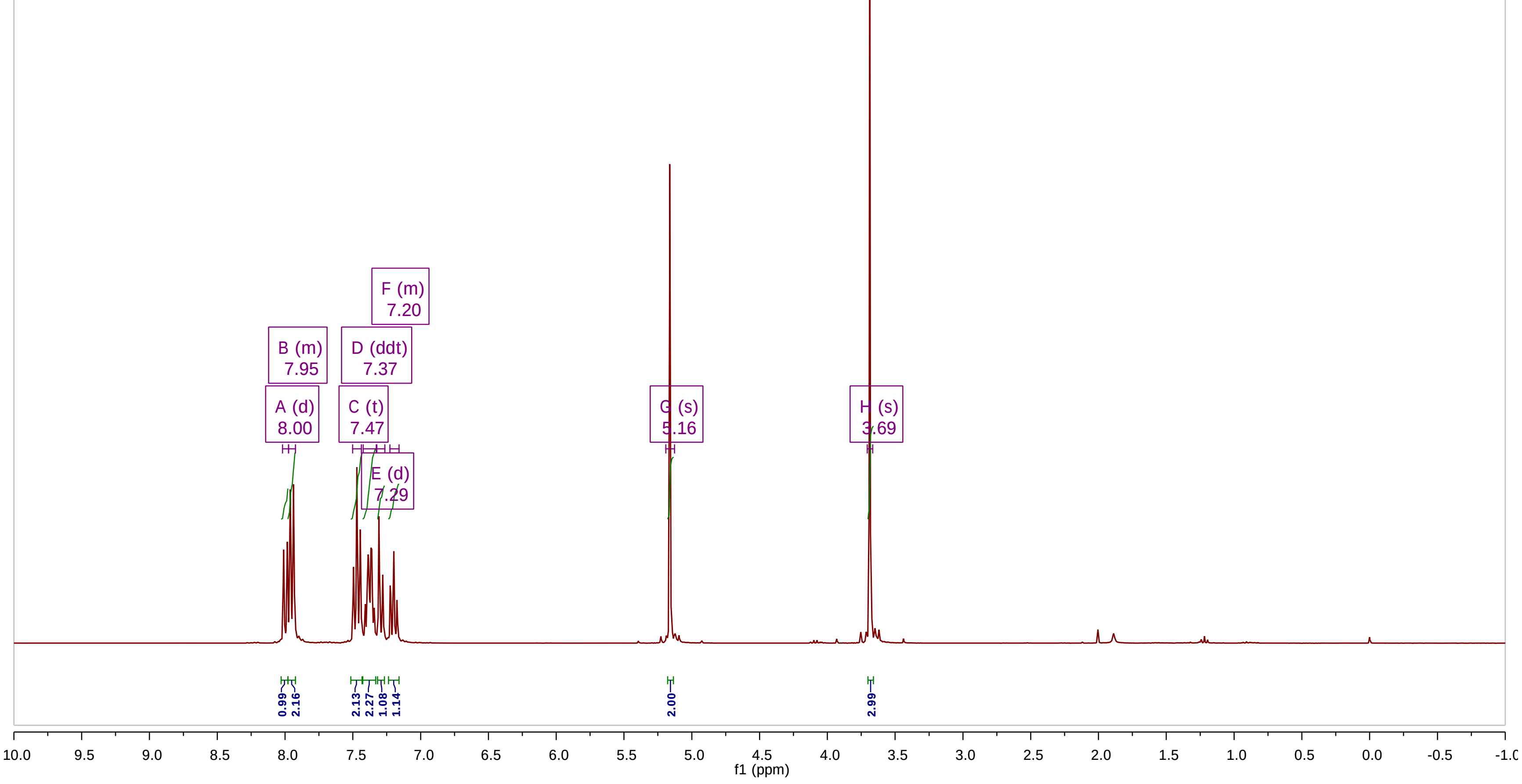

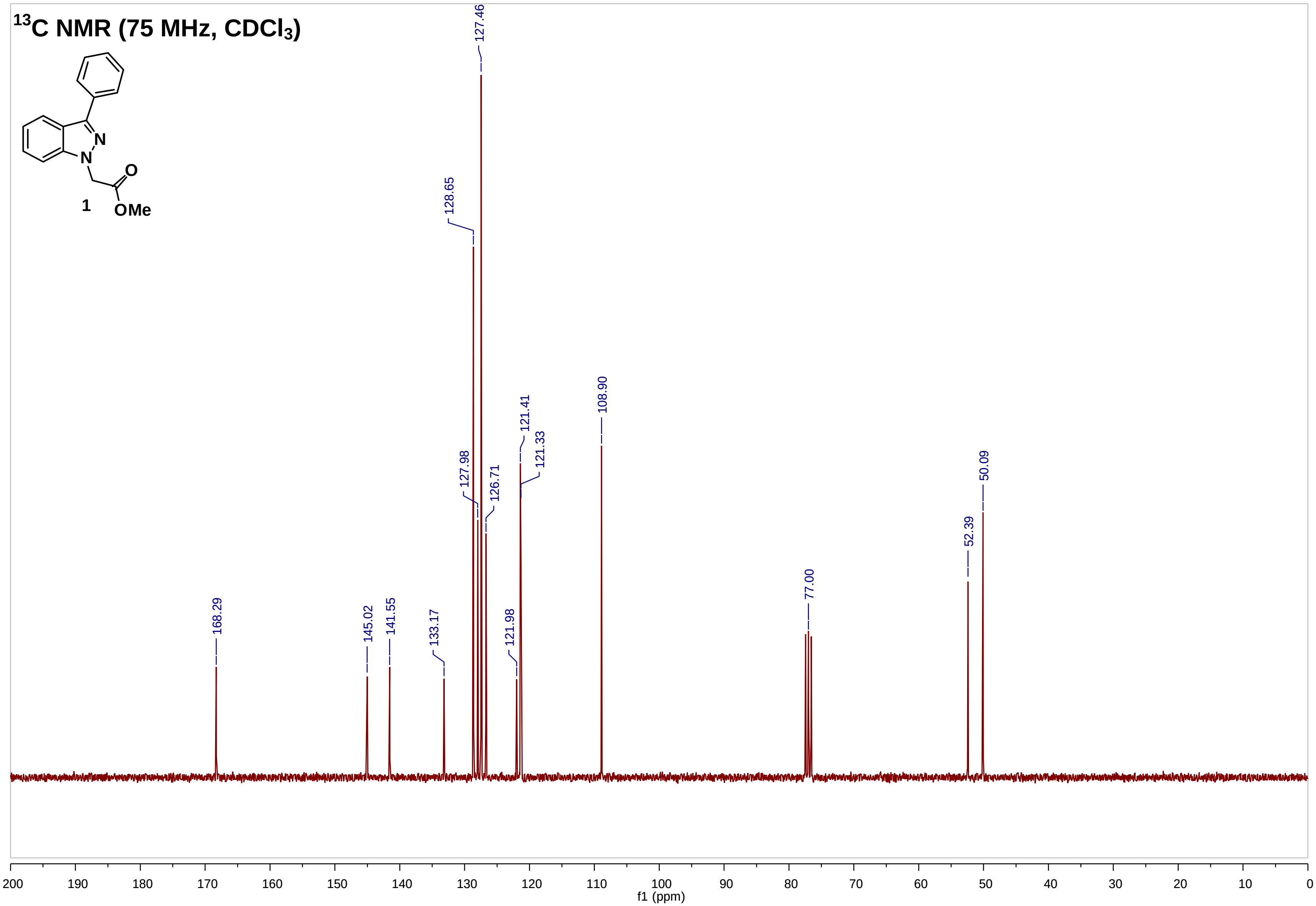

13C NMR (75 MHz, CDCl3)
N
N
O
OMe
1
127.46
128.65
127.98
126.71
121.41
121.33
108.90
50.09
52.39
77.00
168.29
145.02
141.55
133.17
121.98
200
190
180
170
160
150
140
130
120
110
100
90
80
70
60
50
40
30
20
10
0
f1 (ppm)

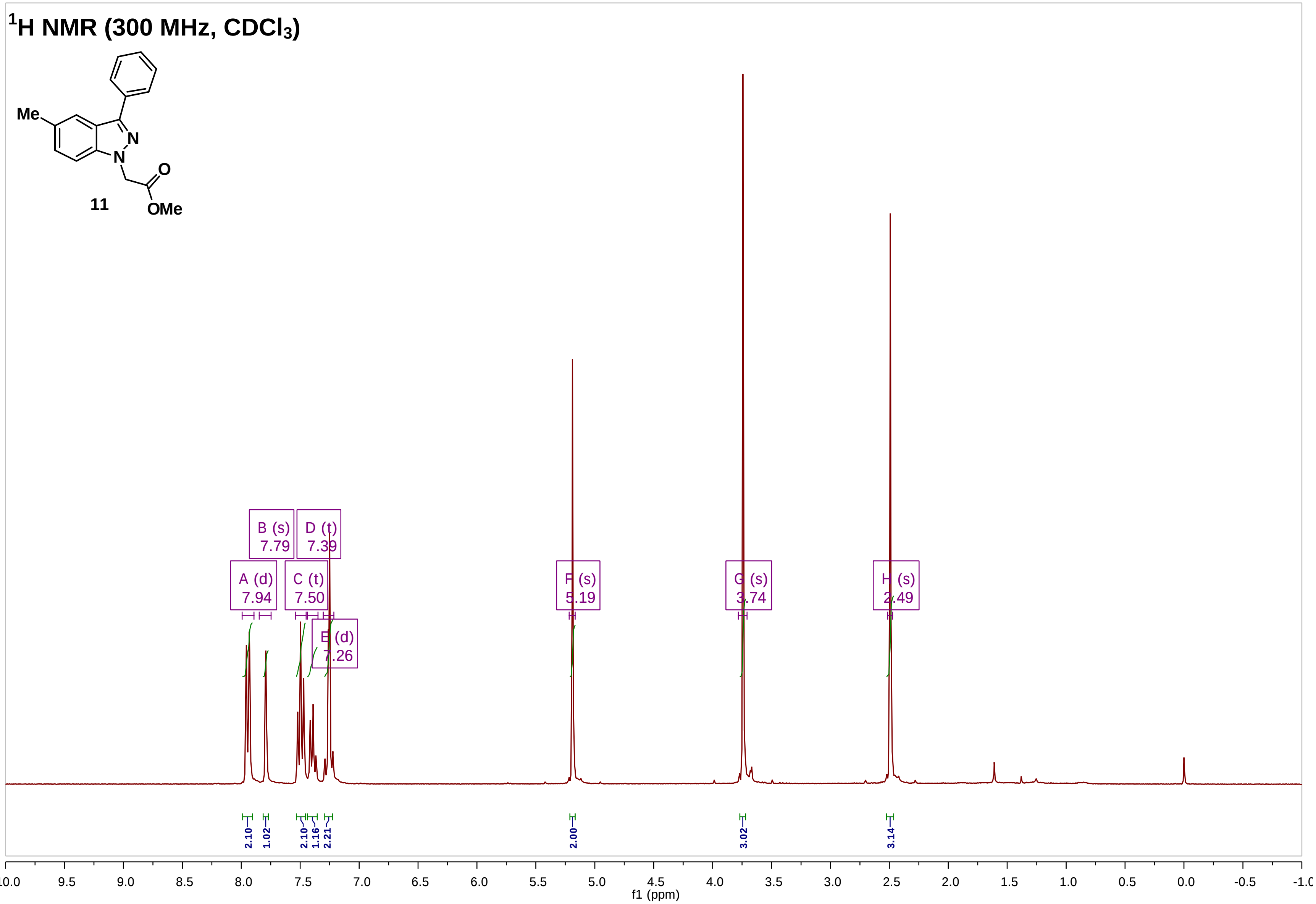

1H NMR (300 MHz, CDCl3)
Me
N
N
O
11
OMe
A (d)
7.94
B (s)
7.79
C (t)
7.50
D (t)
7.39
E (d)
7.26
F (s)
5.19
G (s)
3.74
H (s)
2.49
2.10
1.02
2.10
1.16
2.21
2.00
3.02
3.14
10.0
9.5
9.0
8.5
8.0
7.5
7.0
6.5
6.0
5.5
5.0
4.5
4.0
3.5
3.0
2.5
2.0
1.5
1.0
0.5
0.0
-0.5
-1.0
f1 (ppm)

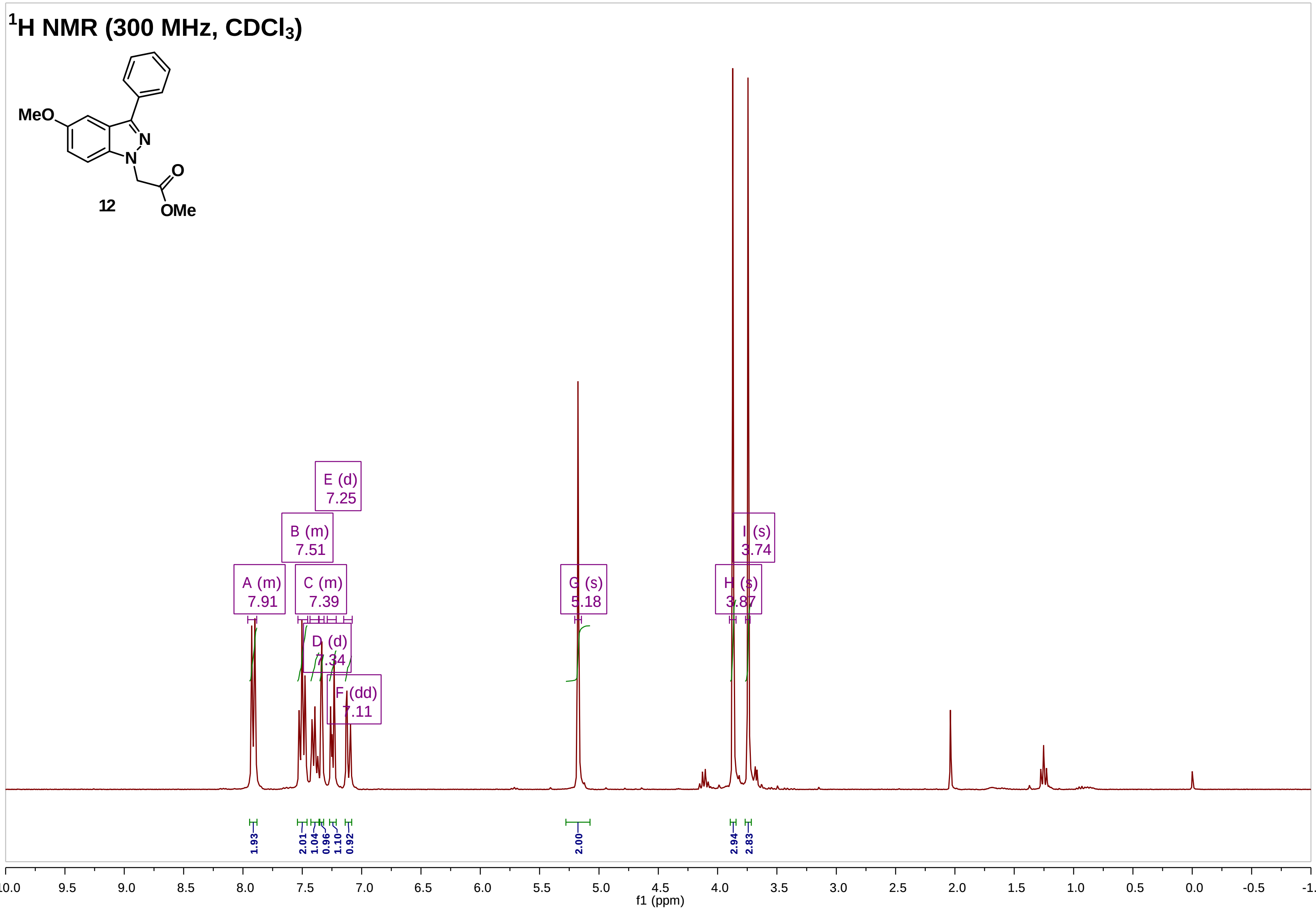

^{1}H NMR (300 MHz, CDCl$_3$)
MeO
N
N
O
OMe
12
A (m)
7.91
B (m)
7.51
C (m)
7.39
D (d)
7.34
E (d)
7.25
F (dd)
7.11
G (s)
5.18
H (s)
3.87
I (s)
3.74
1.93
2.01
1.04
0.96
1.10
0.92
2.00
2.94
2.83
10.0
9.5
9.0
8.5
8.0
7.5
7.0
6.5
6.0
5.5
5.0
4.5
4.0
3.5
3.0
2.5
2.0
1.5
1.0
0.5
0.0
-0.5
f1 (ppm)

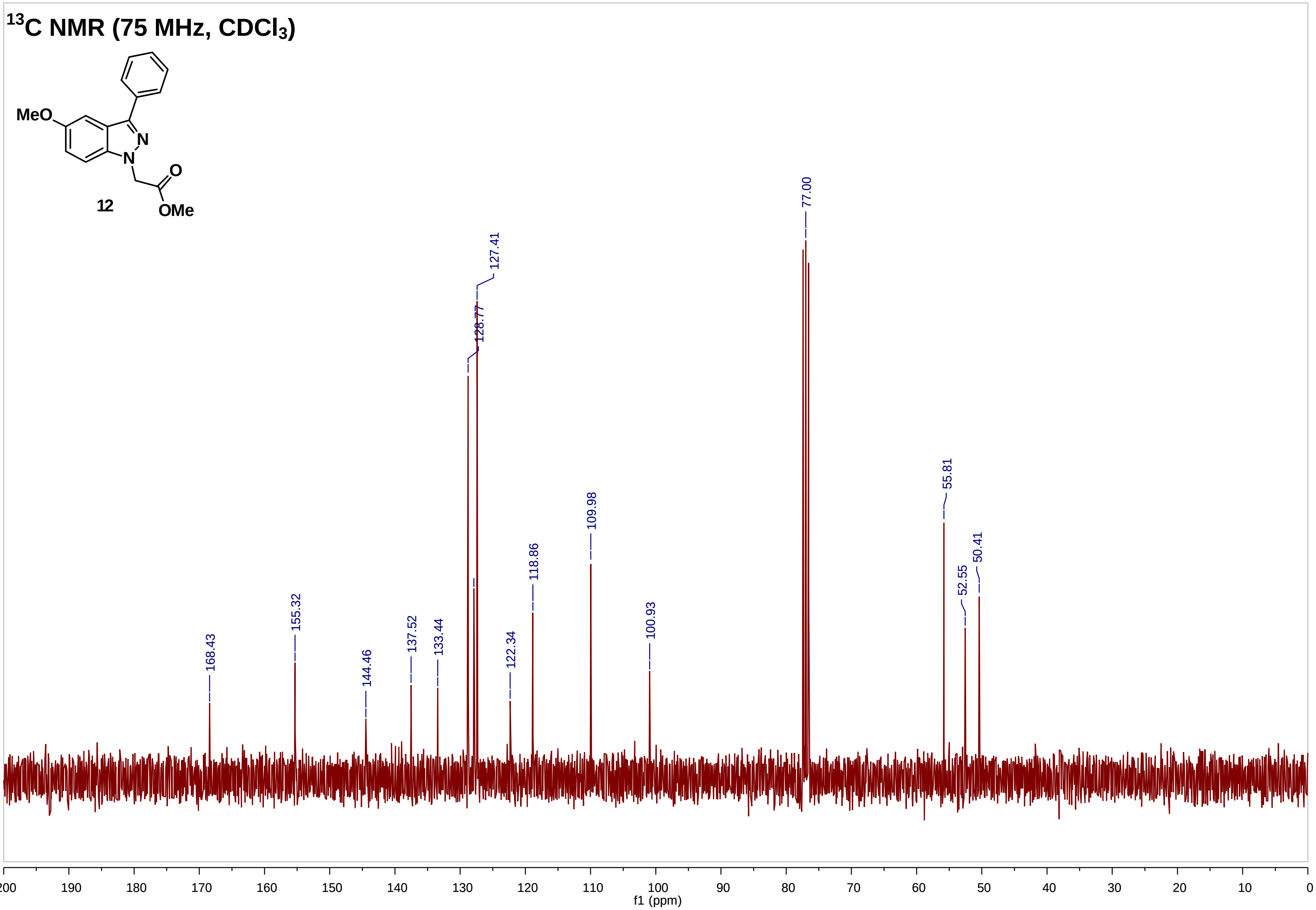

^{13}C NMR (75 MHz, $CDCl_3$)
MeO
N
N
O
OMe
12
168.43
155.32
144.46
137.52
133.44
128.77
127.41
122.34
118.86
109.98
100.93
77.00
55.81
52.55
50.41
200
190
180
170
160
150
140
130
120
110
100
90
80
70
60
50
40
30
20
10
0
f1 (ppm)

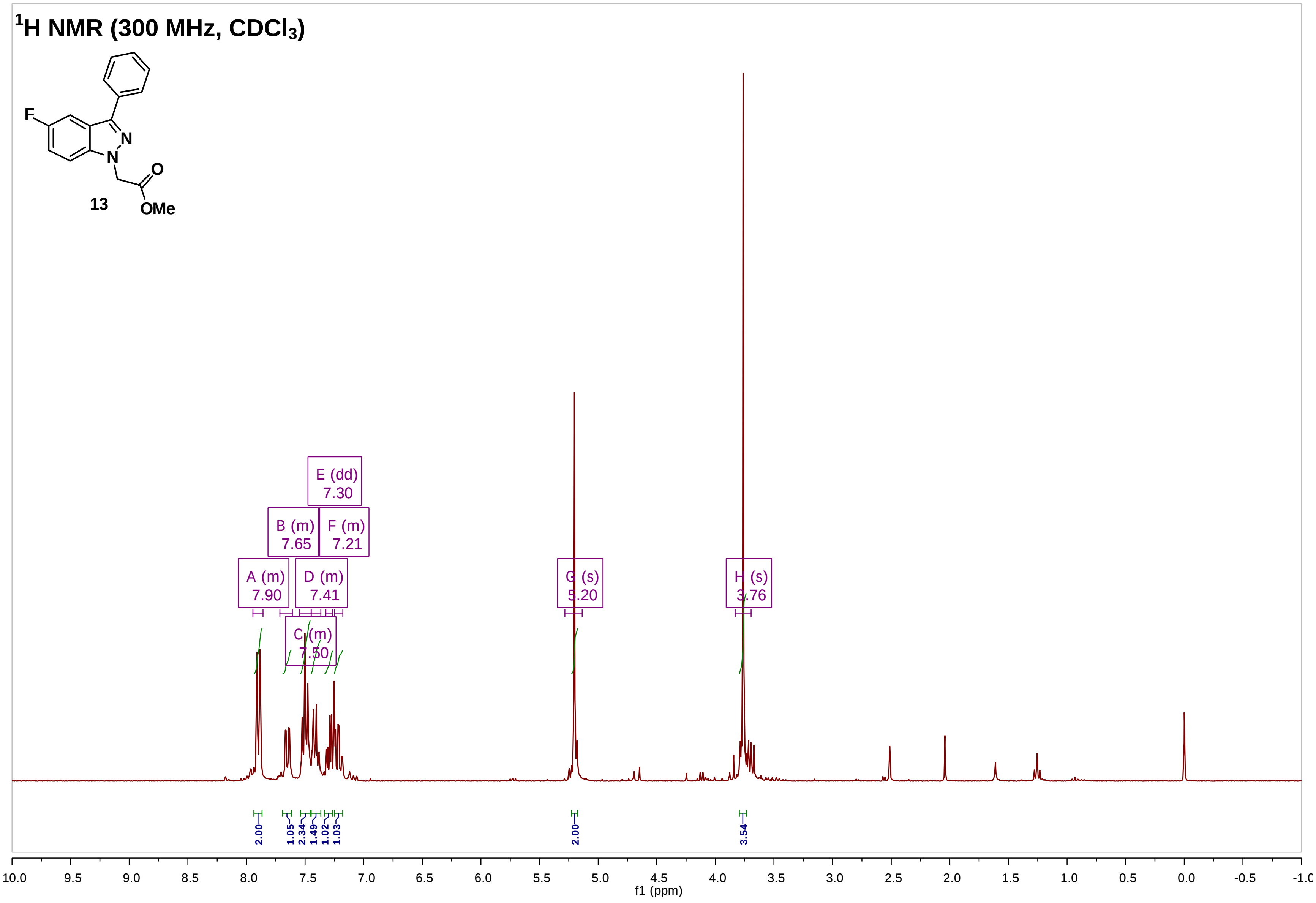
1H NMR (300 MHz, CDCl3)
F
N
N
O
13
OMe
E (dd)
7.30
B (m)
7.65
F (m)
7.21
A (m)
7.90
D (m)
7.41
C (m)
7.50
G (s)
5.20
H (s)
3.76
2.00
1.05
2.34
1.49
1.02
1.03
2.00
3.54
10.0
9.5
9.0
8.5
8.0
7.5
7.0
6.5
6.0
5.5
5.0
4.5
4.0
3.5
3.0
2.5
2.0
1.5
1.0
0.5
0.0
-0.5
f1 (ppm)

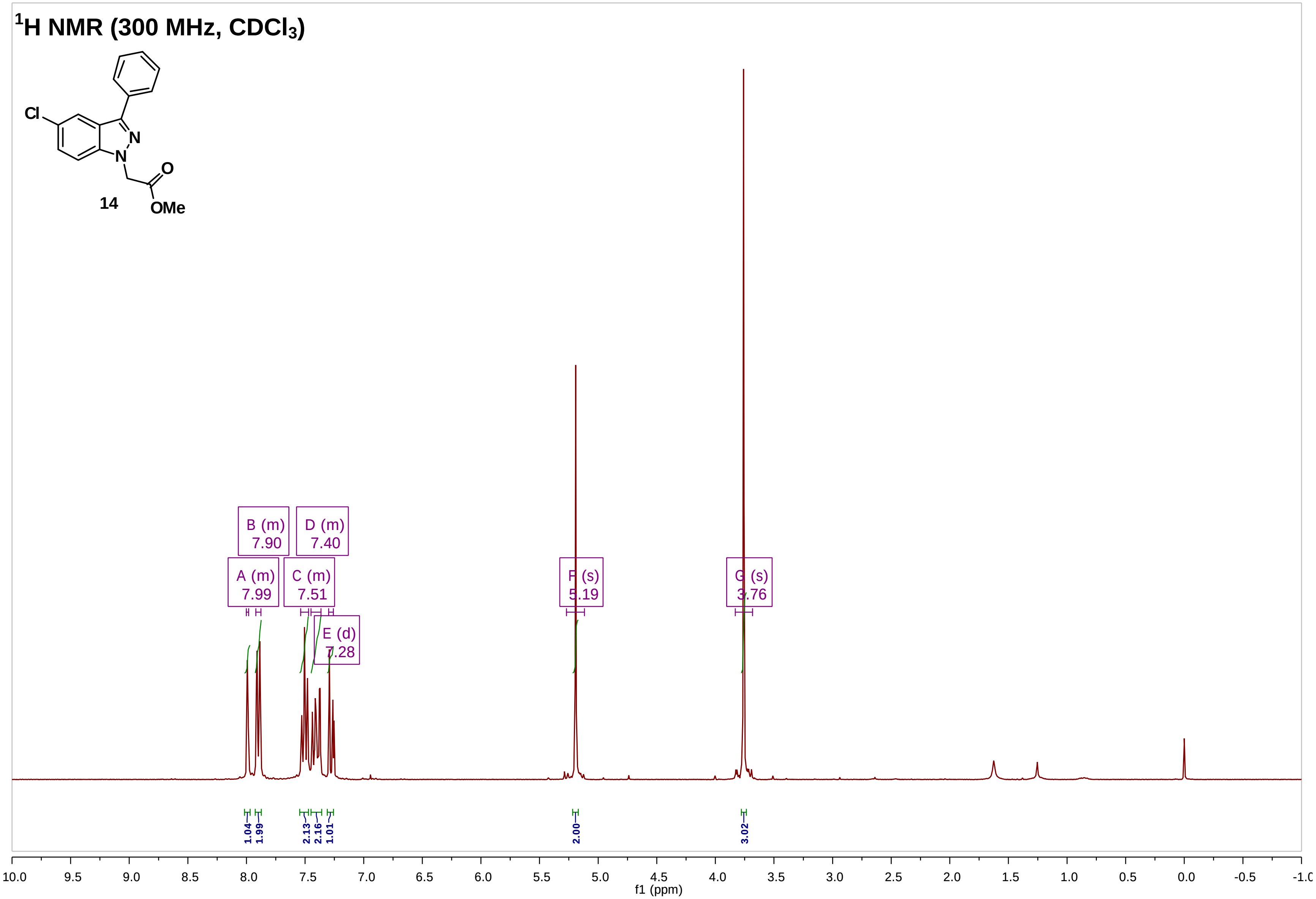

1H NMR (300 MHz, CDCl3)
Cl
N
N
O
OMe
14
A (m) 7.99
B (m) 7.90
C (m) 7.51
D (m) 7.40
E (d) 7.28
F (s) 5.19
G (s) 3.76
1.04
1.99
2.13
2.16
1.01
2.00
3.02
10.0 9.5 9.0 8.5 8.0 7.5 7.0 6.5 6.0 5.5 5.0 4.5 4.0 3.5 3.0 2.5 2.0 1.5 1.0 0.5 0.0 -0.5 -1.0
f1 (ppm)

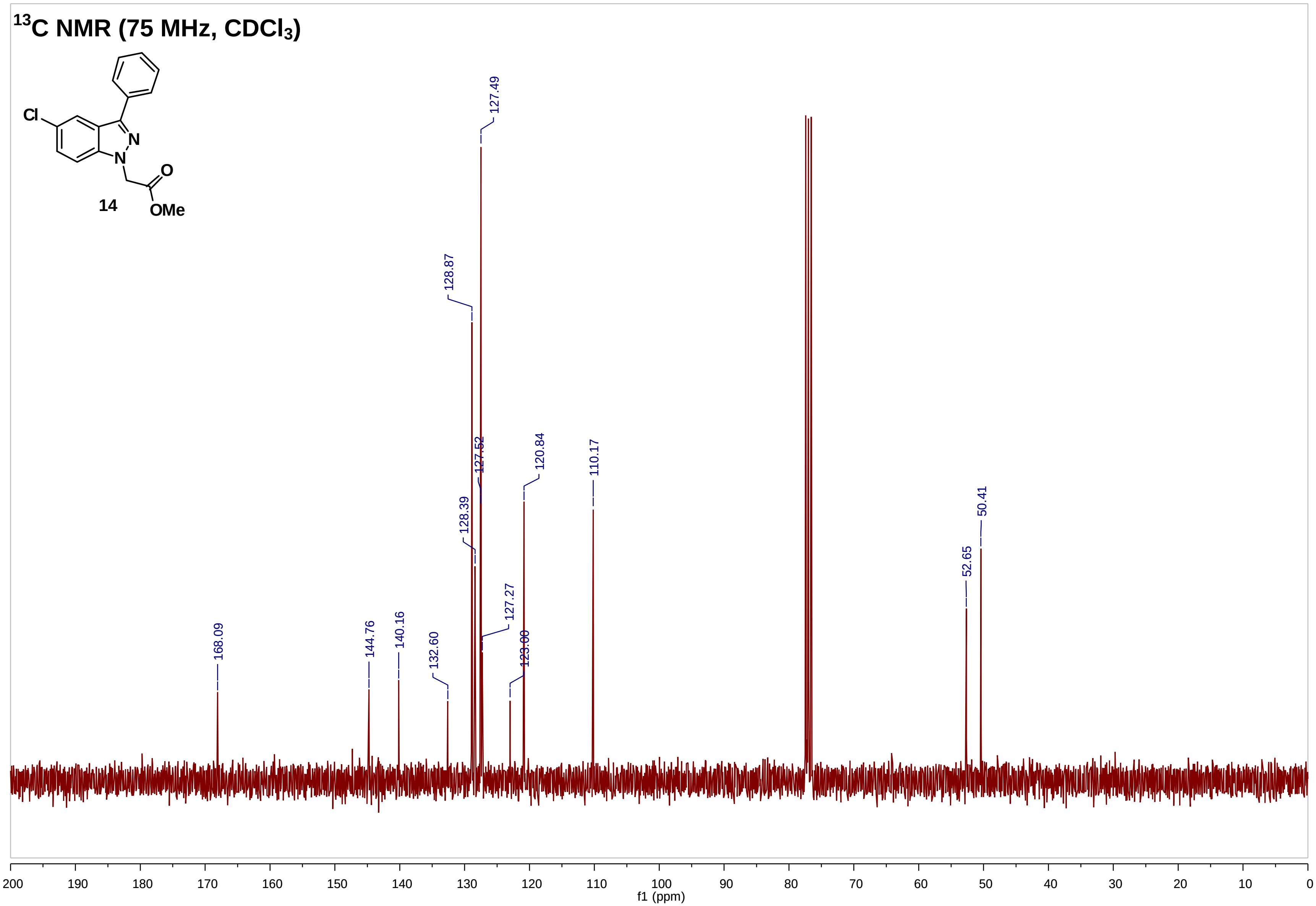

^{13}C NMR (75 MHz, CDCl$_3$)
Cl
N
N
O
OMe
14
168.09
144.76
140.16
132.60
128.87
128.39
127.52
127.49
127.27
123.00
120.84
110.17
52.65
50.41
200
190
180
170
160
150
140
130
120
110
100
90
80
70
60
50
40
30
20
10
0
f1 (ppm)

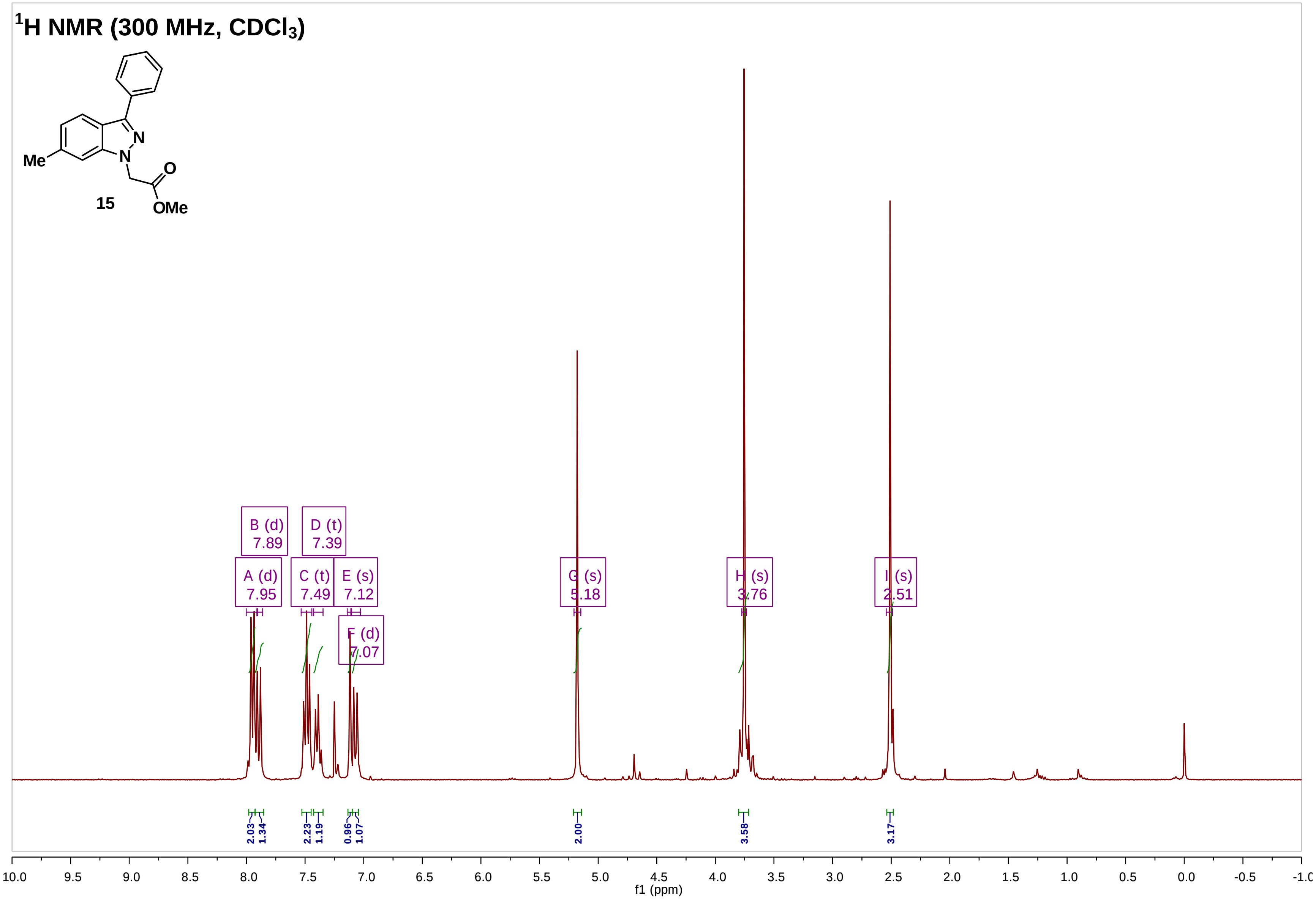

^{1}H NMR (300 MHz, CDCl$_3$)
Me
N
N
O
OMe
15
A (d) 7.95
B (d) 7.89
C (t) 7.49
D (t) 7.39
E (s) 7.12
F (d) 7.07
G (s) 5.18
H (s) 3.76
I (s) 2.51
2.03
1.34
2.23
1.19
0.96
1.07
2.00
3.58
3.17
10.0 9.5 9.0 8.5 8.0 7.5 7.0 6.5 6.0 5.5 5.0 4.5 4.0 3.5 3.0 2.5 2.0 1.5 1.0 0.5 0.0 -0.5 -1.0
f1 (ppm)

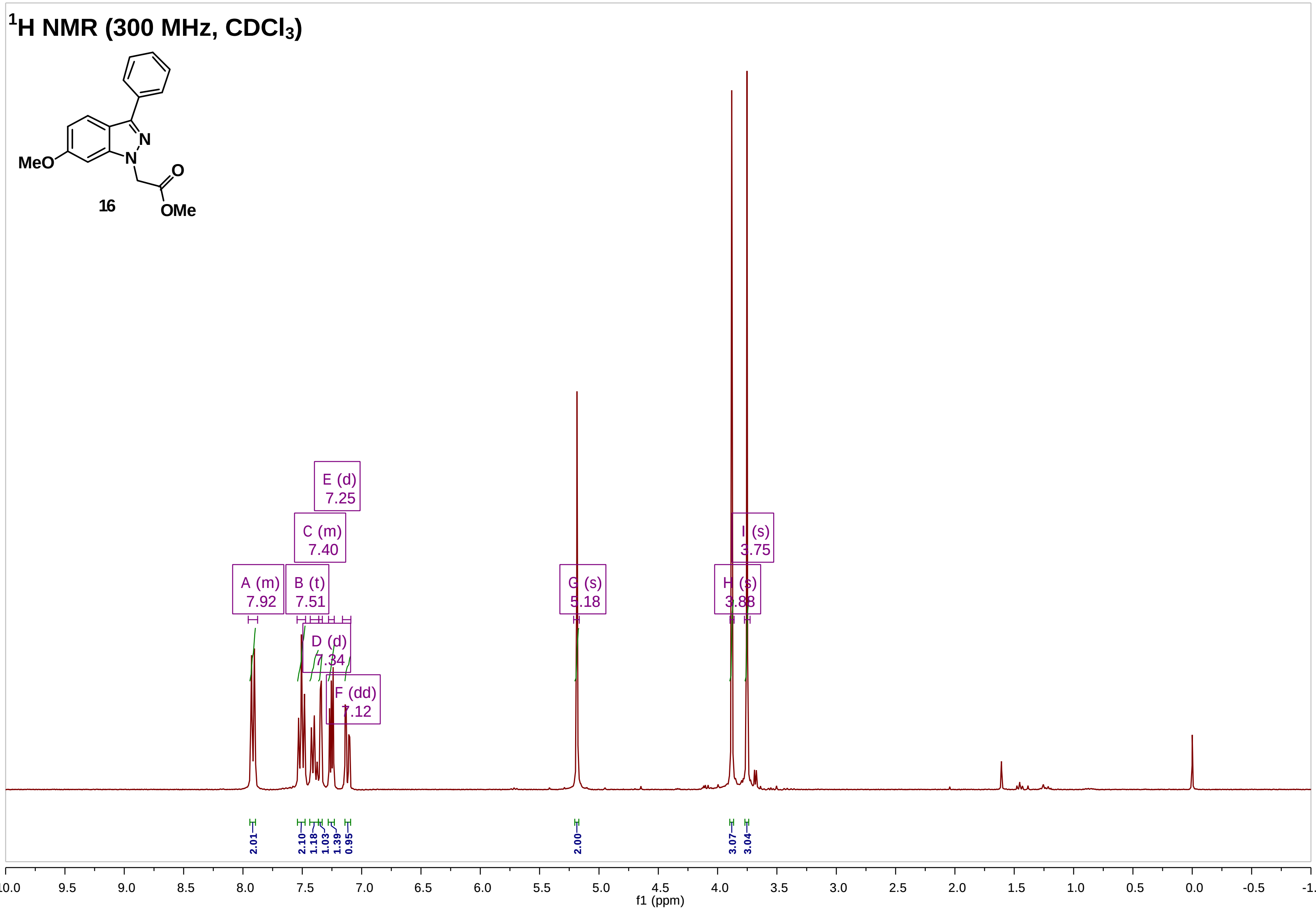

1H NMR (300 MHz, CDCl3)
MeO
N
N
O
OMe
16
A (m)
7.92
B (t)
7.51
C (m)
7.40
D (d)
7.34
E (d)
7.25
F (dd)
7.12
G (s)
5.18
H (s)
3.88
I (s)
3.75
2.01
2.10
1.18
1.03
1.39
0.95
2.00
3.07
3.04
10.0
9.5
9.0
8.5
8.0
7.5
7.0
6.5
6.0
5.5
5.0
4.5
4.0
3.5
3.0
2.5
2.0
1.5
1.0
0.5
0.0
-0.5
f1 (ppm)

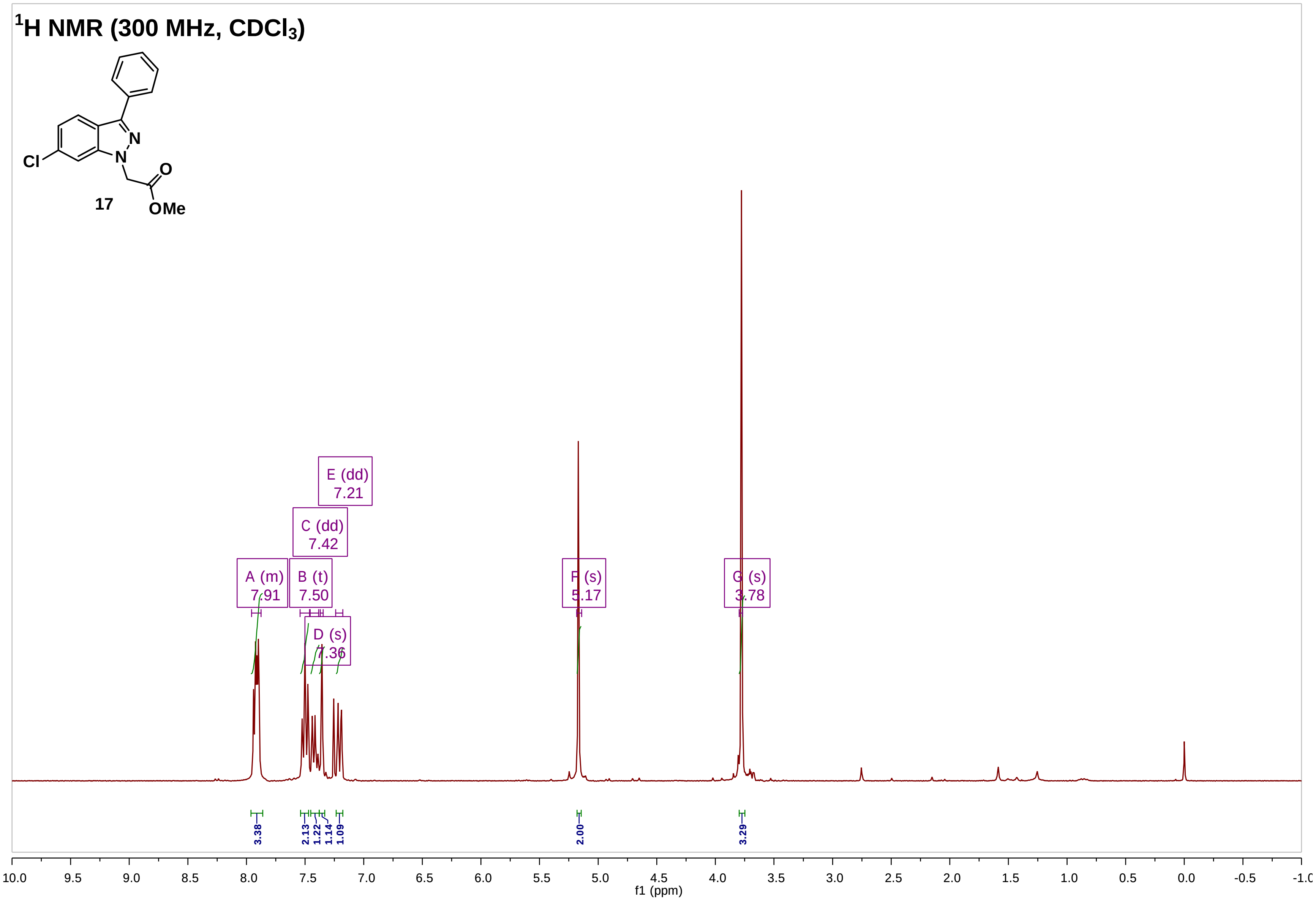

1H NMR (300 MHz, CDCl3)
Cl
N
N
O
OMe
17
A (m) 7.91
B (t) 7.50
C (dd) 7.42
D (s) 7.36
E (dd) 7.21
F (s) 5.17
G (s) 3.78
3.38
2.13
1.22
1.14
1.09
2.00
3.29
f1 (ppm)

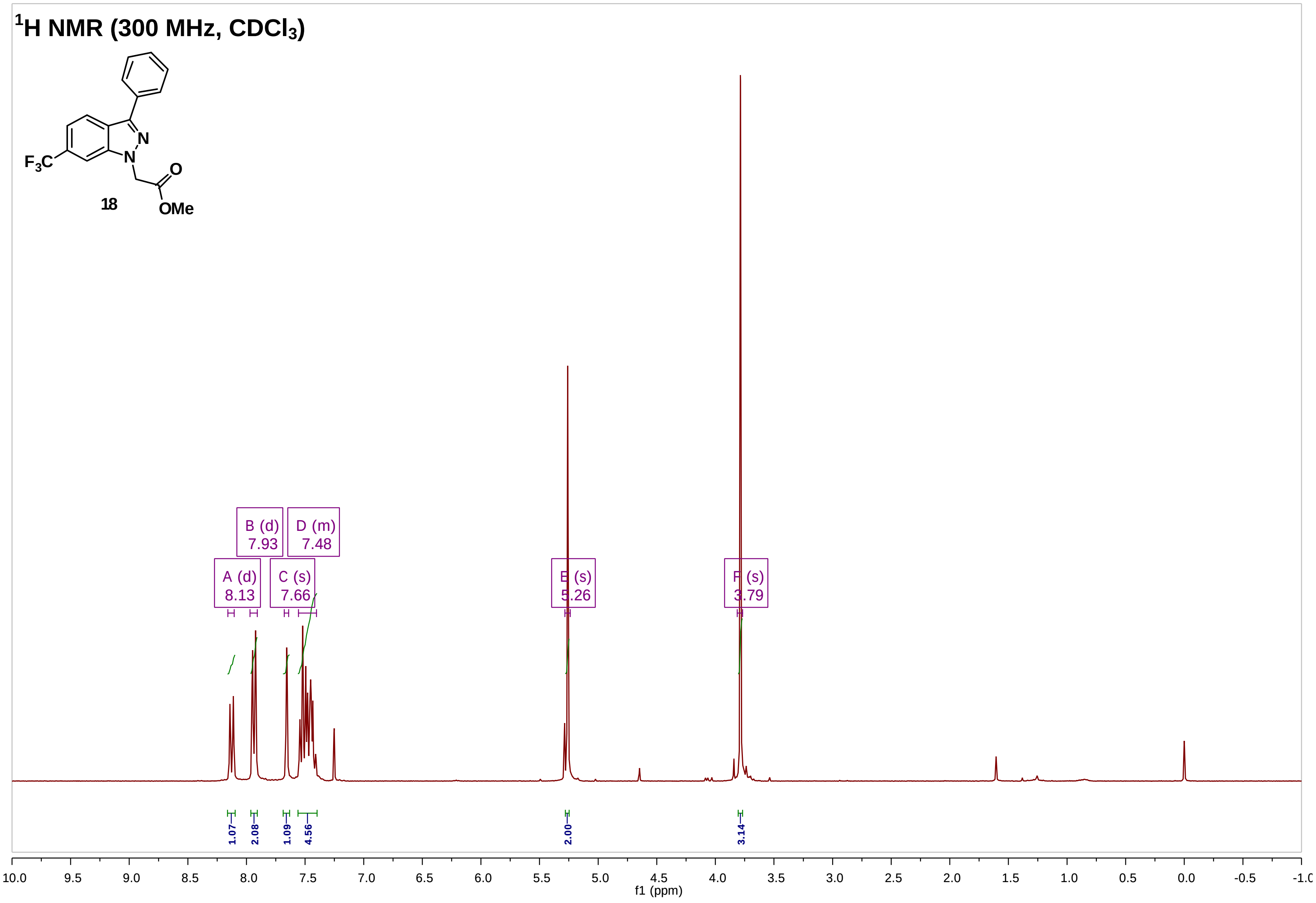

^{1}H NMR (300 MHz, $CDCl_3$)
F_3C
N
N
O
OMe
18
A (d) 8.13
B (d) 7.93
C (s) 7.66
D (m) 7.48
E (s) 5.26
F (s) 3.79
1.07
2.08
1.09
4.56
2.00
3.14
10.0 9.5 9.0 8.5 8.0 7.5 7.0 6.5 6.0 5.5 5.0 4.5 4.0 3.5 3.0 2.5 2.0 1.5 1.0 0.5 0.0 -0.5
f1 (ppm)

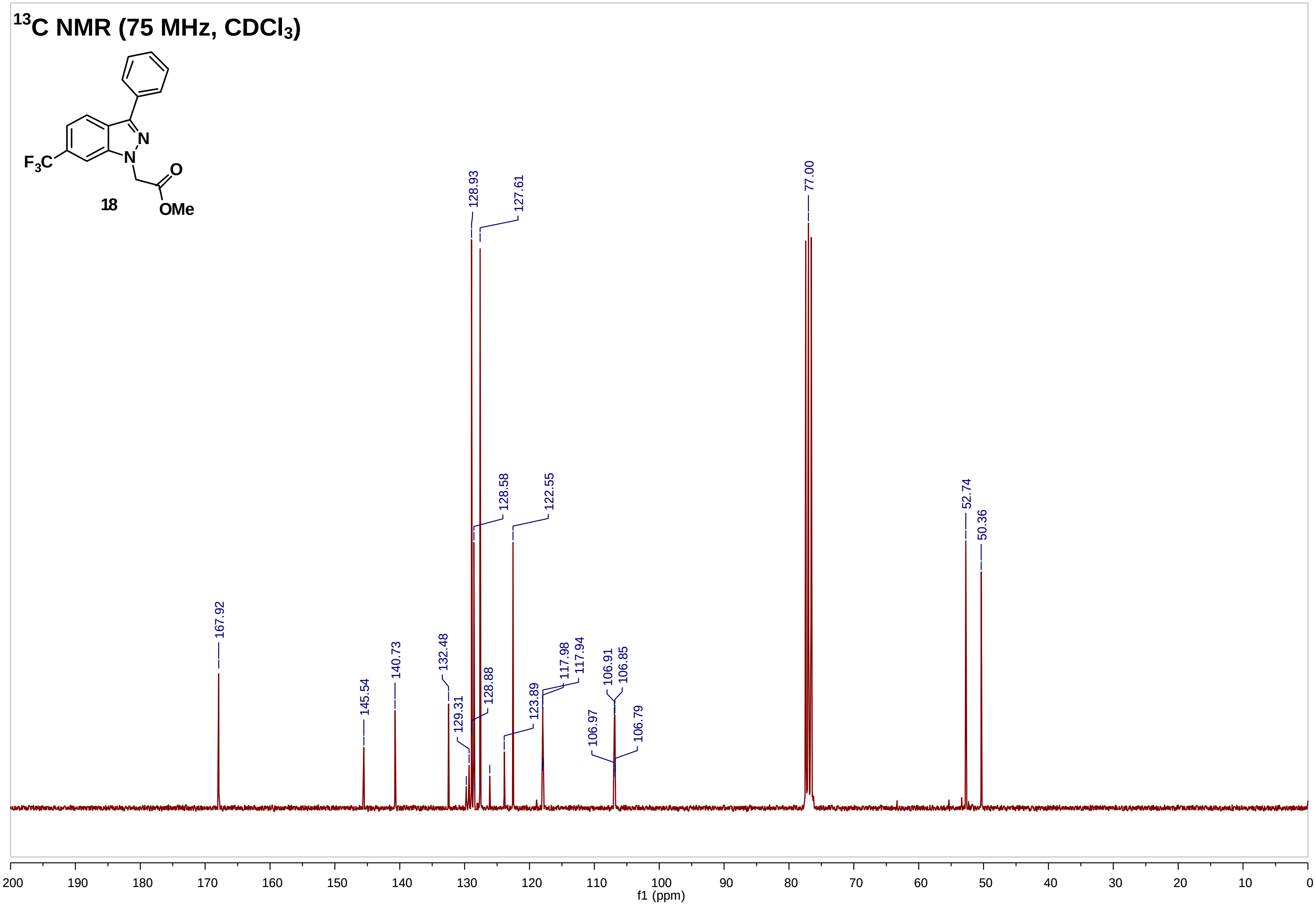

^{13}C NMR (75 MHz, $CDCl_3$)
F3C
N
N
O
OMe
18
167.92
145.54
140.73
132.48
129.31
128.93
128.88
128.58
127.61
123.89
122.55
117.98
117.94
106.97
106.91
106.85
106.79
77.00
52.74
50.36
200
190
180
170
160
150
140
130
120
110
100
90
80
70
60
50
40
30
20
10
0
f1 (ppm)

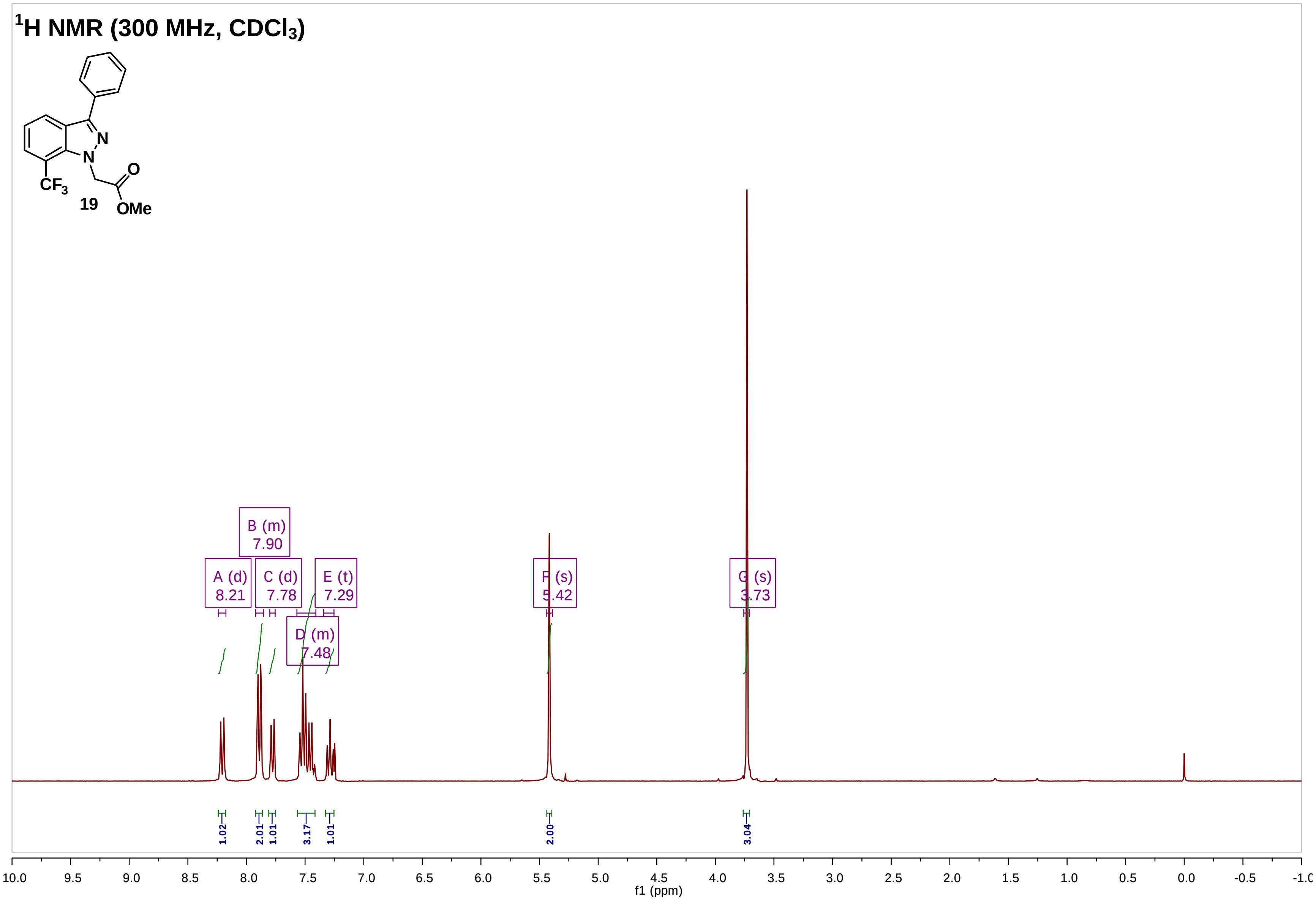

^{1}H NMR (300 MHz, $CDCl_3$)
19
N
N
O
OMe
CF_3
A (d) 8.21
B (m) 7.90
C (d) 7.78
D (m) 7.48
E (t) 7.29
F (s) 5.42
G (s) 3.73
1.02
2.01
1.01
3.17
1.01
2.00
3.04
f1 (ppm)

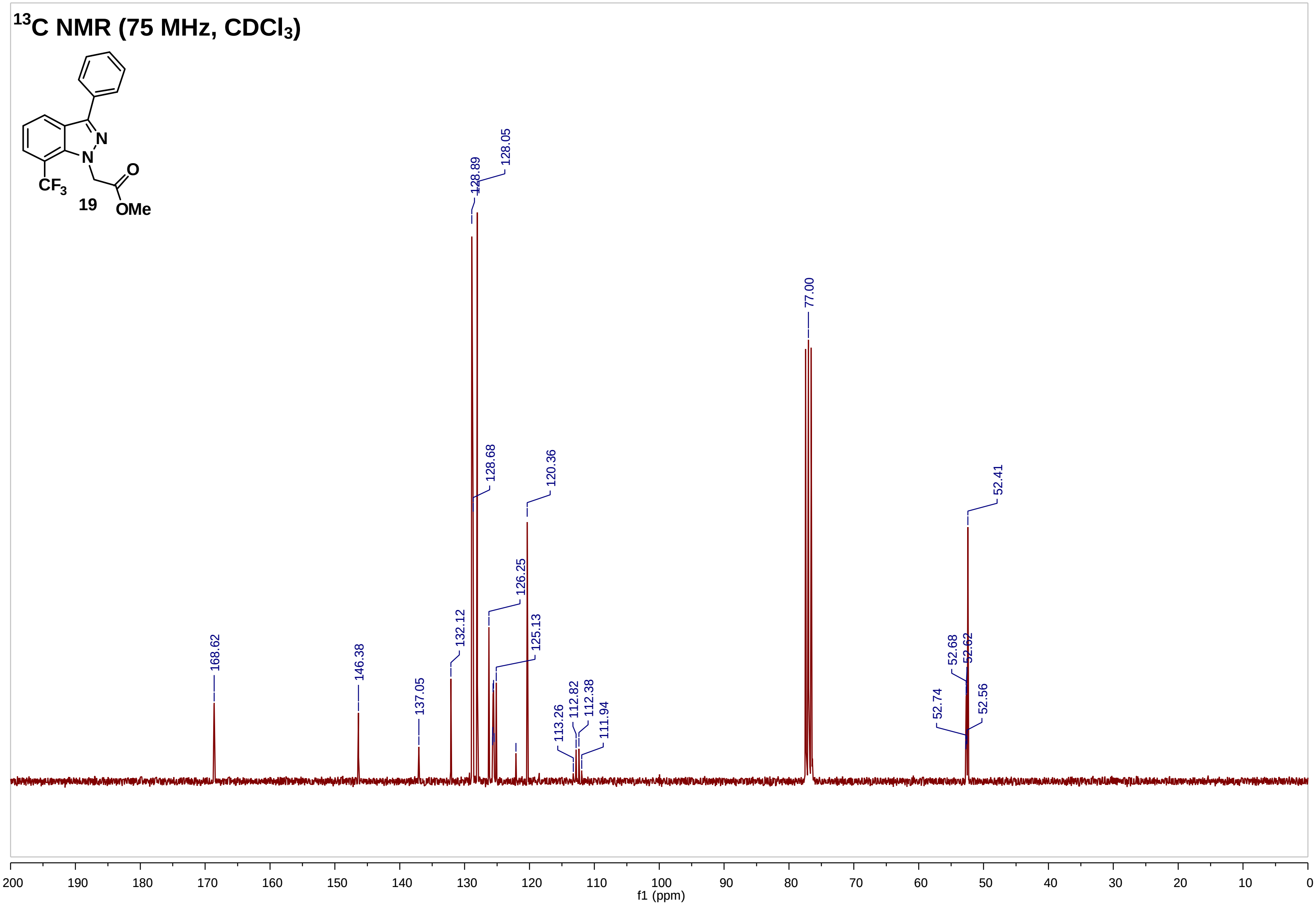

^{13}C NMR (75 MHz, $CDCl_3$)
N
N
O
CF_3
19
OMe
168.62
146.38
137.05
132.12
128.89
128.05
128.68
126.25
125.13
120.36
113.26
112.82
112.38
111.94
77.00
52.74
52.68
52.62
52.56
52.41
200
190
180
170
160
150
140
130
120
110
100
90
80
70
60
50
40
30
20
10
0
f1 (ppm)

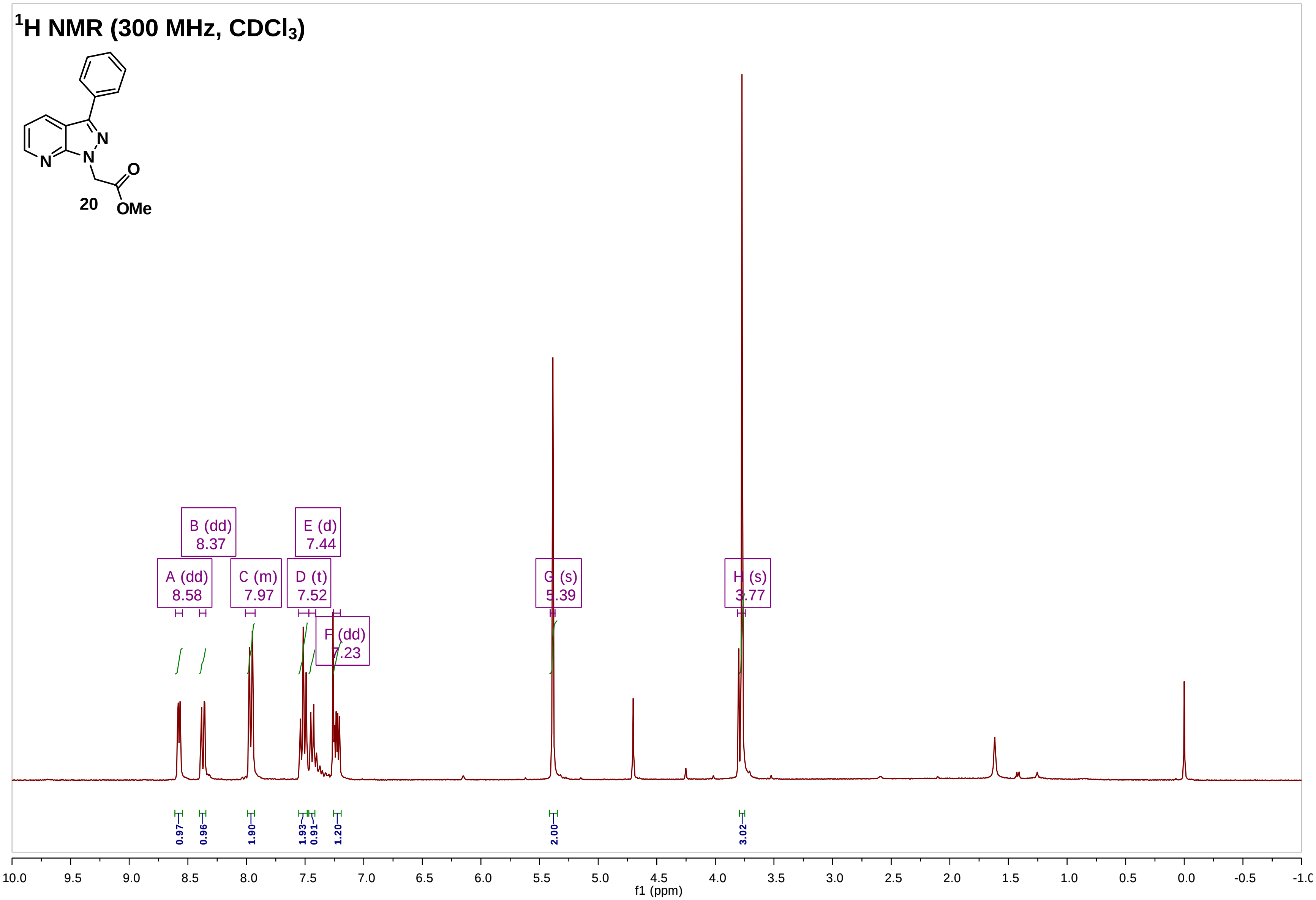

1H NMR (300 MHz, CDCl3)
N
N
N
O
OMe
20
A (dd) 8.58
B (dd) 8.37
C (m) 7.97
D (t) 7.52
E (d) 7.44
F (dd) 7.23
G (s) 5.39
H (s) 3.77
0.97
0.96
1.90
1.93
0.91
1.20
2.00
3.02
10.0 9.5 9.0 8.5 8.0 7.5 7.0 6.5 6.0 5.5 5.0 4.5 4.0 3.5 3.0 2.5 2.0 1.5 1.0 0.5 0.0 -0.5
f1 (ppm)

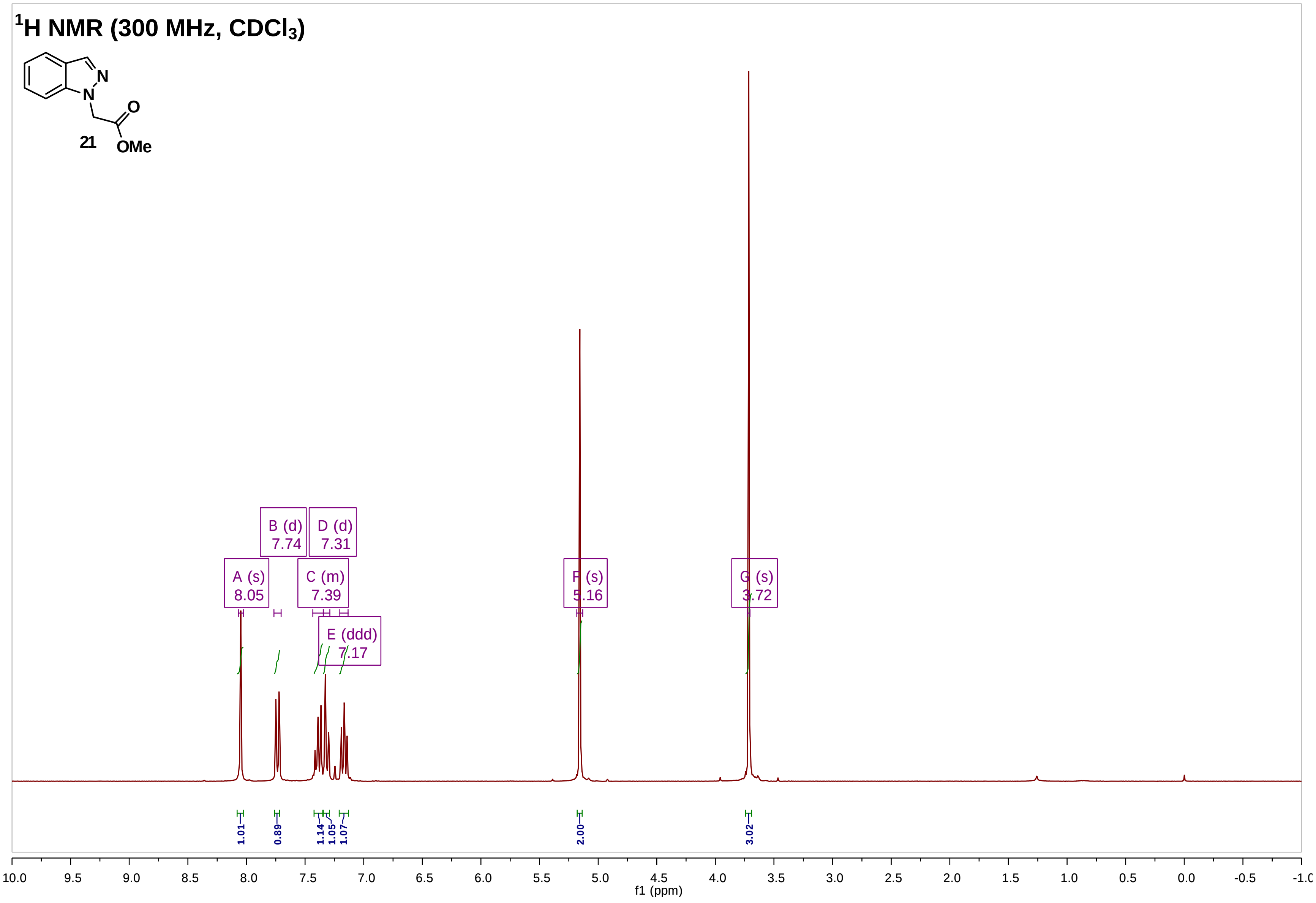

^{1}H NMR (300 MHz, CDCl$_3$)
N
N
O
OMe
21
A (s)
8.05
B (d)
7.74
C (m)
7.39
D (d)
7.31
E (ddd)
7.17
F (s)
5.16
G (s)
3.72
1.01
0.89
1.14
1.05
1.07
2.00
3.02
10.0 9.5 9.0 8.5 8.0 7.5 7.0 6.5 6.0 5.5 5.0 4.5 4.0 3.5 3.0 2.5 2.0 1.5 1.0 0.5 0.0 -0.5
f1 (ppm)

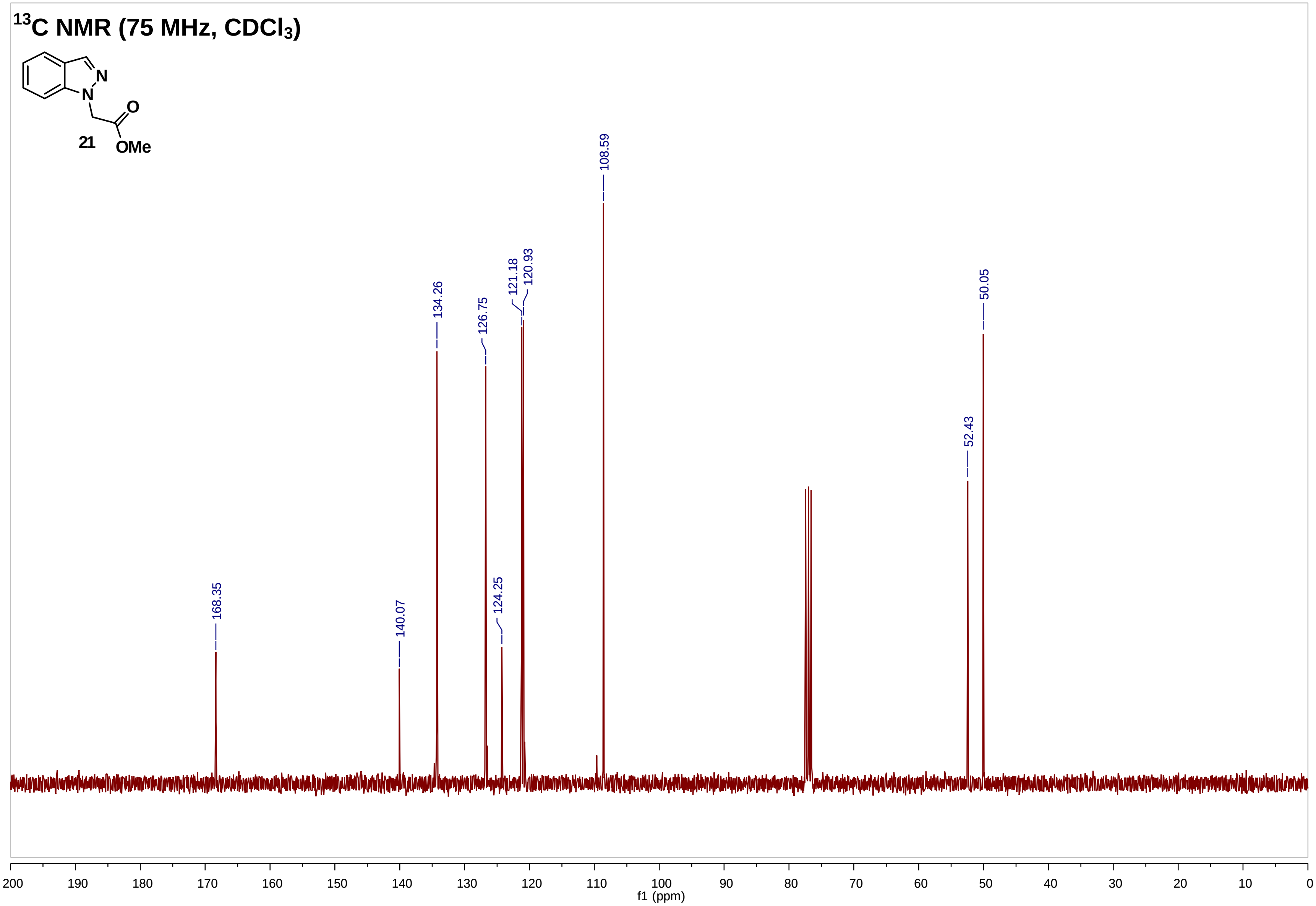

13C NMR (75 MHz, CDCl3)
N
N
O
21
OMe
168.35
140.07
134.26
126.75
124.25
121.18
120.93
108.59
52.43
50.05
200
190
180
170
160
150
140
130
120
110
100
f1 (ppm)
90
80
70
60
50
40
30
20
10
0

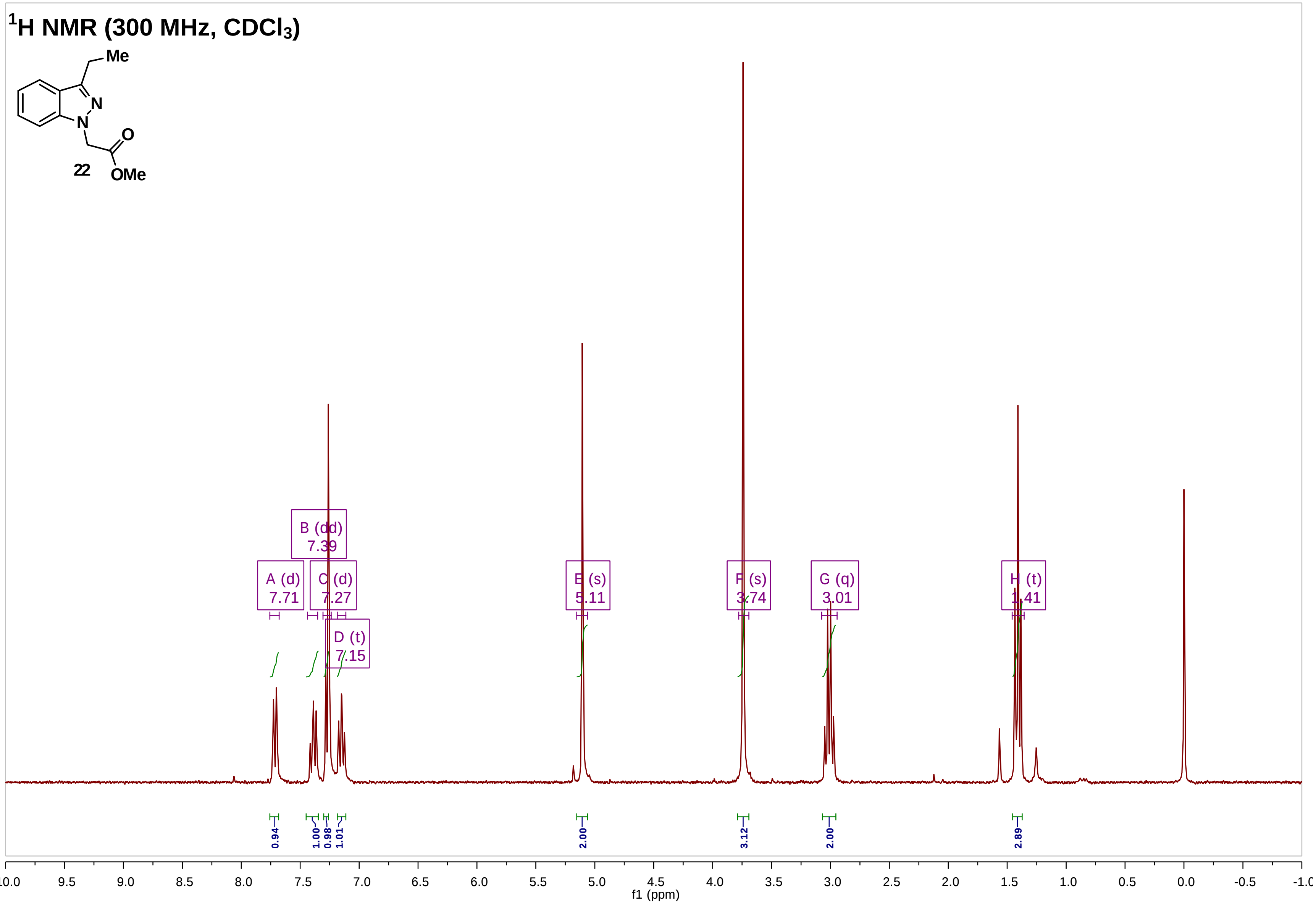

^{1}H NMR (300 MHz, $CDCl_3$)
Me
N
N
O
OMe
22
A (d) 7.71
B (dd) 7.39
C (d) 7.27
D (t) 7.15
E (s) 5.11
F (s) 3.74
G (q) 3.01
H (t) 1.41
0.94
1.00
0.98
1.01
2.00
3.12
2.00
2.89
10.0 9.5 9.0 8.5 8.0 7.5 7.0 6.5 6.0 5.5 5.0 4.5 4.0 3.5 3.0 2.5 2.0 1.5 1.0 0.5 0.0 -0.5 -1.0
f1 (ppm)

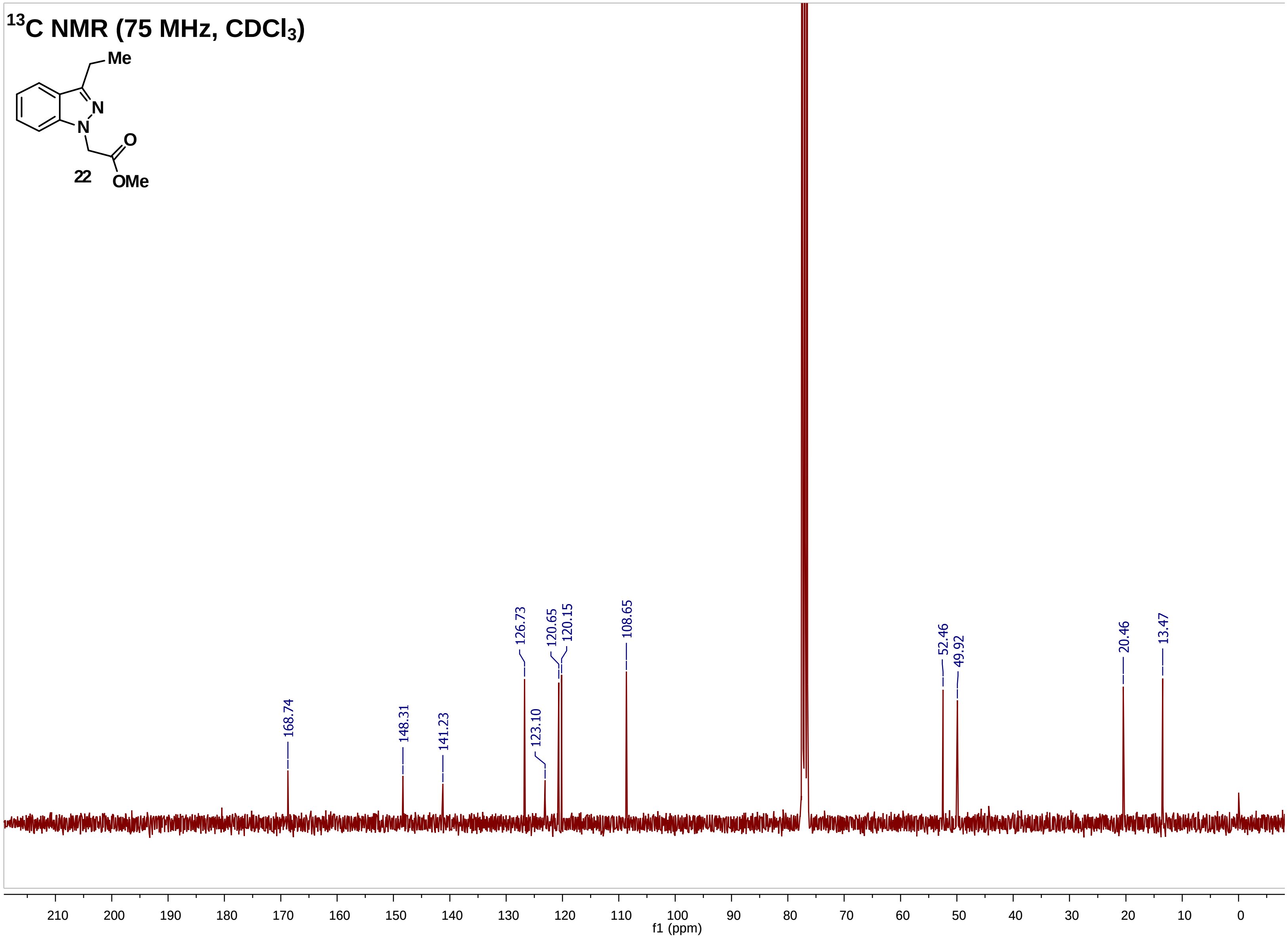

^{13}C NMR (75 MHz, CDCl$_3$)
Me
N
N
O
22
OMe
168.74
148.31
141.23
126.73
123.10
120.65
120.15
108.65
52.46
49.92
20.46
13.47
210
200
190
180
170
160
150
140
130
120
110
100
90
80
70
60
50
40
30
20
10
0
f1 (ppm)

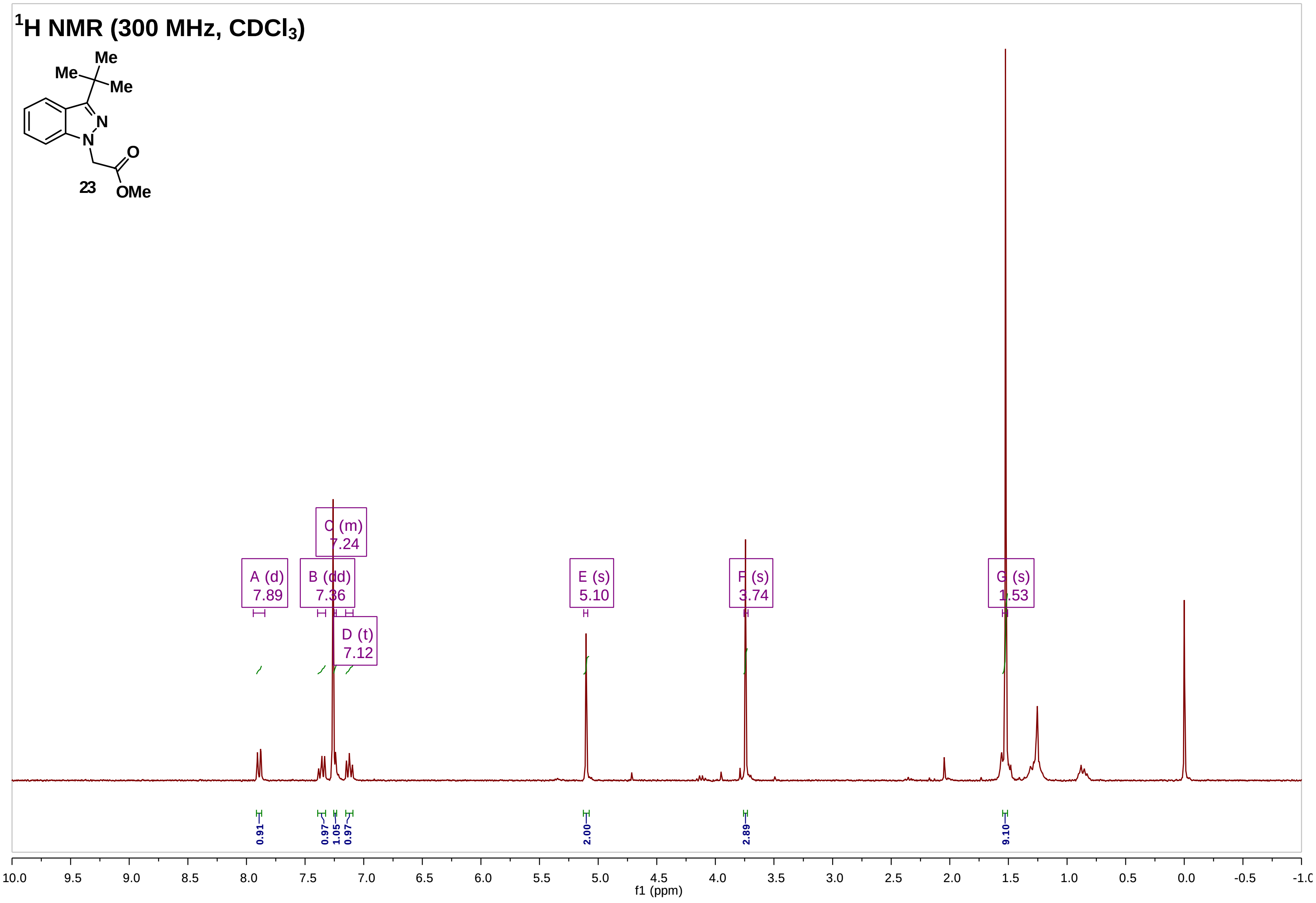

^{1}H NMR (300 MHz, $CDCl_3$)
Me
Me
Me
N
N
O
OMe
23
A (d) 7.89
B (dd) 7.36
C (m) 7.24
D (t) 7.12
E (s) 5.10
F (s) 3.74
G (s) 1.53
0.91
0.97
1.05
0.97
2.00
2.89
9.10
10.0 9.5 9.0 8.5 8.0 7.5 7.0 6.5 6.0 5.5 5.0 4.5 4.0 3.5 3.0 2.5 2.0 1.5 1.0 0.5 0.0 -0.5 -1.0
f1 (ppm)

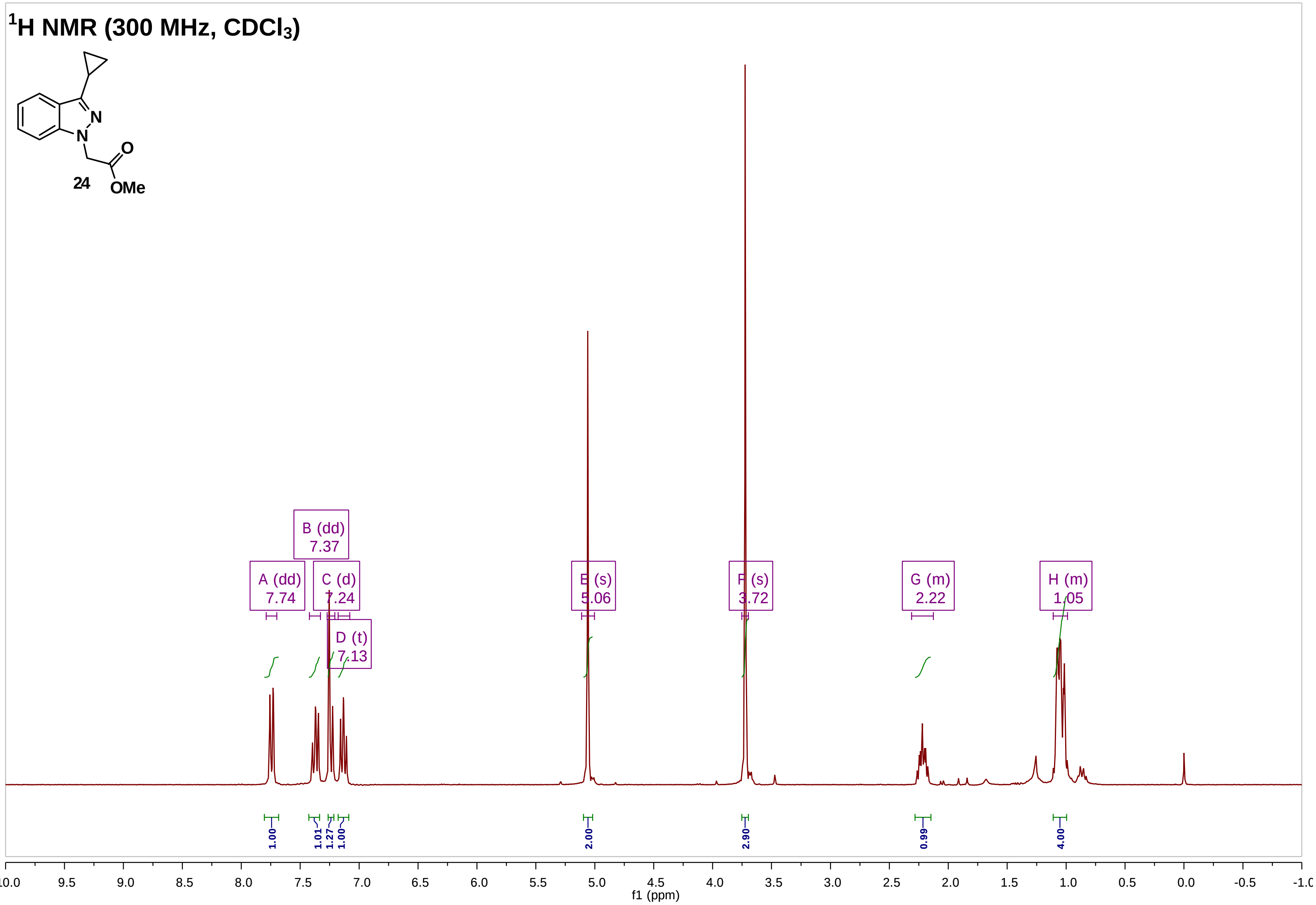

1H NMR (300 MHz, CDCl3)
24
N
N
O
OMe
A (dd) 7.74
B (dd) 7.37
C (d) 7.24
D (t) 7.13
E (s) 5.06
F (s) 3.72
G (m) 2.22
H (m) 1.05
1.00
1.01
1.27
1.00
2.00
2.90
0.99
4.00
f1 (ppm)

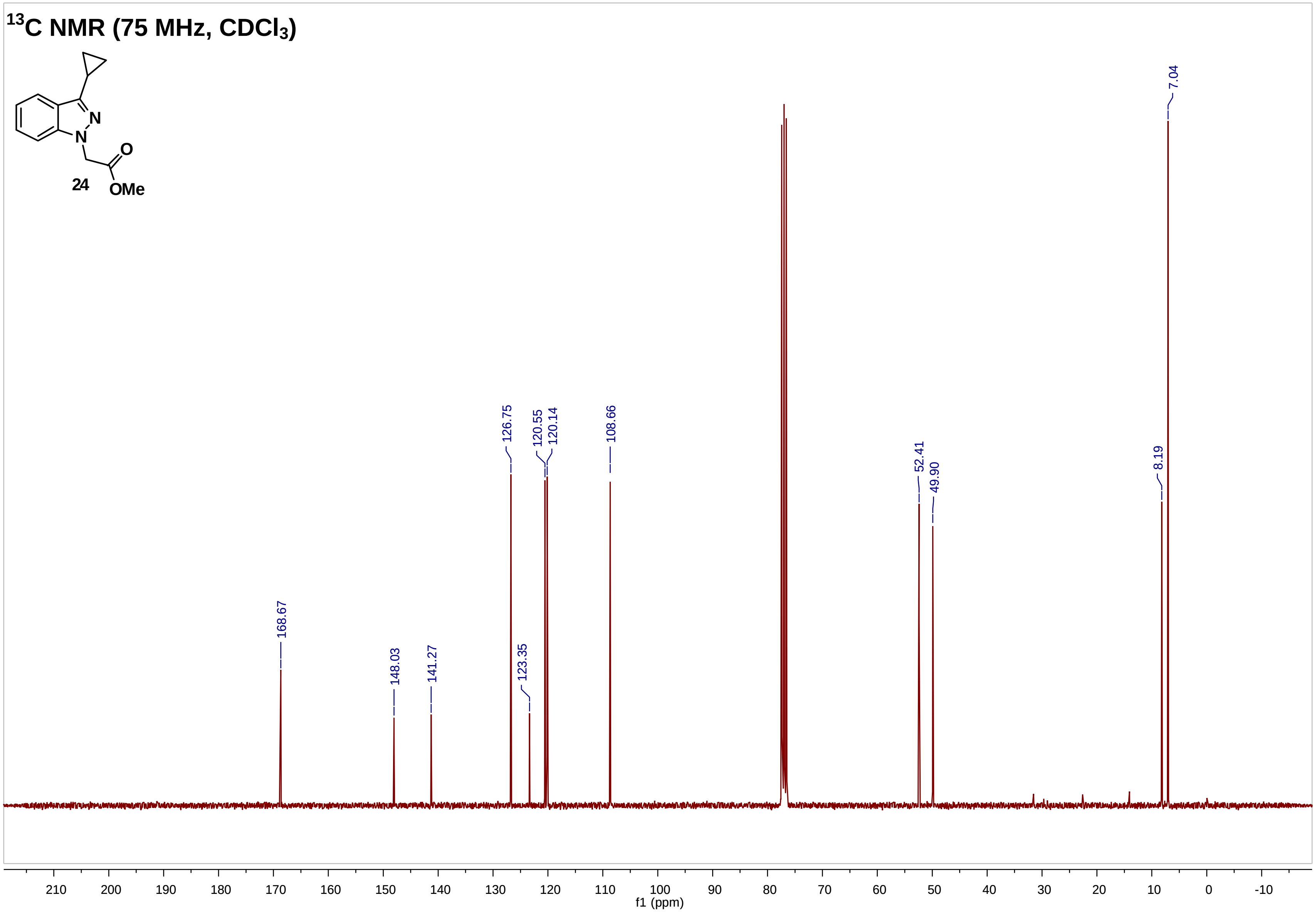

13C NMR (75 MHz, CDCl3)
N
N
O
OMe
24
168.67
148.03
141.27
126.75
123.35
120.55
120.14
108.66
52.41
49.90
8.19
7.04
210
200
190
180
170
160
150
140
130
120
110
100
90
80
70
60
50
40
30
20
10
0
-10
f1 (ppm)

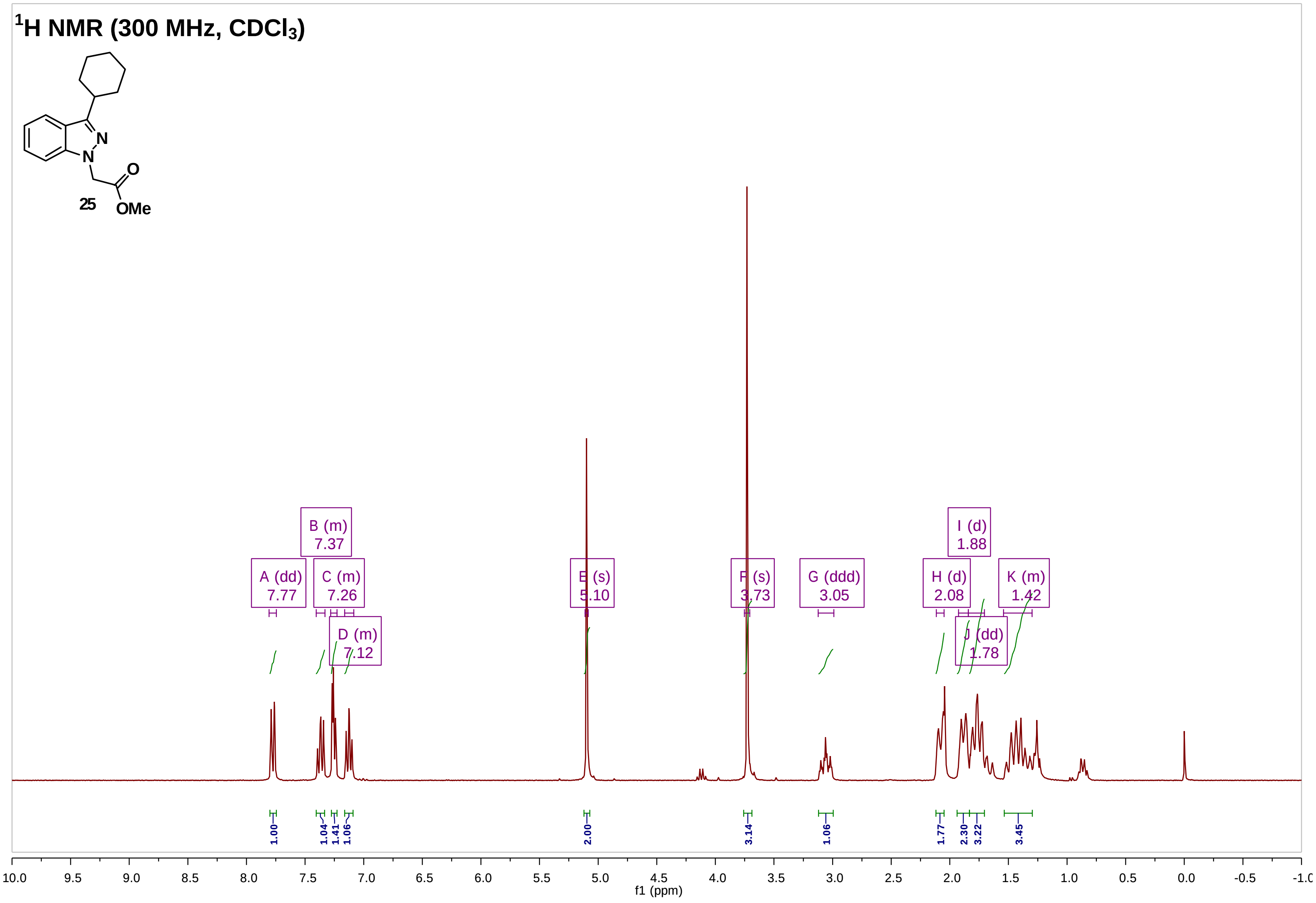
1H NMR (300 MHz, CDCl3)
N
N
O
OMe
25
A (dd) 7.77
B (m) 7.37
C (m) 7.26
D (m) 7.12
E (s) 5.10
F (s) 3.73
G (ddd) 3.05
H (d) 2.08
I (d) 1.88
J (dd) 1.78
K (m) 1.42
1.00
1.04
1.41
1.06
2.00
3.14
1.06
1.77
2.30
3.22
3.45
10.0 9.5 9.0 8.5 8.0 7.5 7.0 6.5 6.0 5.5 5.0 4.5 4.0 3.5 3.0 2.5 2.0 1.5 1.0 0.5 0.0 -0.5
f1 (ppm)

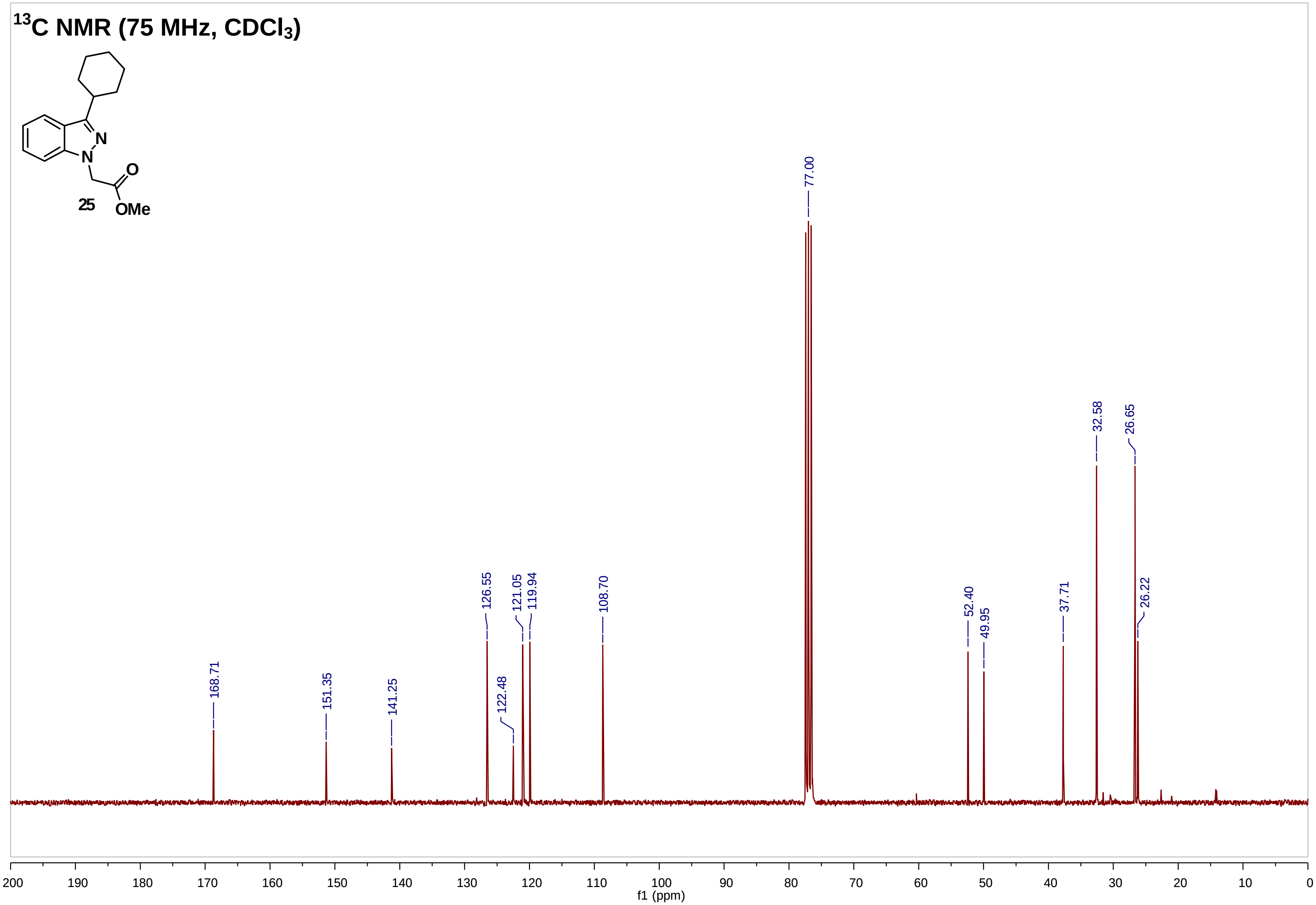

13C NMR (75 MHz, CDCl3)
N
N
O
25
OMe
168.71
151.35
141.25
126.55
122.48
121.05
119.94
108.70
77.00
52.40
49.95
37.71
32.58
26.65
26.22
200
190
180
170
160
150
140
130
120
110
100
90
80
70
60
50
40
30
20
10
0
f1 (ppm)

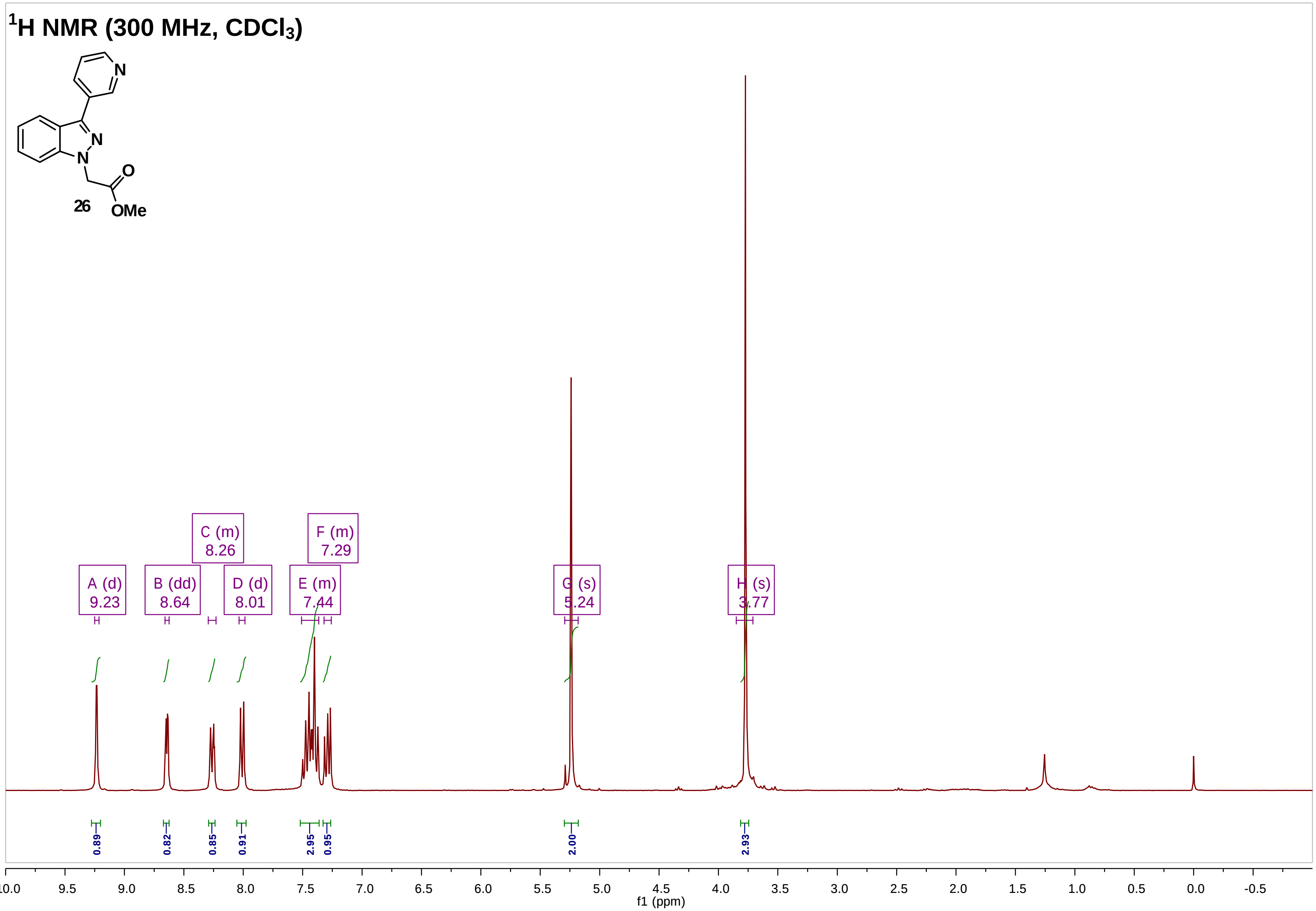

1H NMR (300 MHz, CDCl3)
26
OMe
A (d) 9.23
B (dd) 8.64
C (m) 8.26
D (d) 8.01
E (m) 7.44
F (m) 7.29
G (s) 5.24
H (s) 3.77
0.89
0.82
0.85
0.91
2.95
0.95
2.00
2.93
f1 (ppm)

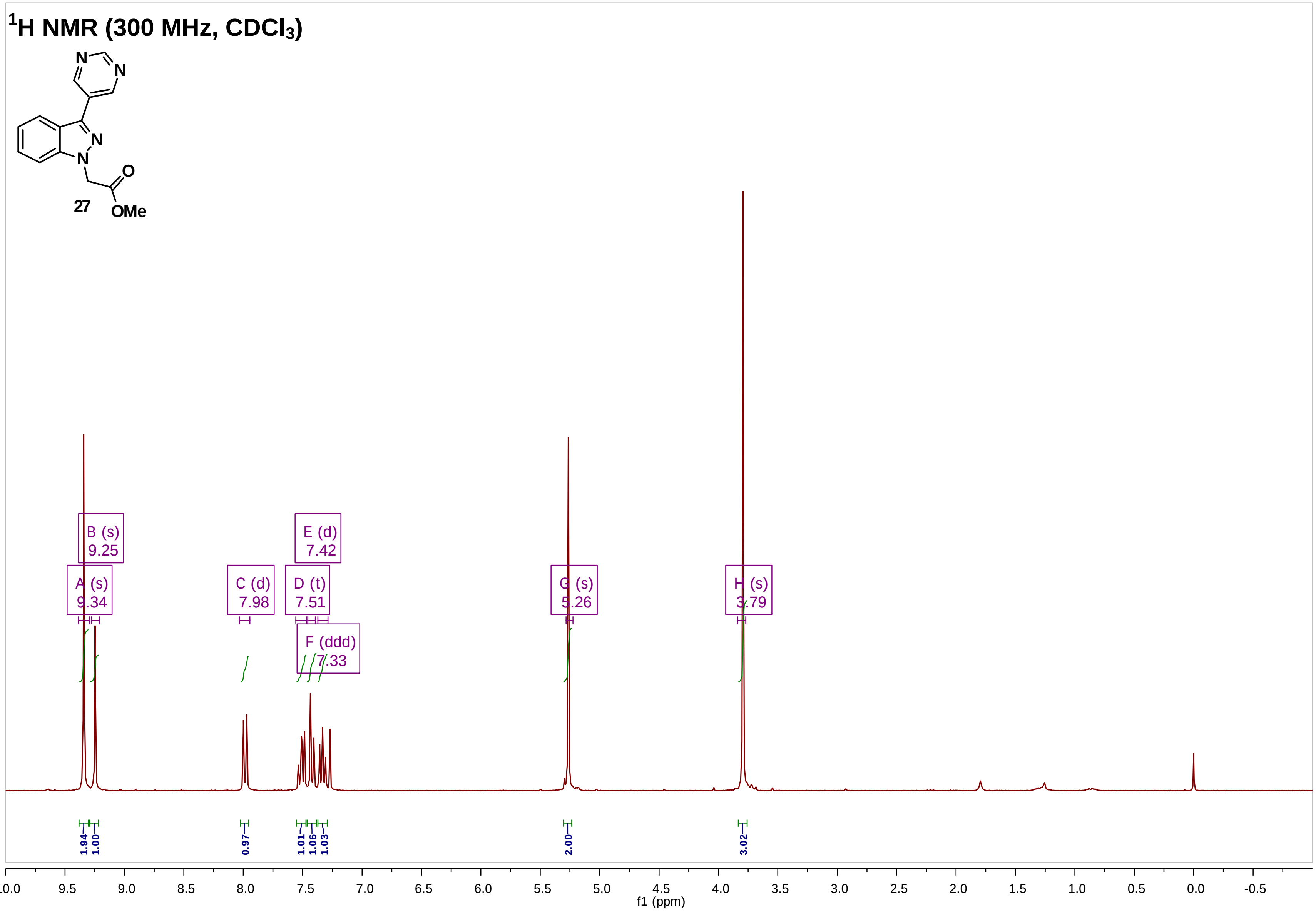

1H NMR (300 MHz, CDCl3)
27
A (s) 9.34
B (s) 9.25
C (d) 7.98
D (t) 7.51
E (d) 7.42
F (ddd) 7.33
G (s) 5.26
H (s) 3.79
1.94
1.00
0.97
1.01
1.06
1.03
2.00
3.02
f1 (ppm)

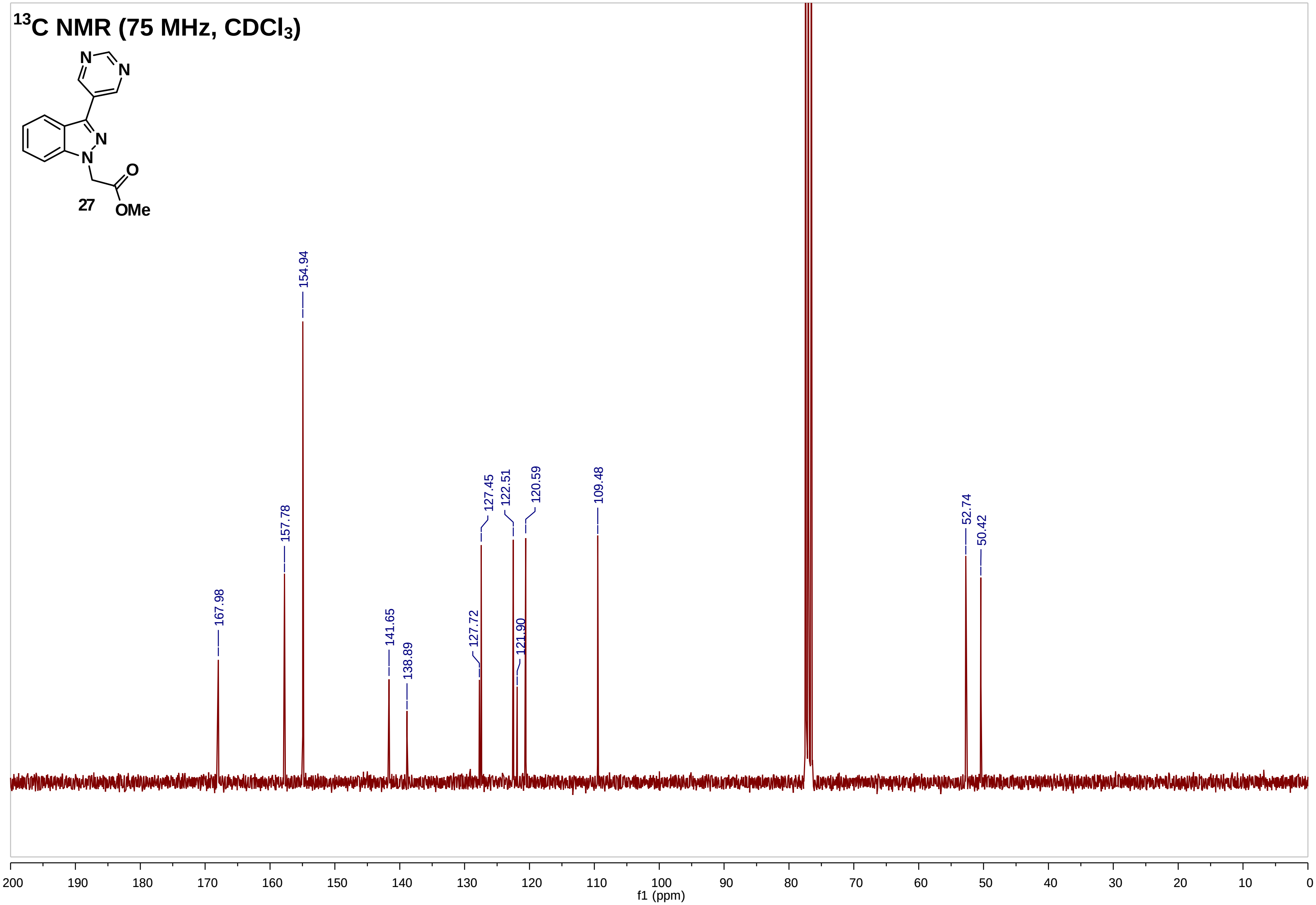

^{13}C NMR (75 MHz, $CDCl_3$)
N
N
N
N
O
OMe
27
167.98
157.78
154.94
141.65
138.89
127.72
127.45
122.51
121.90
120.59
109.48
52.74
50.42
200
190
180
170
160
150
140
130
120
110
100
90
80
70
60
50
40
30
20
10
0
f1 (ppm)

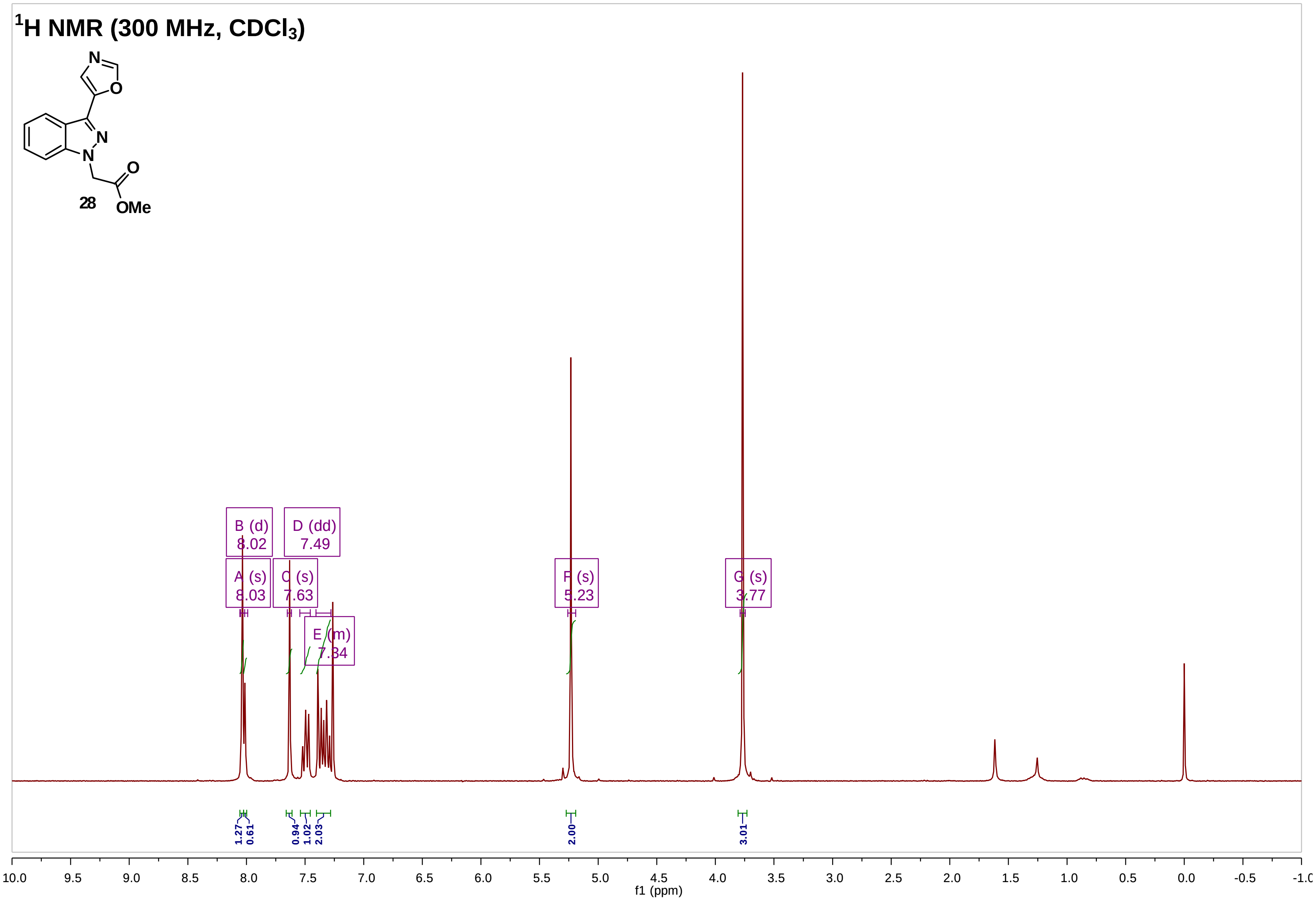

^{1}H NMR (300 MHz, $CDCl_3$)
28
B (d) 8.02
D (dd) 7.49
A (s) 8.03
C (s) 7.63
E (m) 7.34
F (s) 5.23
G (s) 3.77
f1 (ppm)

^{1}H NMR (300 MHz, $CDCl_3$)

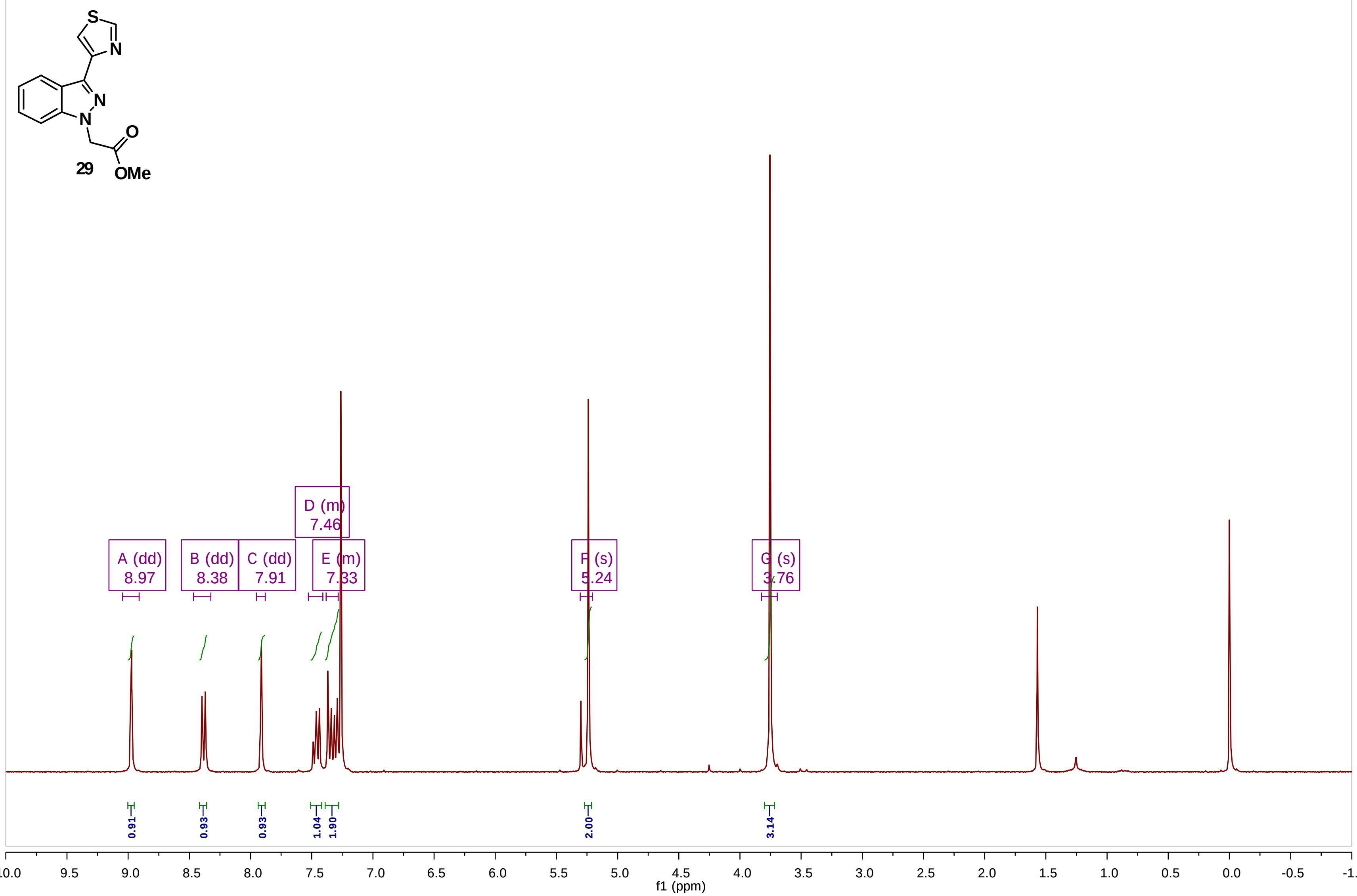

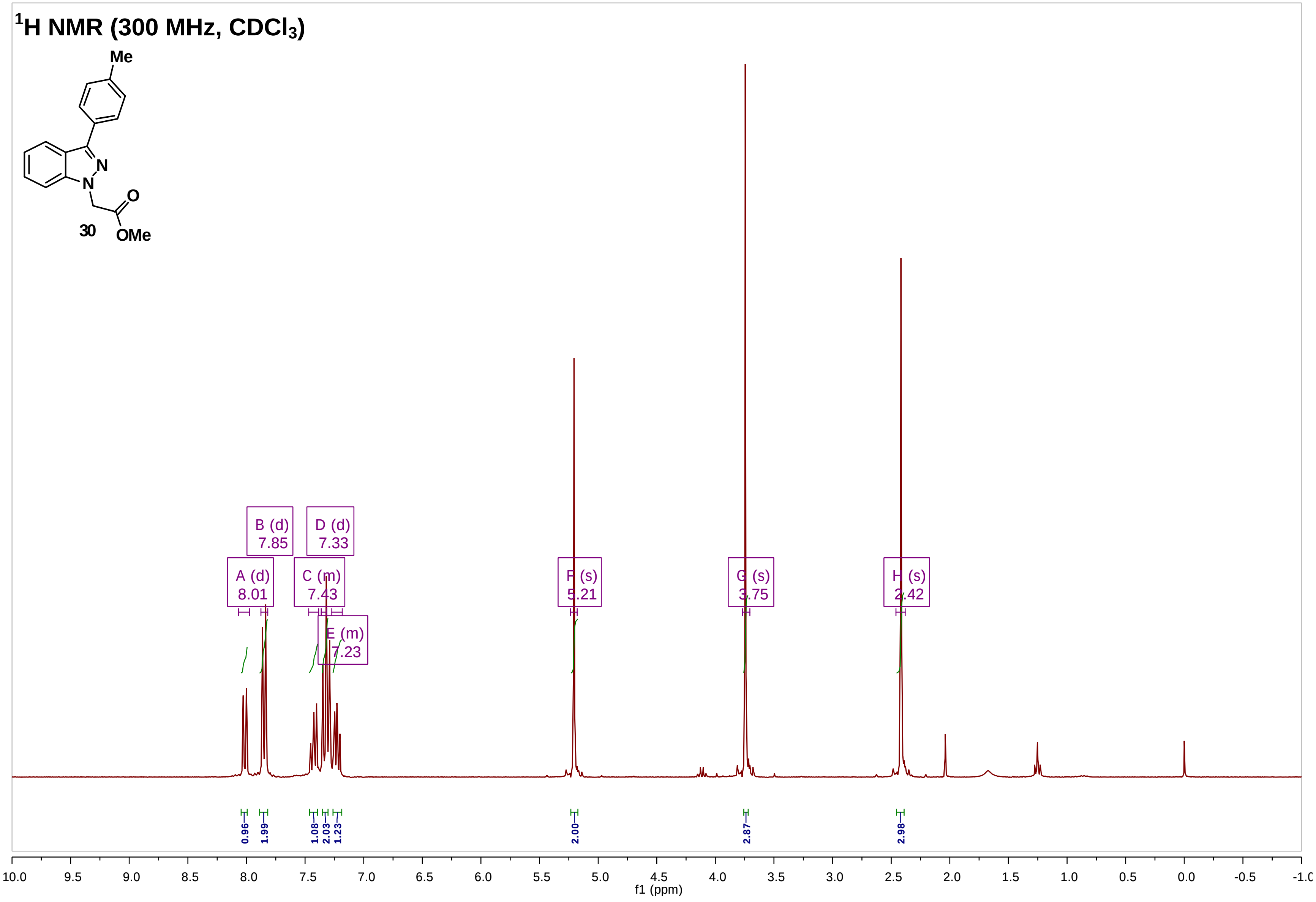

1H NMR (300 MHz, CDCl3)
Me
N
N
O
30
OMe
A (d)
8.01
B (d)
7.85
C (m)
7.43
D (d)
7.33
E (m)
7.23
F (s)
5.21
G (s)
3.75
H (s)
2.42
0.96
1.99
1.08
2.03
1.23
2.00
2.87
2.98
10.0
9.5
9.0
8.5
8.0
7.5
7.0
6.5
6.0
5.5
5.0
4.5
4.0
3.5
3.0
2.5
2.0
1.5
1.0
0.5
0.0
-0.5
f1 (ppm)

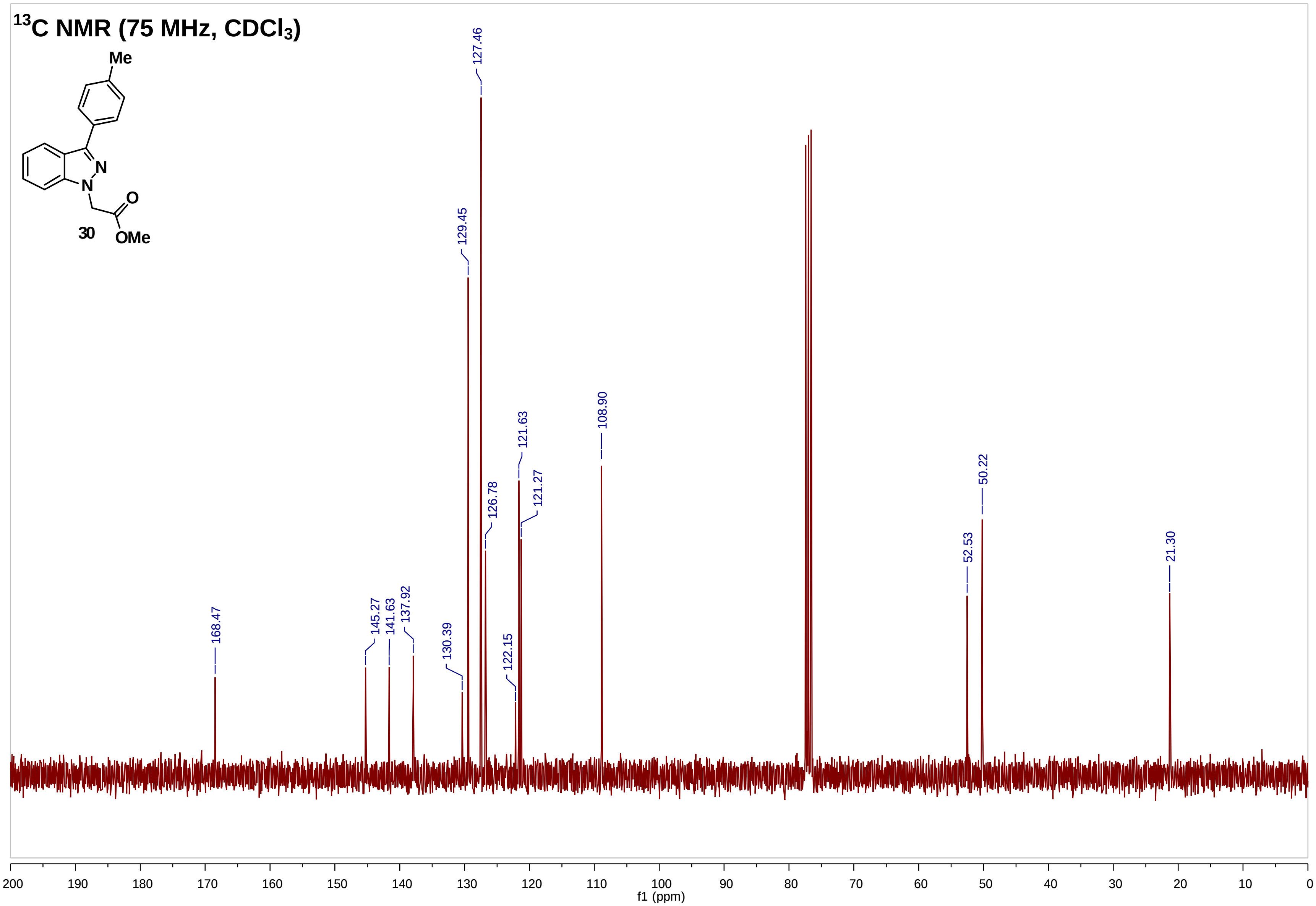
13C NMR (75 MHz, CDCl3)
Me
N
N
O
OMe
30
168.47
145.27
141.63
137.92
130.39
129.45
127.46
126.78
122.15
121.63
121.27
108.90
52.53
50.22
21.30
f1 (ppm)

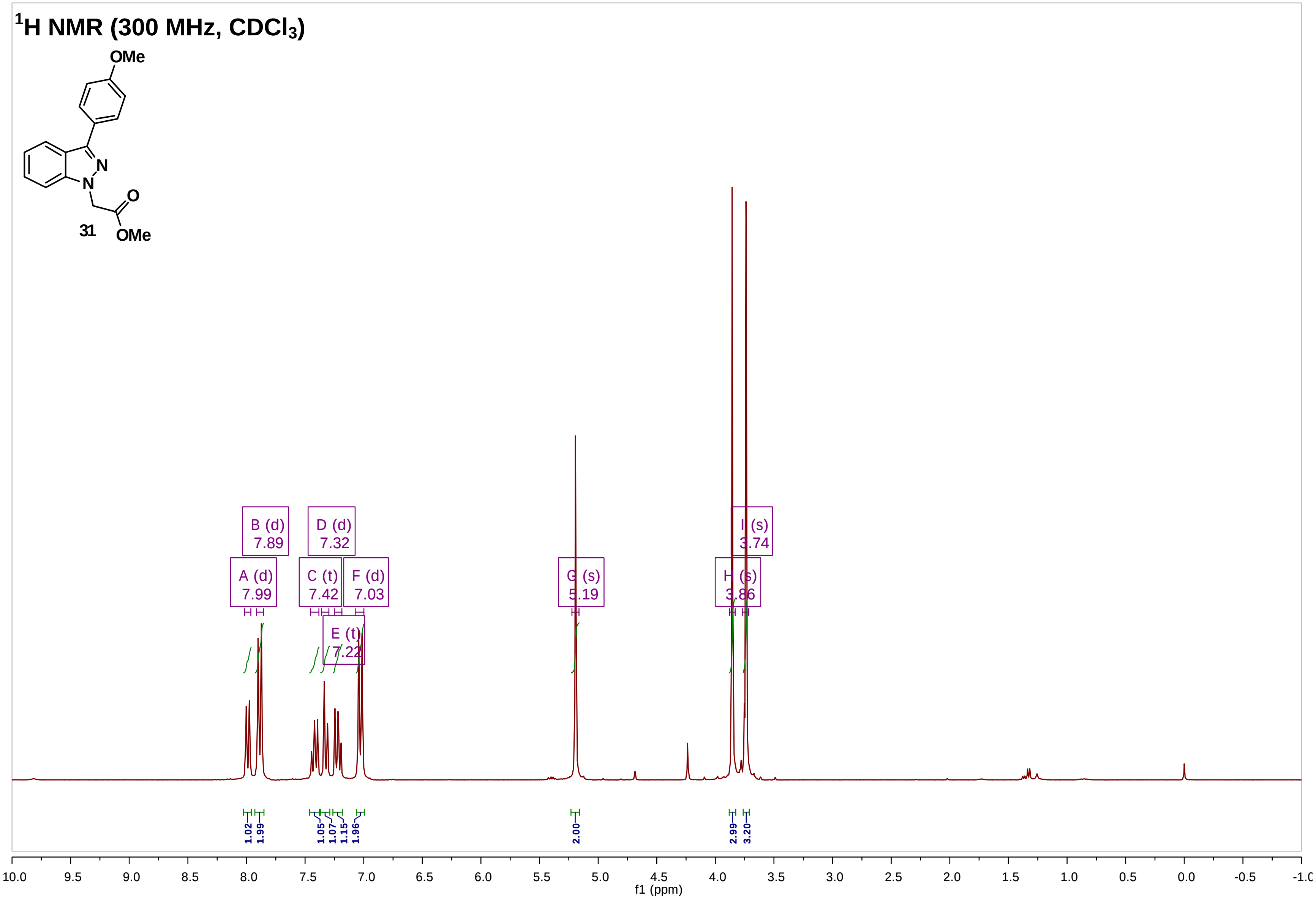
1H NMR (300 MHz, CDCl3)
OMe
N
N
O
31
OMe
A (d)
7.99
B (d)
7.89
C (t)
7.42
D (d)
7.32
E (t)
7.22
F (d)
7.03
G (s)
5.19
H (s)
3.86
I (s)
3.74
1.02
1.99
1.05
1.07
1.15
1.96
2.00
2.99
3.20
10.0
9.5
9.0
8.5
8.0
7.5
7.0
6.5
6.0
5.5
5.0
4.5
4.0
3.5
3.0
2.5
2.0
1.5
1.0
0.5
0.0
-0.5
f1 (ppm)

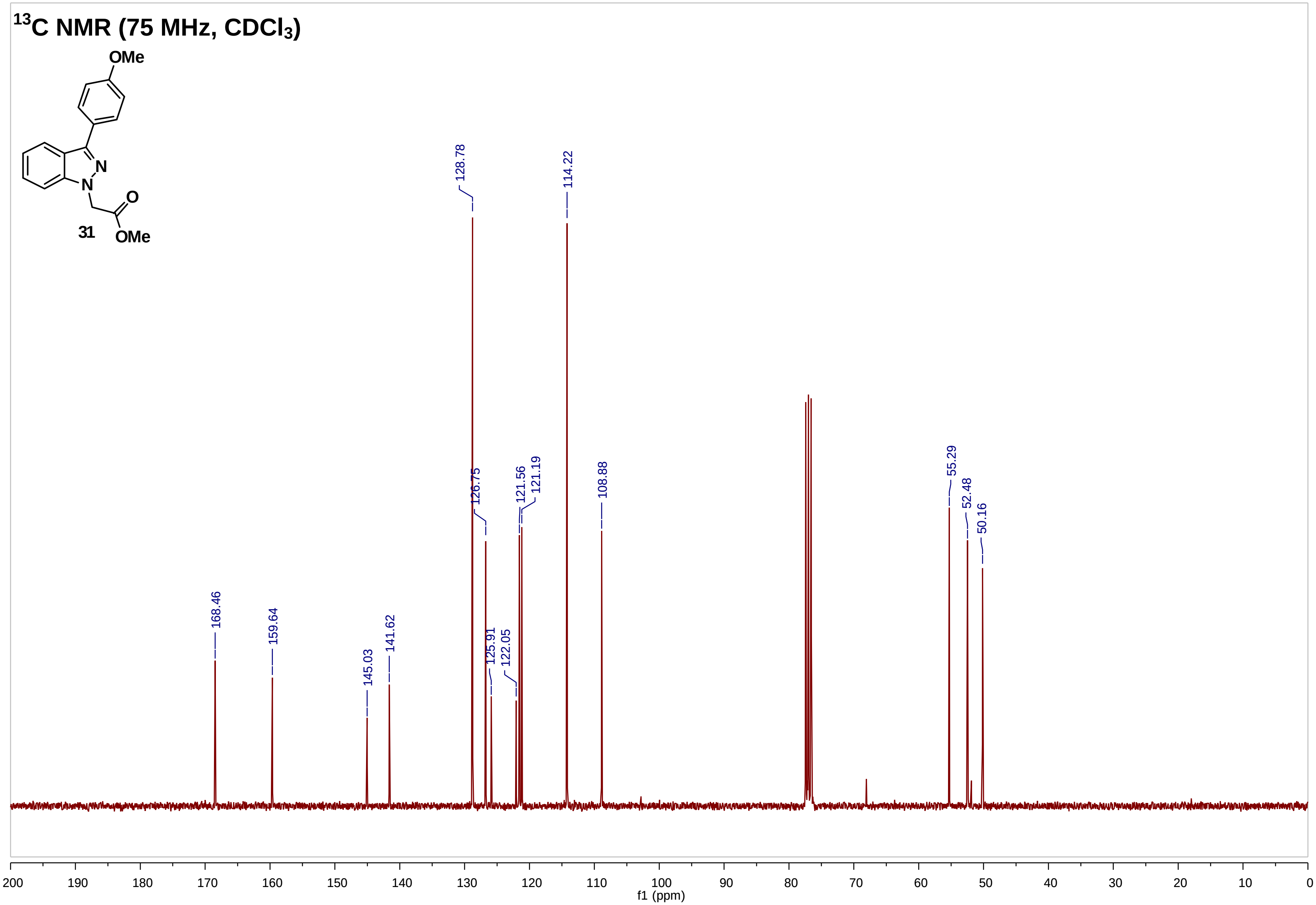

^{13}C NMR (75 MHz, $CDCl_3$)
OMe
N
N
O
31
OMe
168.46
159.64
145.03
141.62
128.78
126.75
125.91
122.05
121.56
121.19
114.22
108.88
55.29
52.48
50.16
200 190 180 170 160 150 140 130 120 110 100 90 80 70 60 50 40 30 20 10 0
f1 (ppm)

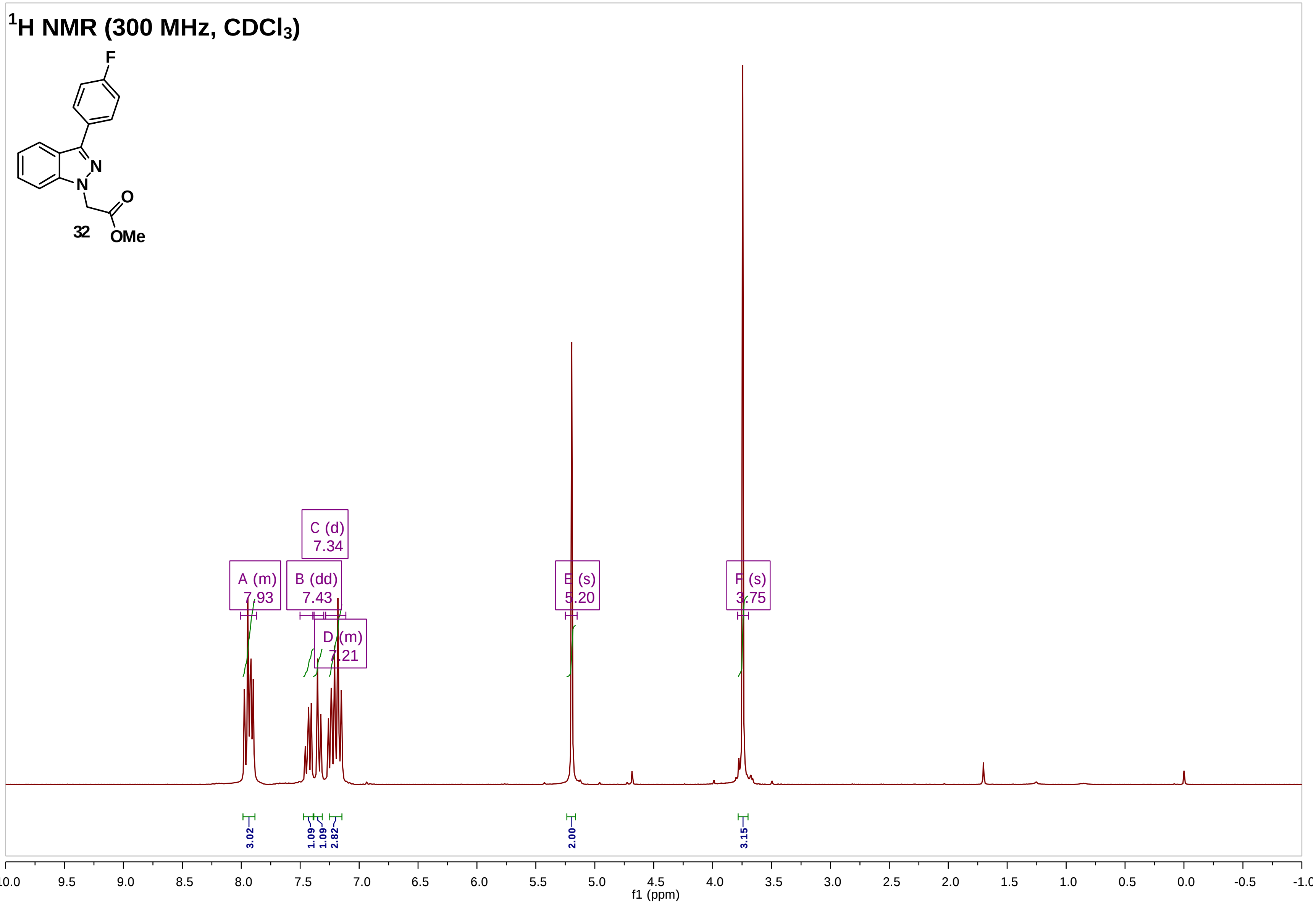

^{1}H NMR (300 MHz, CDCl$_3$)
F
N
N
O
32
OMe
C (d)
7.34
A (m)
7.93
B (dd)
7.43
E (s)
5.20
F (s)
3.75
D (m)
7.21
3.02
1.09
1.09
2.82
2.00
3.15
10.0
9.5
9.0
8.5
8.0
7.5
7.0
6.5
6.0
5.5
5.0
4.5
4.0
3.5
3.0
2.5
2.0
1.5
1.0
0.5
0.0
-0.5
f1 (ppm)

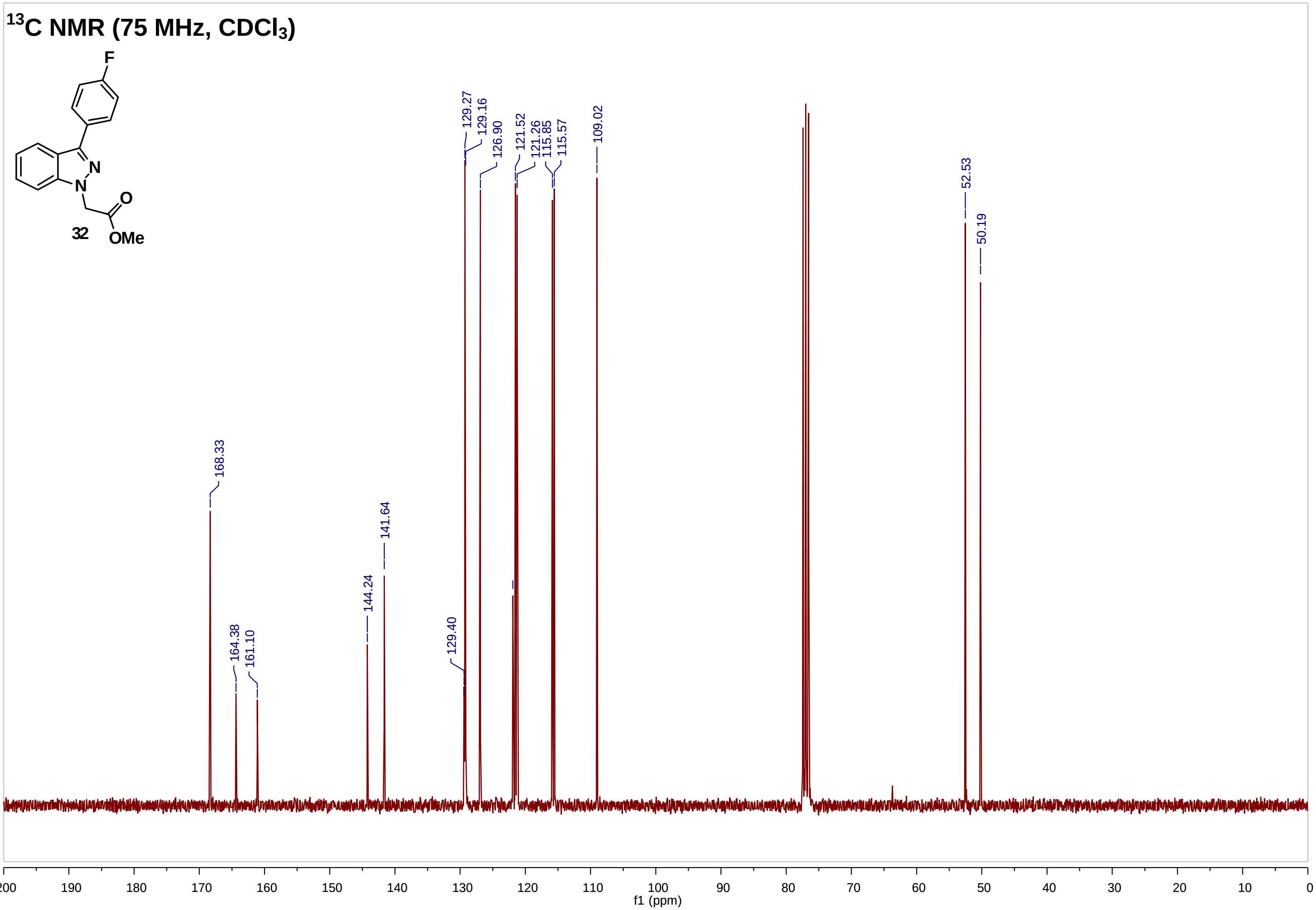

13C NMR (75 MHz, CDCl3)
F
N
N
O
32
OMe
168.33
164.38
161.10
144.24
141.64
129.40
129.27
129.16
126.90
121.52
121.26
115.85
115.57
109.02
52.53
50.19
200
190
180
170
160
150
140
130
120
110
100
90
80
70
60
50
40
30
20
10
0
f1 (ppm)

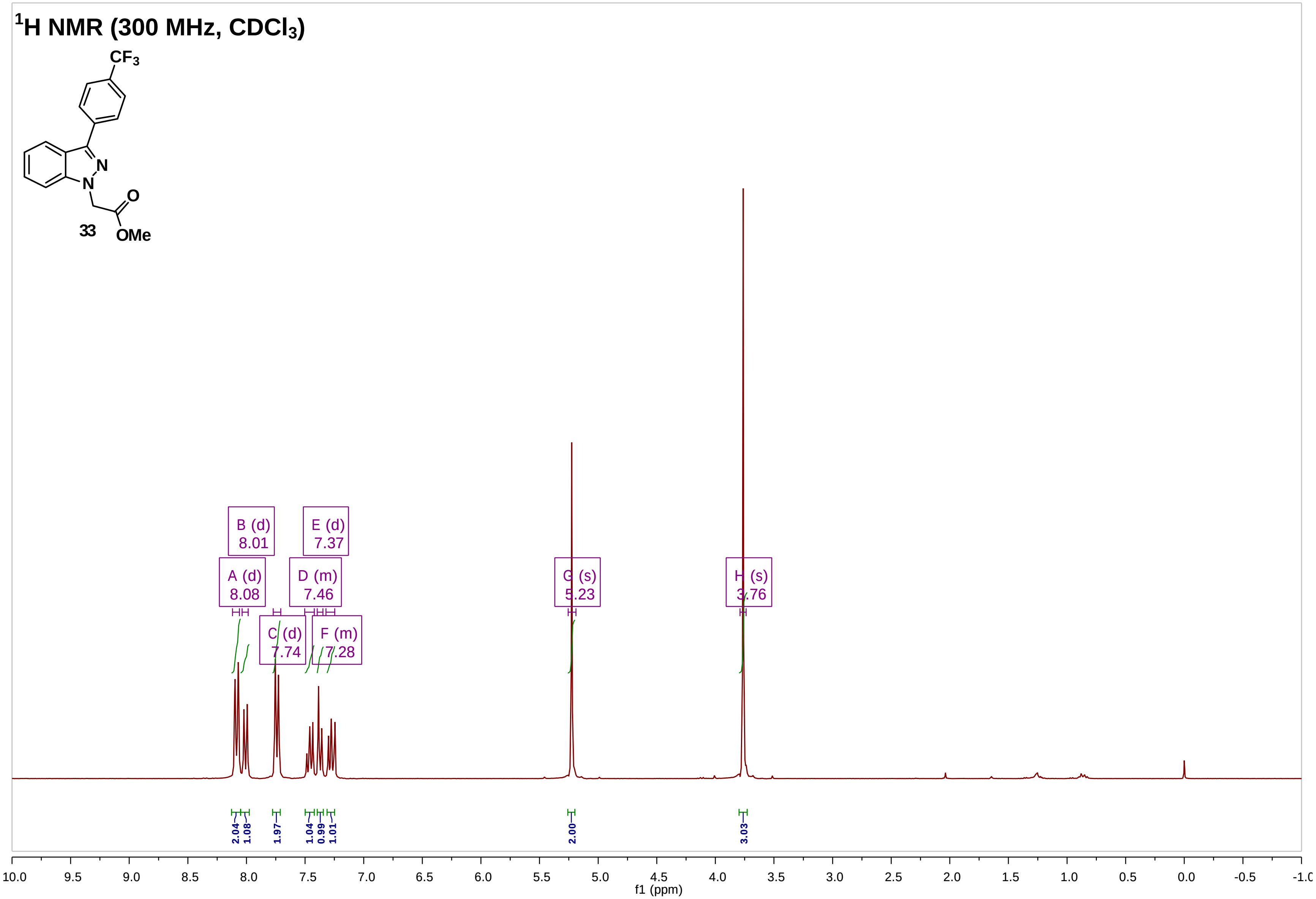

1H NMR (300 MHz, CDCl3)
CF3
N
N
O
33
OMe
B (d) 8.01
E (d) 7.37
A (d) 8.08
D (m) 7.46
G (s) 5.23
H (s) 3.76
C (d) 7.74
F (m) 7.28
2.04
1.08
1.97
1.04
0.99
1.01
2.00
3.03
10.0 9.5 9.0 8.5 8.0 7.5 7.0 6.5 6.0 5.5 5.0 4.5 4.0 3.5 3.0 2.5 2.0 1.5 1.0 0.5 0.0 -0.5
f1 (ppm)

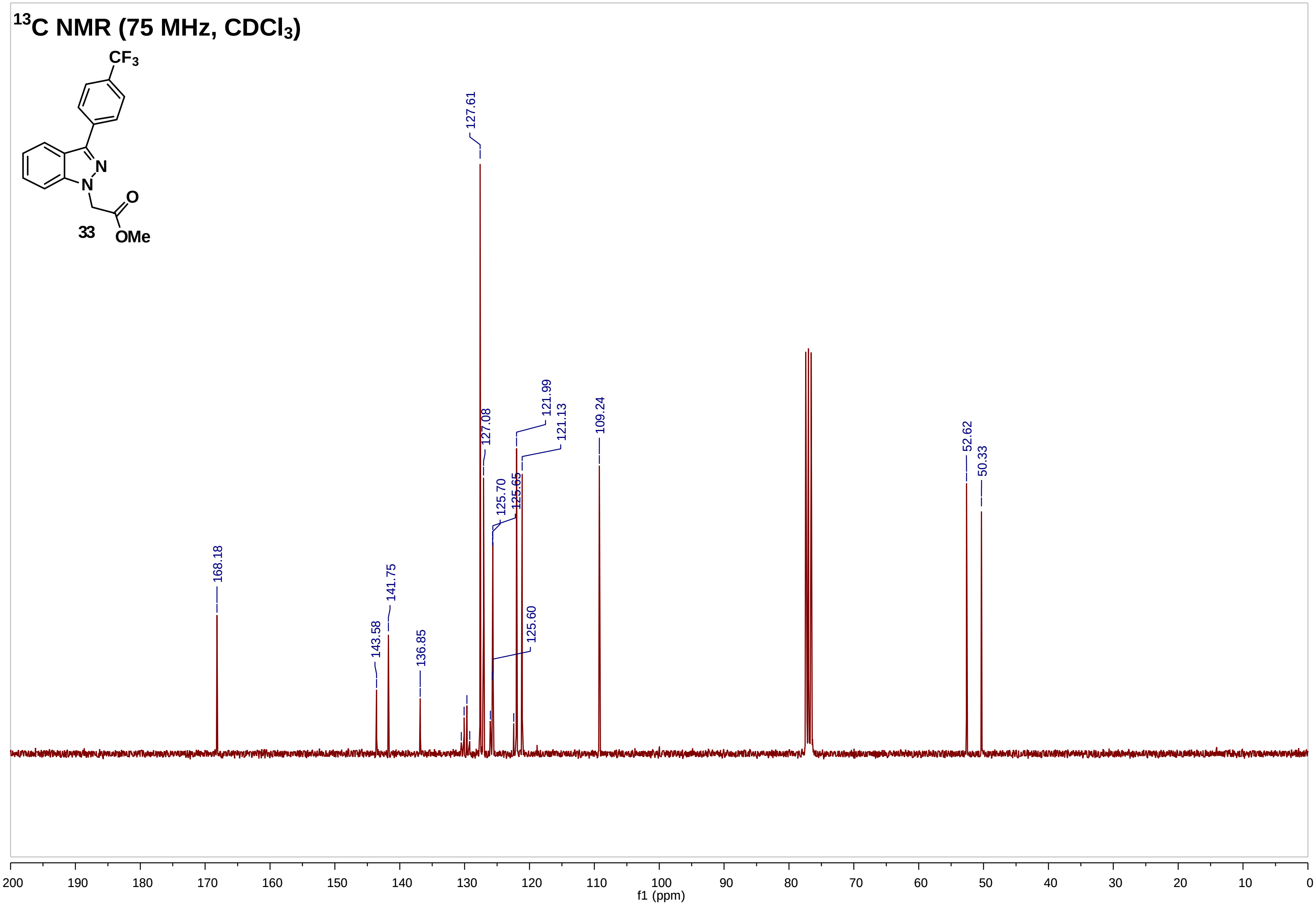

^{13}C NMR (75 MHz, $CDCl_3$)
CF_3
N
N
O
OMe
33
127.61
127.08
125.70
125.65
121.99
121.13
109.24
168.18
141.75
143.58
136.85
125.60
52.62
50.33
200 190 180 170 160 150 140 130 120 110 100 90 80 70 60 50 40 30 20 10 0
f1 (ppm)

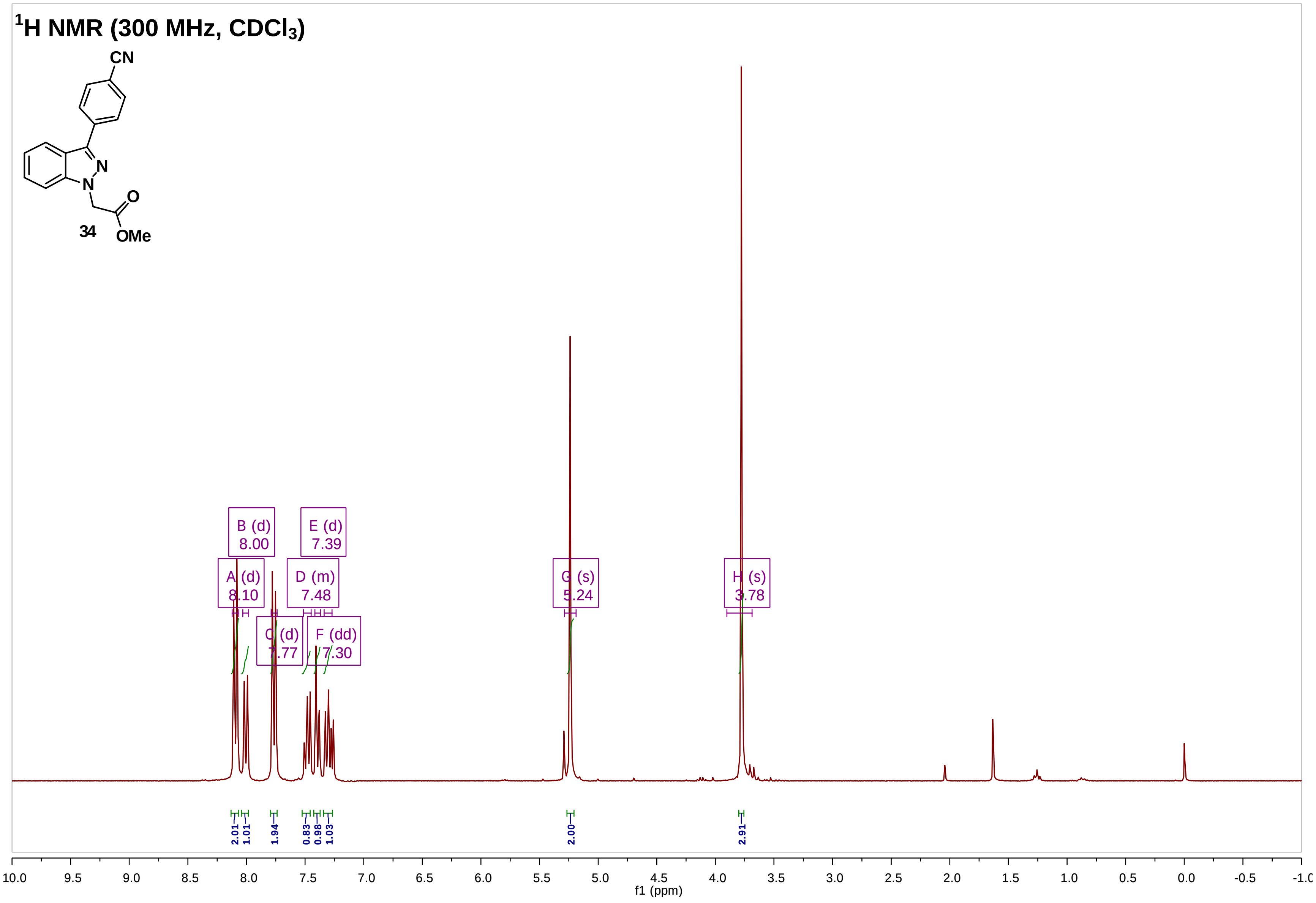

1H NMR (300 MHz, CDCl3)
CN
N
N
O
34
OMe
A (d)
8.10
B (d)
8.00
C (d)
7.77
D (m)
7.48
E (d)
7.39
F (dd)
7.30
G (s)
5.24
H (s)
3.78
2.01
1.01
1.94
0.83
0.98
1.03
2.00
2.91
f1 (ppm)

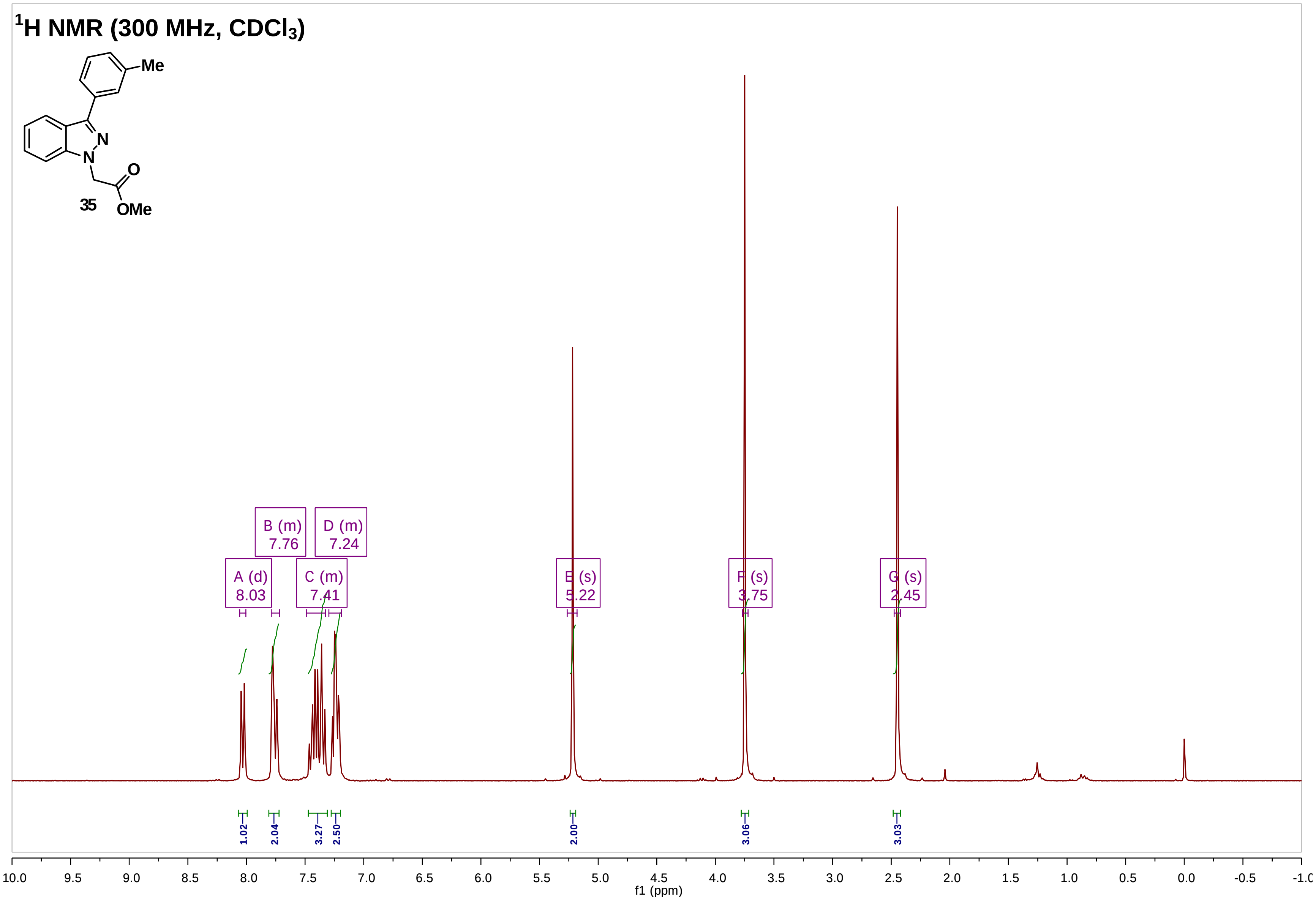

1H NMR (300 MHz, CDCl3)
Me
N
N
O
OMe
35
A (d)
8.03
B (m)
7.76
C (m)
7.41
D (m)
7.24
E (s)
5.22
F (s)
3.75
G (s)
2.45
1.02
2.04
3.27
2.50
2.00
3.06
3.03
10.0
9.5
9.0
8.5
8.0
7.5
7.0
6.5
6.0
5.5
5.0
4.5
4.0
3.5
3.0
2.5
2.0
1.5
1.0
0.5
0.0
-0.5
f1 (ppm)

^{13}C NMR (75 MHz, $CDCl_3$)

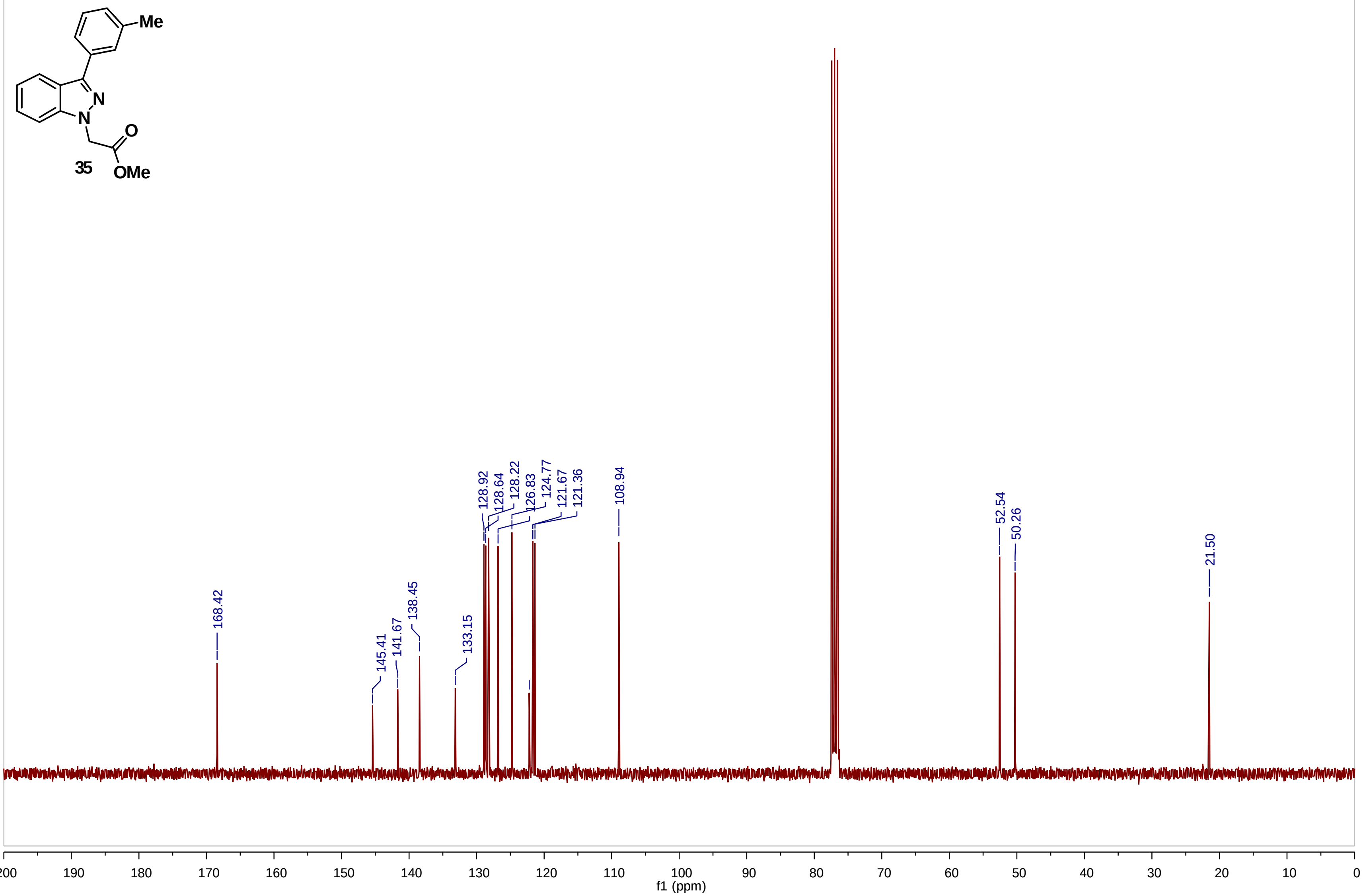

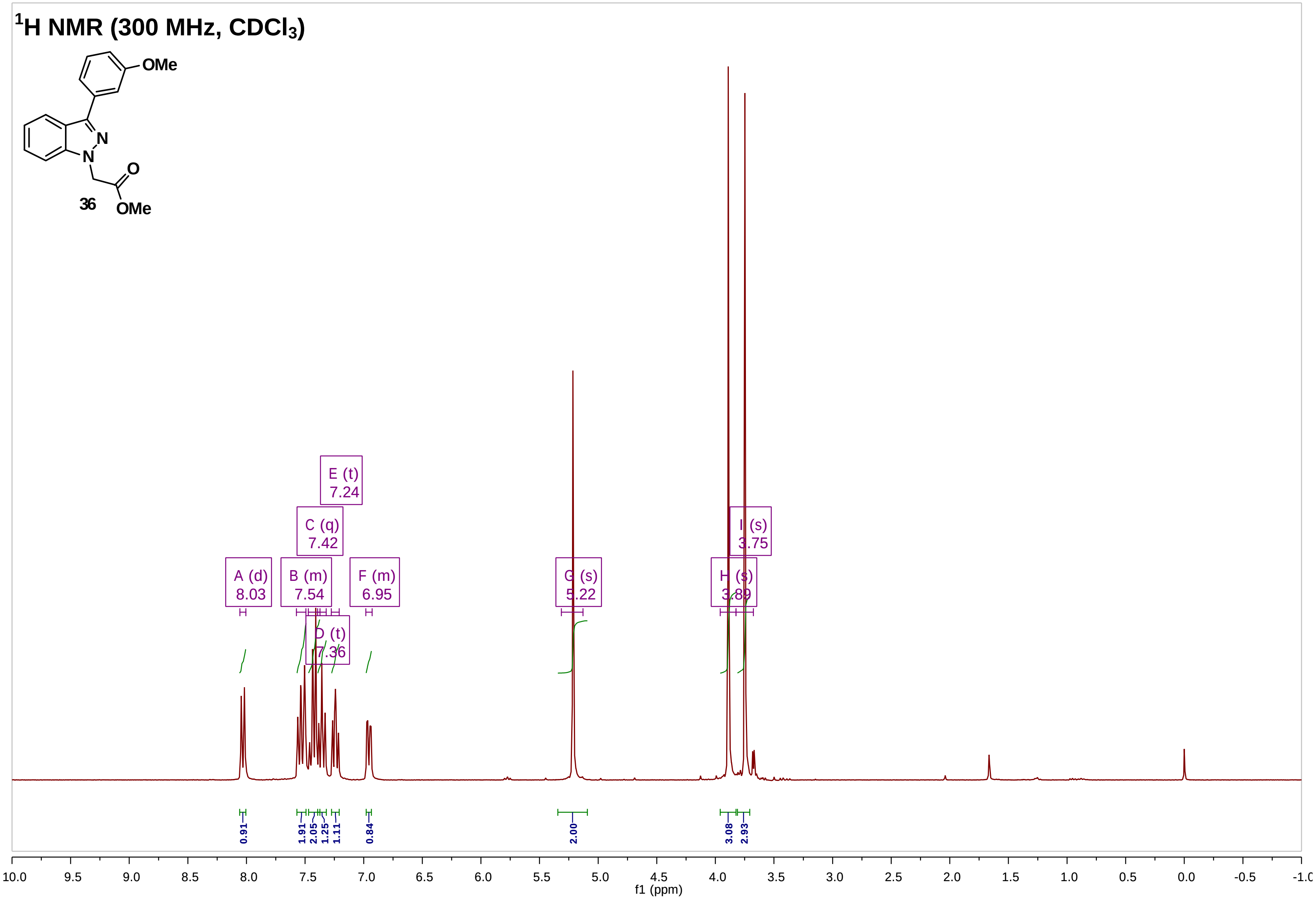
1H NMR (300 MHz, CDCl3)
OMe
N
N
O
36
OMe
A (d)
8.03
B (m)
7.54
C (q)
7.42
D (t)
7.36
E (t)
7.24
F (m)
6.95
G (s)
5.22
H (s)
3.89
I (s)
3.75
0.91
1.91
2.05
1.25
1.11
0.84
2.00
3.08
2.93
10.0
9.5
9.0
8.5
8.0
7.5
7.0
6.5
6.0
5.5
5.0
4.5
4.0
3.5
3.0
2.5
2.0
1.5
1.0
0.5
0.0
-0.5
f1 (ppm)

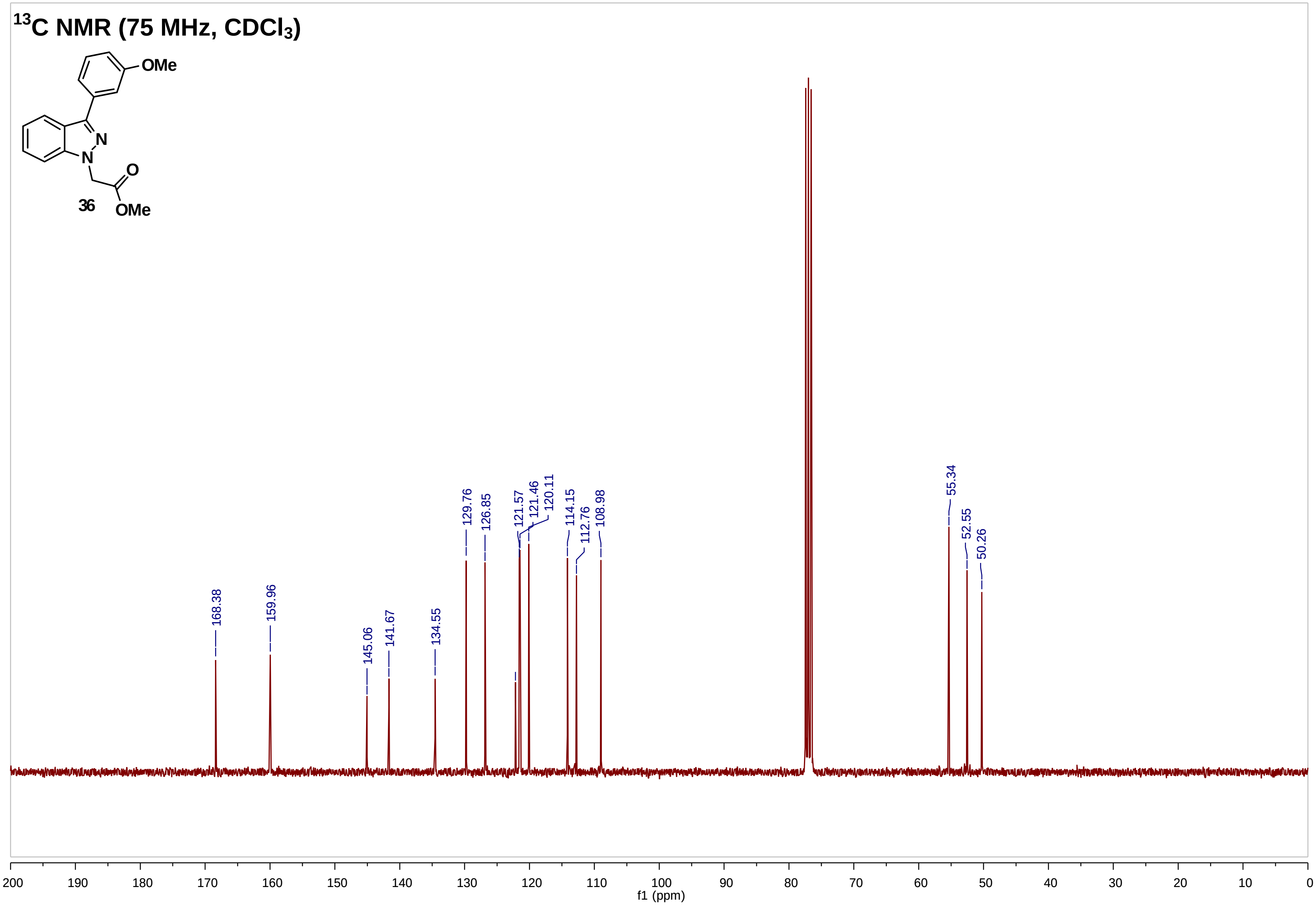

13C NMR (75 MHz, CDCl3)
OMe
N
N
O
36
OMe
168.38
159.96
145.06
141.67
134.55
129.76
126.85
121.57
121.46
120.11
114.15
112.76
108.98
55.34
52.55
50.26
f1 (ppm)
200
190
180
170
160
150
140
130
120
110
100
90
80
70
60
50
40
30
20
10
0

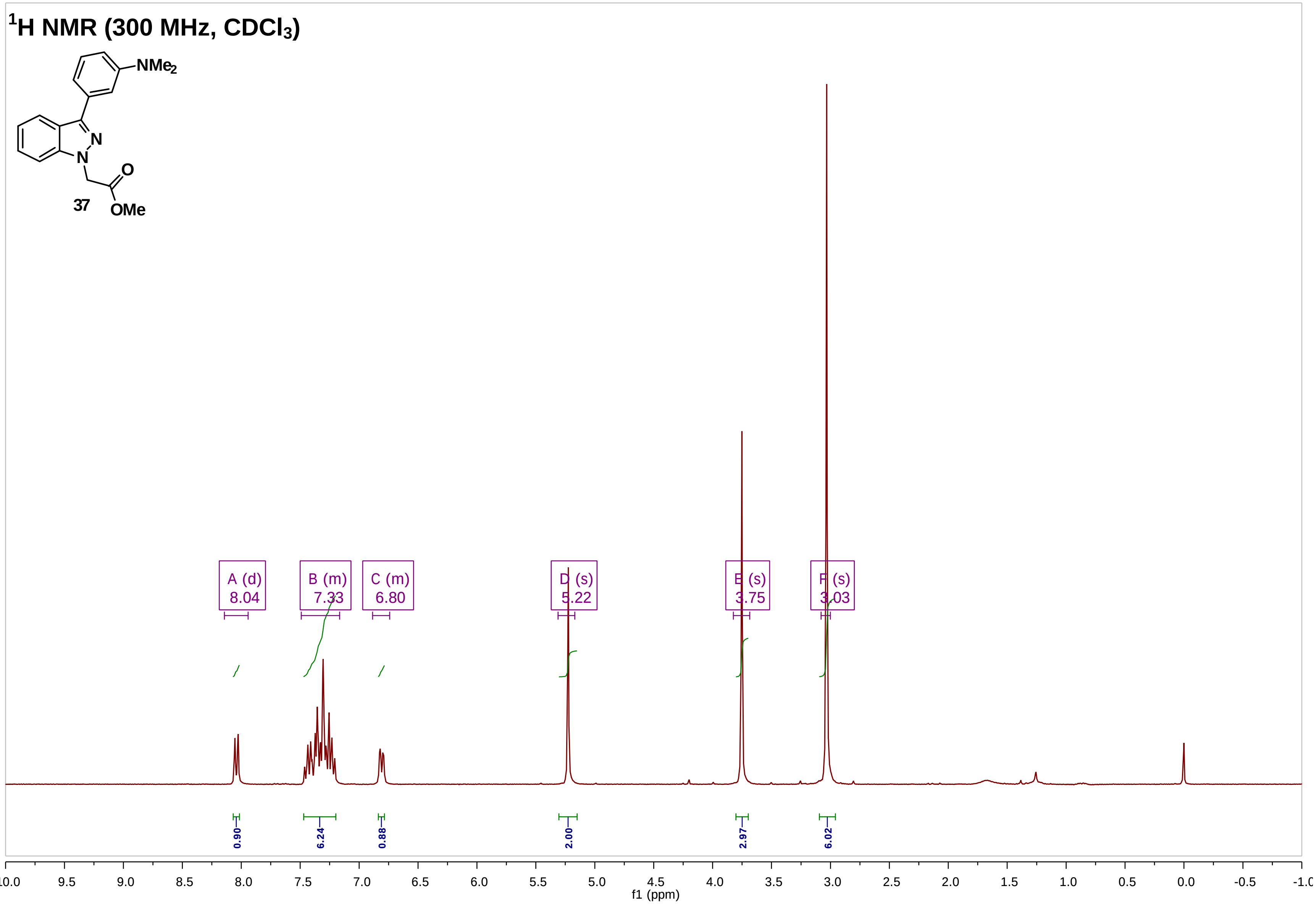

1H NMR (300 MHz, CDCl3)
NMe2
N
N
O
37
OMe
A (d)
8.04
B (m)
7.33
C (m)
6.80
D (s)
5.22
E (s)
3.75
F (s)
3.03
0.90
6.24
0.88
2.00
2.97
6.02
10.0
9.5
9.0
8.5
8.0
7.5
7.0
6.5
6.0
5.5
5.0
4.5
4.0
3.5
3.0
2.5
2.0
1.5
1.0
0.5
0.0
-0.5
f1 (ppm)

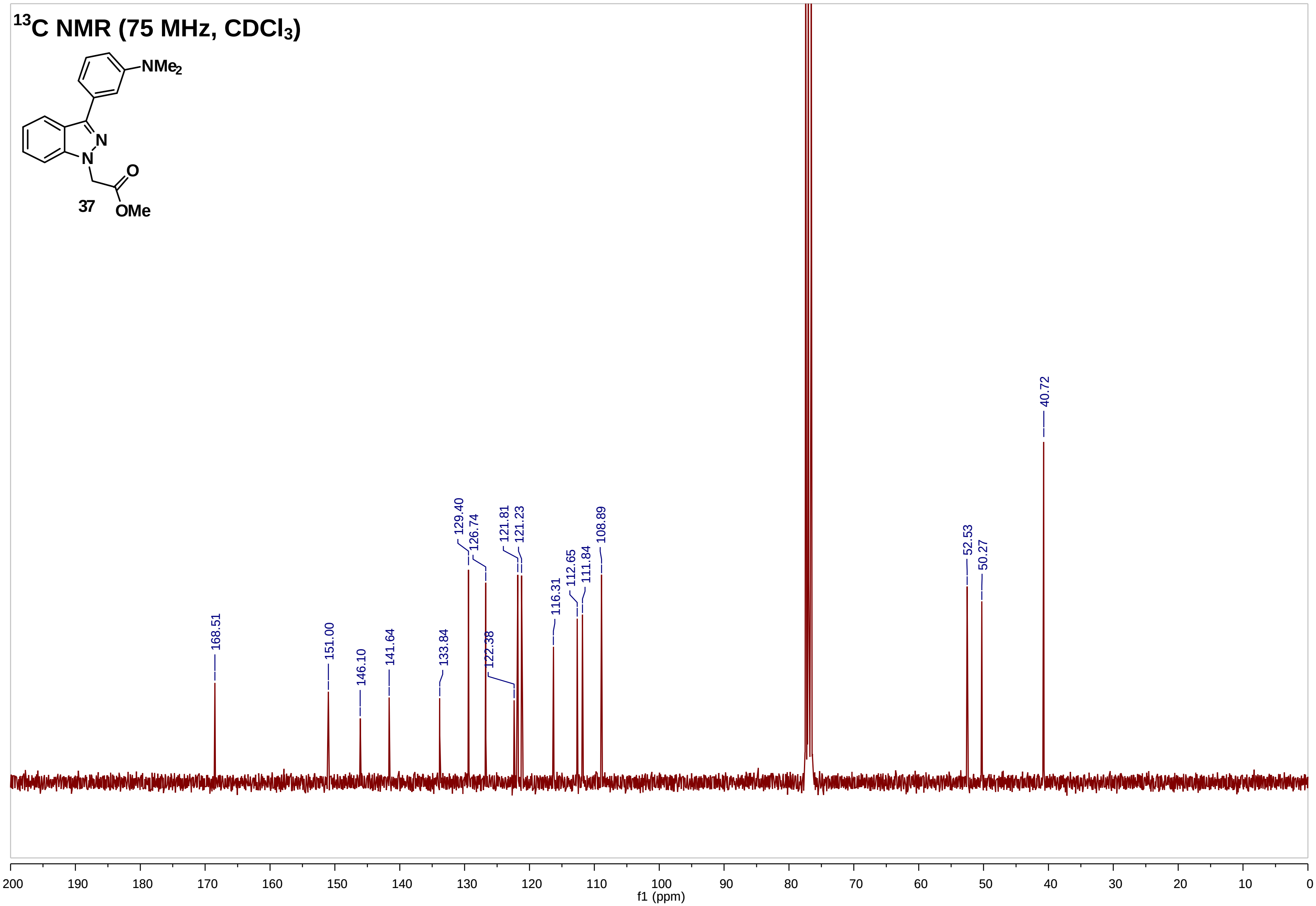
13C NMR (75 MHz, CDCl3)
NMe2
N
N
O
OMe
37
168.51
151.00
146.10
141.64
133.84
129.40
126.74
122.38
121.81
121.23
116.31
112.65
111.84
108.89
52.53
50.27
40.72
200
190
180
170
160
150
140
130
120
110
100
90
80
70
60
50
40
30
20
10
0
f1 (ppm)

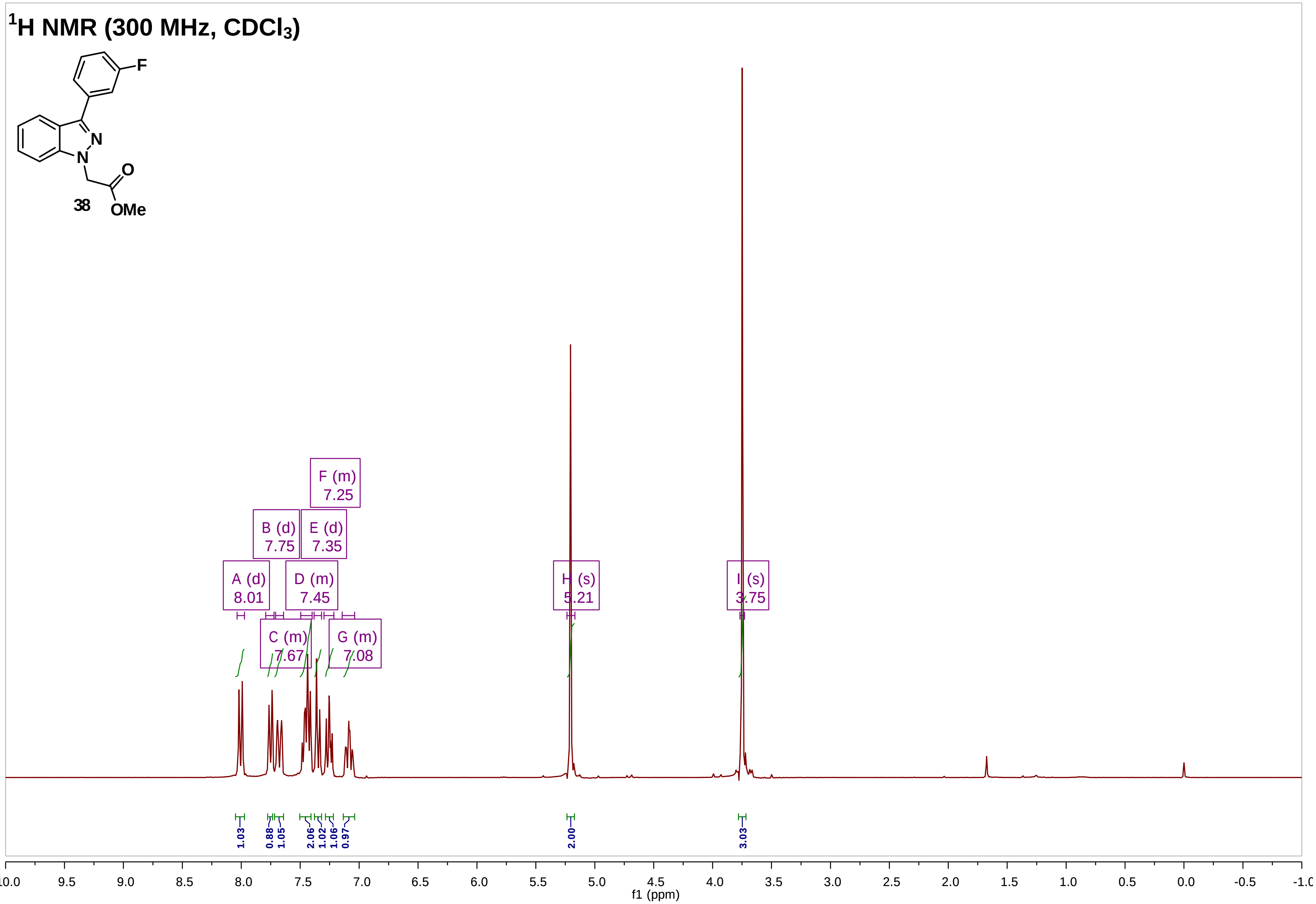
^{1}H NMR (300 MHz, $CDCl_3$)
F
N
N
O
38
OMe
F (m)
7.25
B (d)
7.75
E (d)
7.35
A (d)
8.01
D (m)
7.45
H (s)
5.21
I (s)
3.75
C (m)
7.67
G (m)
7.08
1.03
0.88
1.05
2.06
1.02
1.06
0.97
2.00
3.03
10.0
9.5
9.0
8.5
8.0
7.5
7.0
6.5
6.0
5.5
5.0
4.5
4.0
3.5
3.0
2.5
2.0
1.5
1.0
0.5
0.0
-0.5
f1 (ppm)

^{13}C NMR (75 MHz, $CDCl_3$)

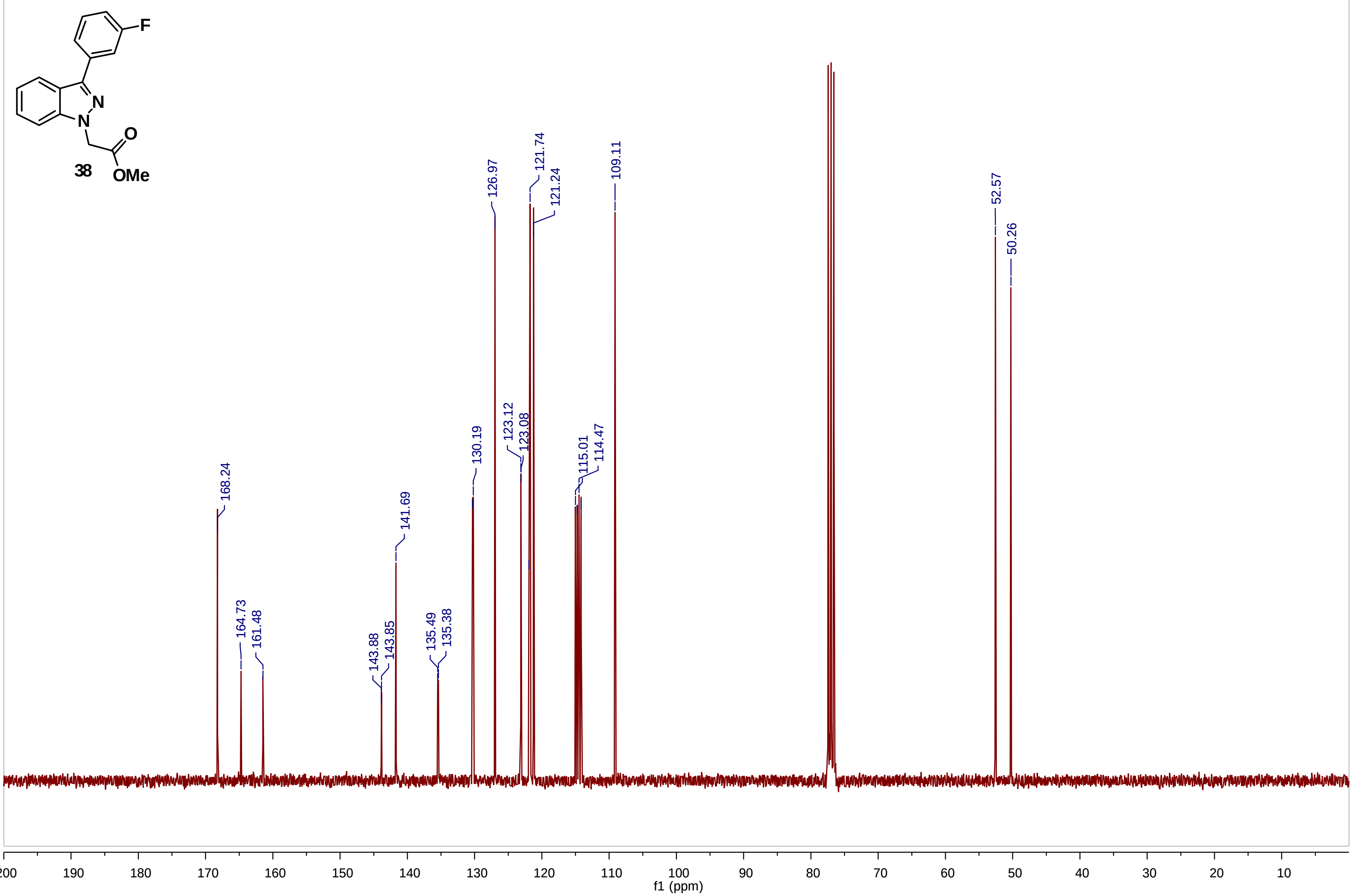

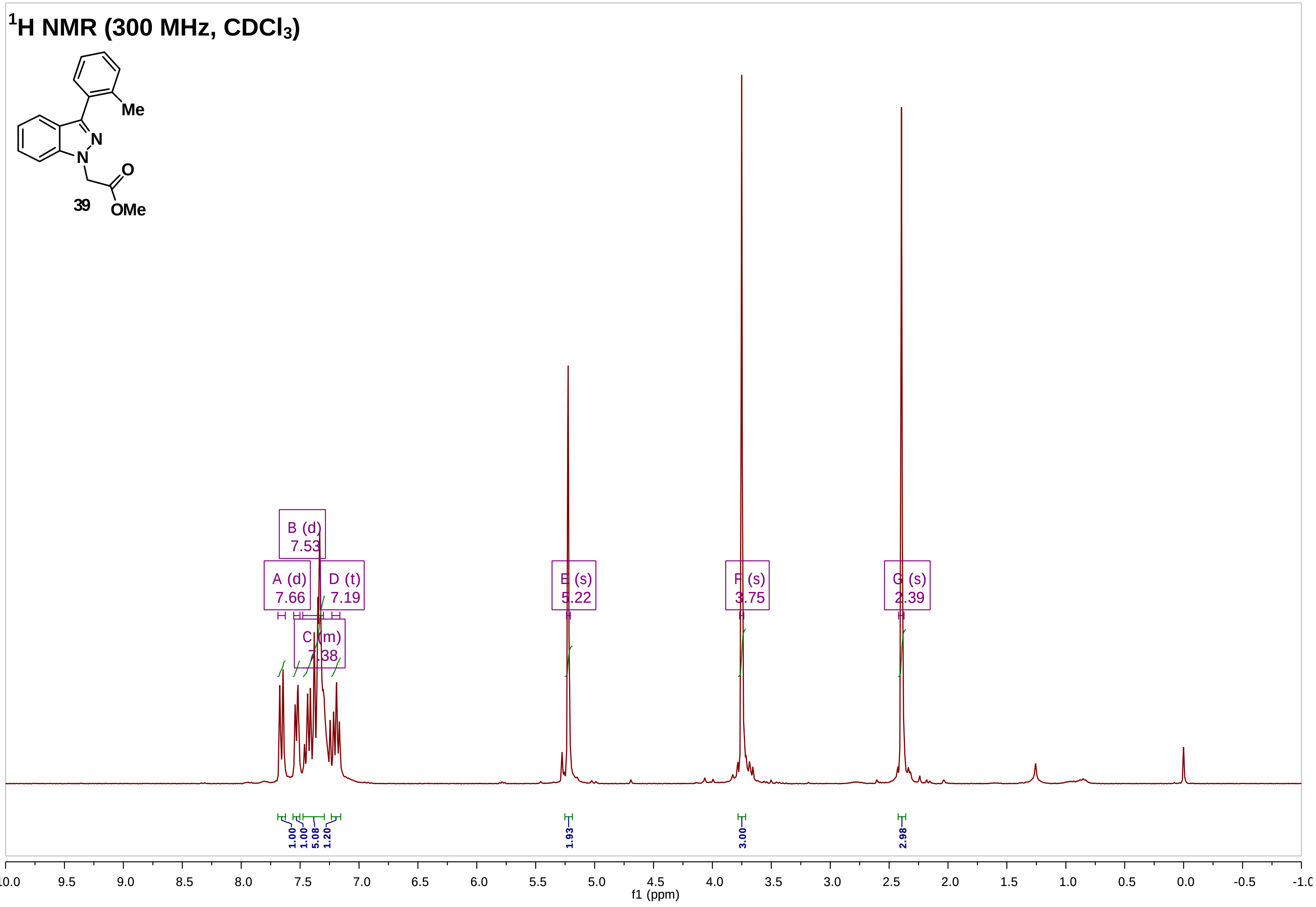

1H NMR (300 MHz, CDCl3)
Me
N
N
O
OMe
39
B (d) 7.53
A (d) 7.66
D (t) 7.19
C (m) 7.38
E (s) 5.22
F (s) 3.75
G (s) 2.39
1.00
1.00
5.08
1.20
1.93
3.00
2.98
10.0 9.5 9.0 8.5 8.0 7.5 7.0 6.5 6.0 5.5 5.0 4.5 4.0 3.5 3.0 2.5 2.0 1.5 1.0 0.5 0.0 -0.5 -1.0
f1 (ppm)

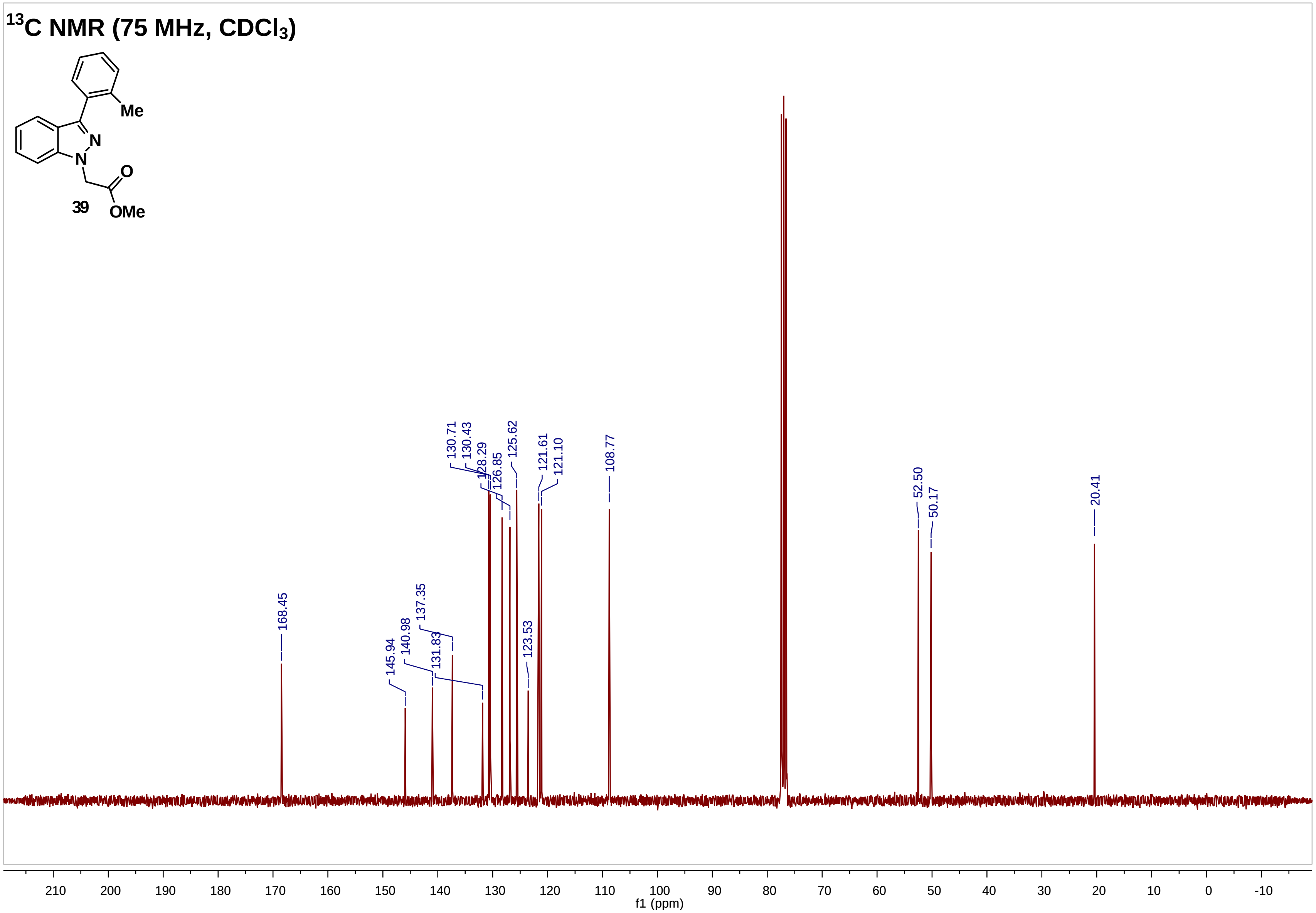

^{13}C NMR (75 MHz, CDCl$_3$)
Me
N
N
O
39
OMe
168.45
145.94
140.98
137.35
131.83
130.71
130.43
128.29
126.85
125.62
123.53
121.61
121.10
108.77
52.50
50.17
20.41
210
200
190
180
170
160
150
140
130
120
110
100
90
80
70
60
50
40
30
20
10
0
-10
f1 (ppm)

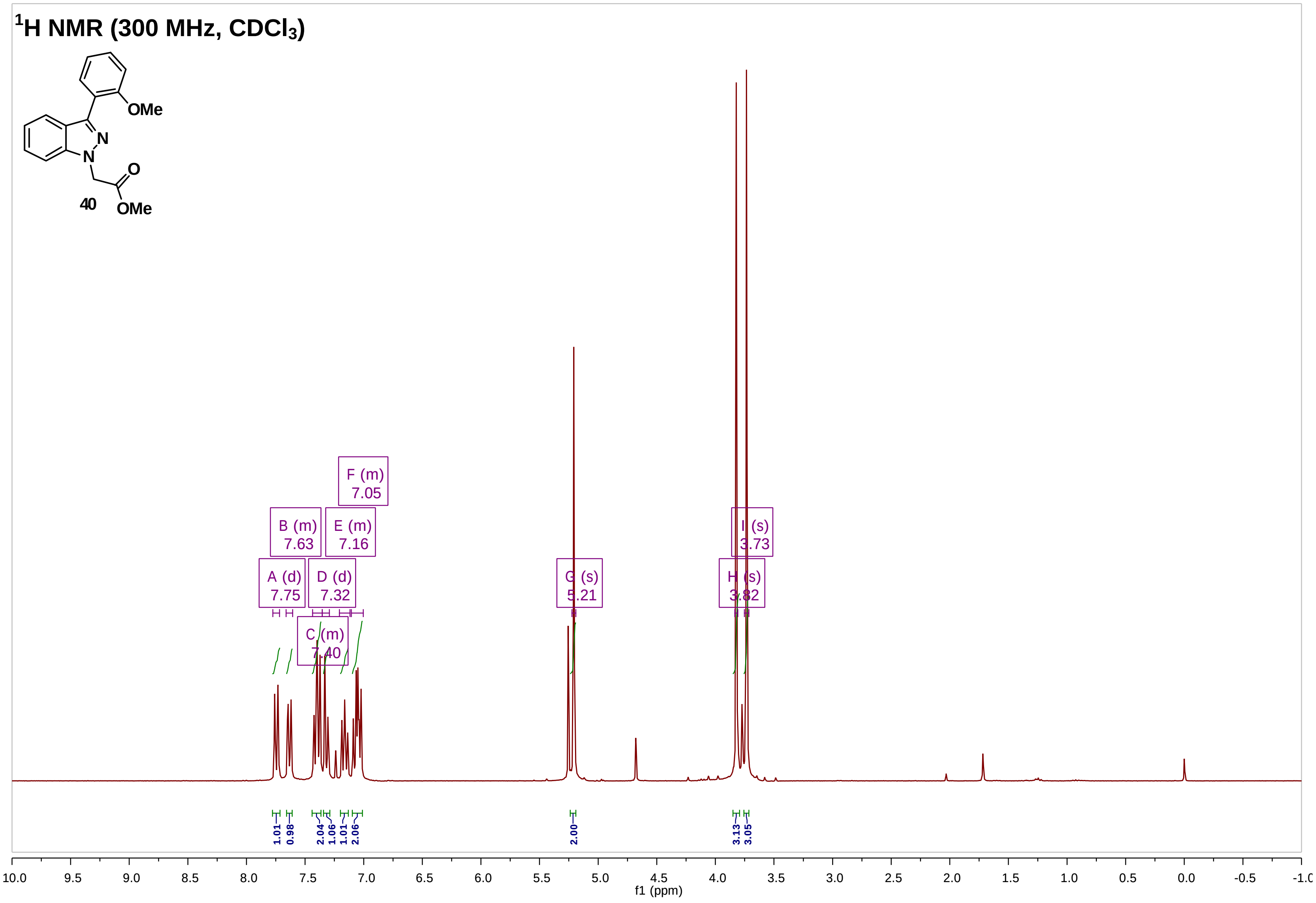

^{1}H NMR (300 MHz, $CDCl_3$)
OMe
N
N
O
40
OMe
A (d) 7.75
B (m) 7.63
C (m) 7.40
D (d) 7.32
E (m) 7.16
F (m) 7.05
G (s) 5.21
H (s) 3.82
I (s) 3.73
1.01
0.98
2.04
1.06
1.01
2.06
2.00
3.13
3.05
10.0 9.5 9.0 8.5 8.0 7.5 7.0 6.5 6.0 5.5 5.0 4.5 4.0 3.5 3.0 2.5 2.0 1.5 1.0 0.5 0.0 -0.5
f1 (ppm)

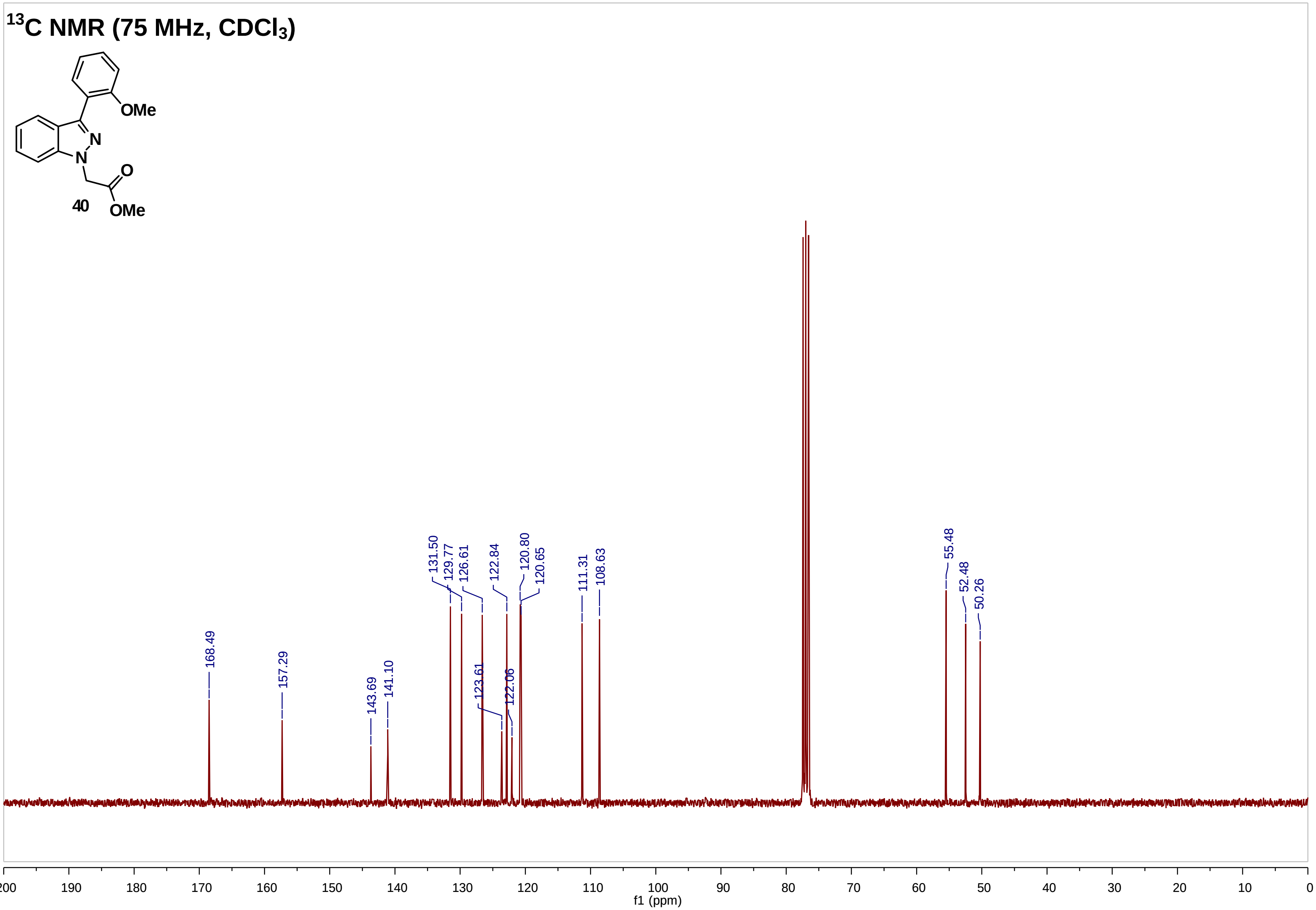

13C NMR (75 MHz, CDCl3)
OMe
N
N
O
OMe
40
168.49
157.29
143.69
141.10
131.50
129.77
126.61
123.61
122.84
122.06
120.80
120.65
111.31
108.63
55.48
52.48
50.26
200
190
180
170
160
150
140
130
120
110
100
90
80
70
60
50
40
30
20
10
0
f1 (ppm)

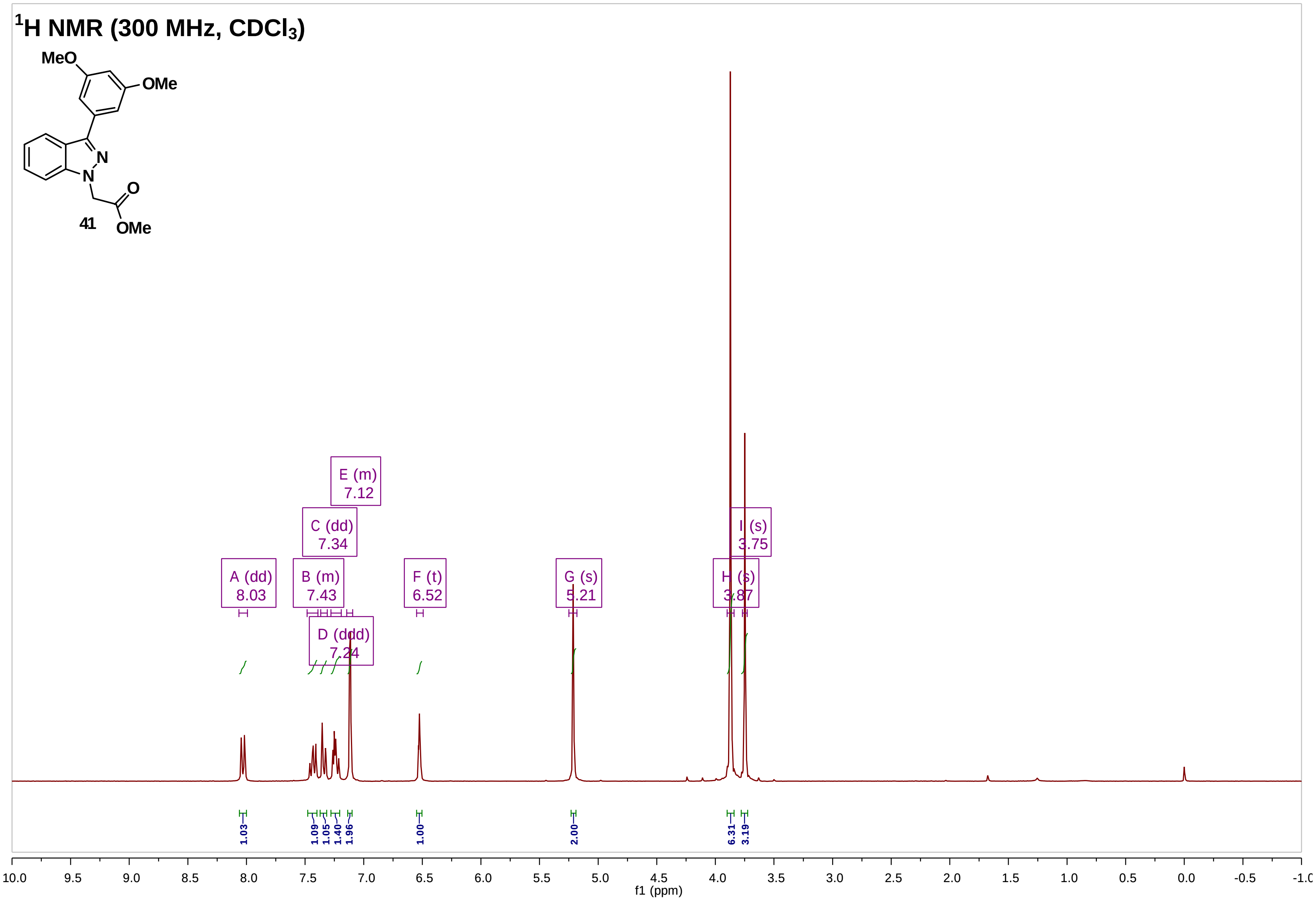

1H NMR (300 MHz, CDCl3)
MeO
OMe
N
N
O
OMe
41
A (dd) 8.03
B (m) 7.43
C (dd) 7.34
D (ddd) 7.24
E (m) 7.12
F (t) 6.52
G (s) 5.21
H (s) 3.87
I (s) 3.75
1.03
1.09
1.05
1.40
1.96
1.00
2.00
6.31
3.19
f1 (ppm)

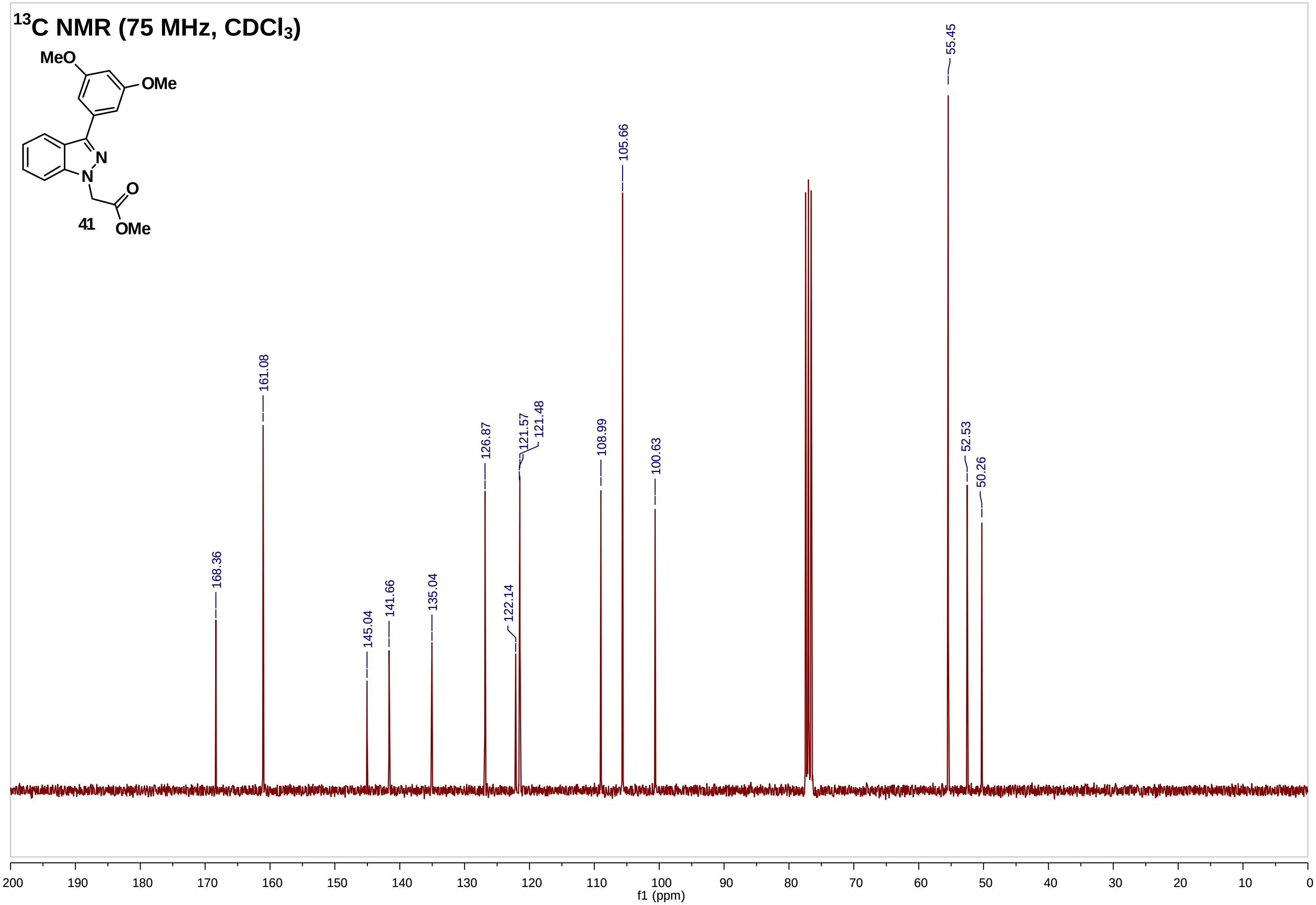

13C NMR (75 MHz, CDCl3)
MeO
OMe
N
N
O
OMe
41
168.36
161.08
145.04
141.66
135.04
126.87
122.14
121.57
121.48
108.99
105.66
100.63
55.45
52.53
50.26
200
190
180
170
160
150
140
130
120
110
100
90
80
70
60
50
40
30
20
10
0
f1 (ppm)

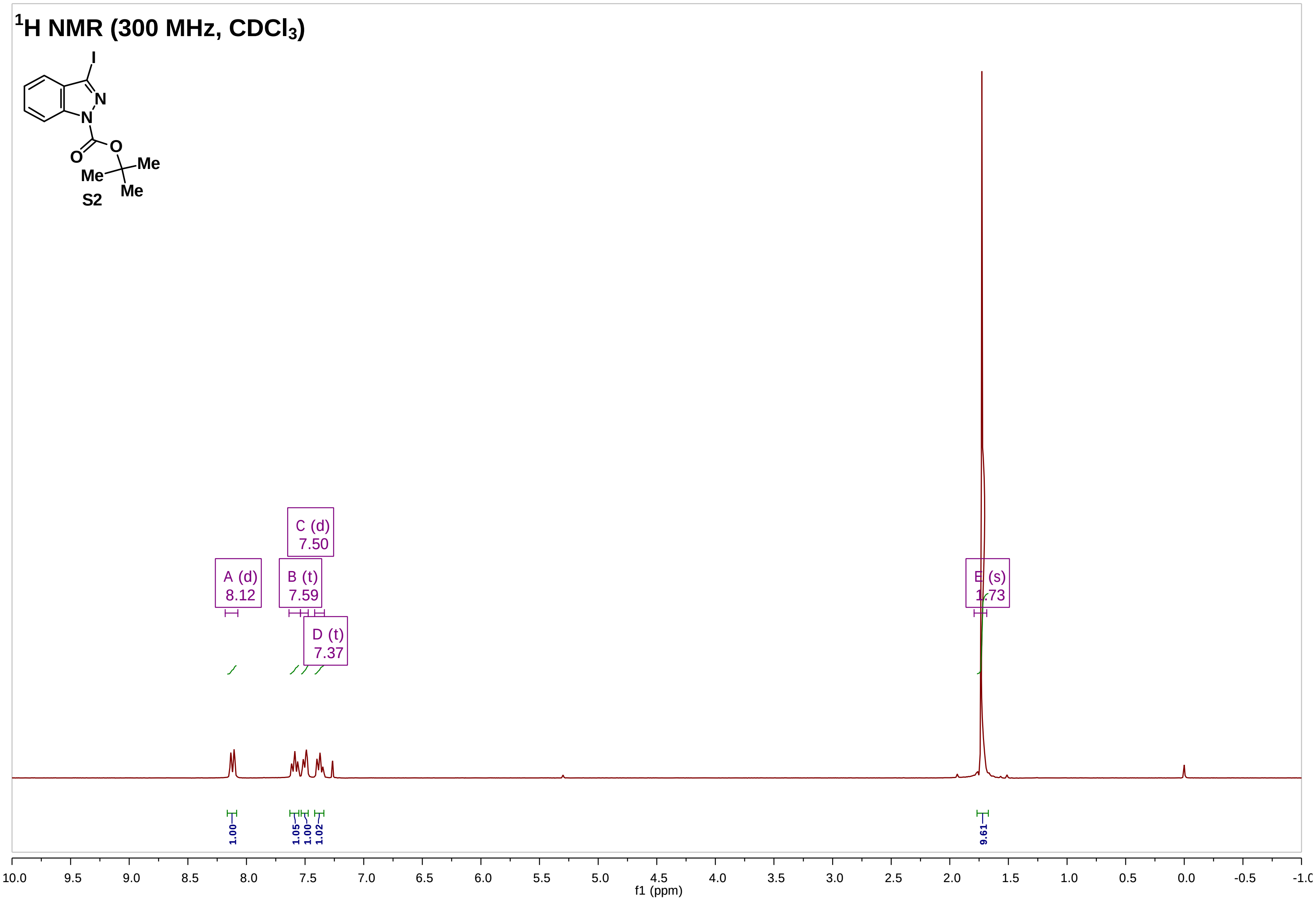

1H NMR (300 MHz, CDCl3)
I
N
N
O
O
Me
Me
Me
S2
A (d) 8.12
B (t) 7.59
C (d) 7.50
D (t) 7.37
E (s) 1.73
1.00
1.05
1.00
1.02
9.61
10.0 9.5 9.0 8.5 8.0 7.5 7.0 6.5 6.0 5.5 5.0 4.5 4.0 3.5 3.0 2.5 2.0 1.5 1.0 0.5 0.0 -0.5
f1 (ppm)

^{13}C NMR (75 MHz, $CDCl_3$)

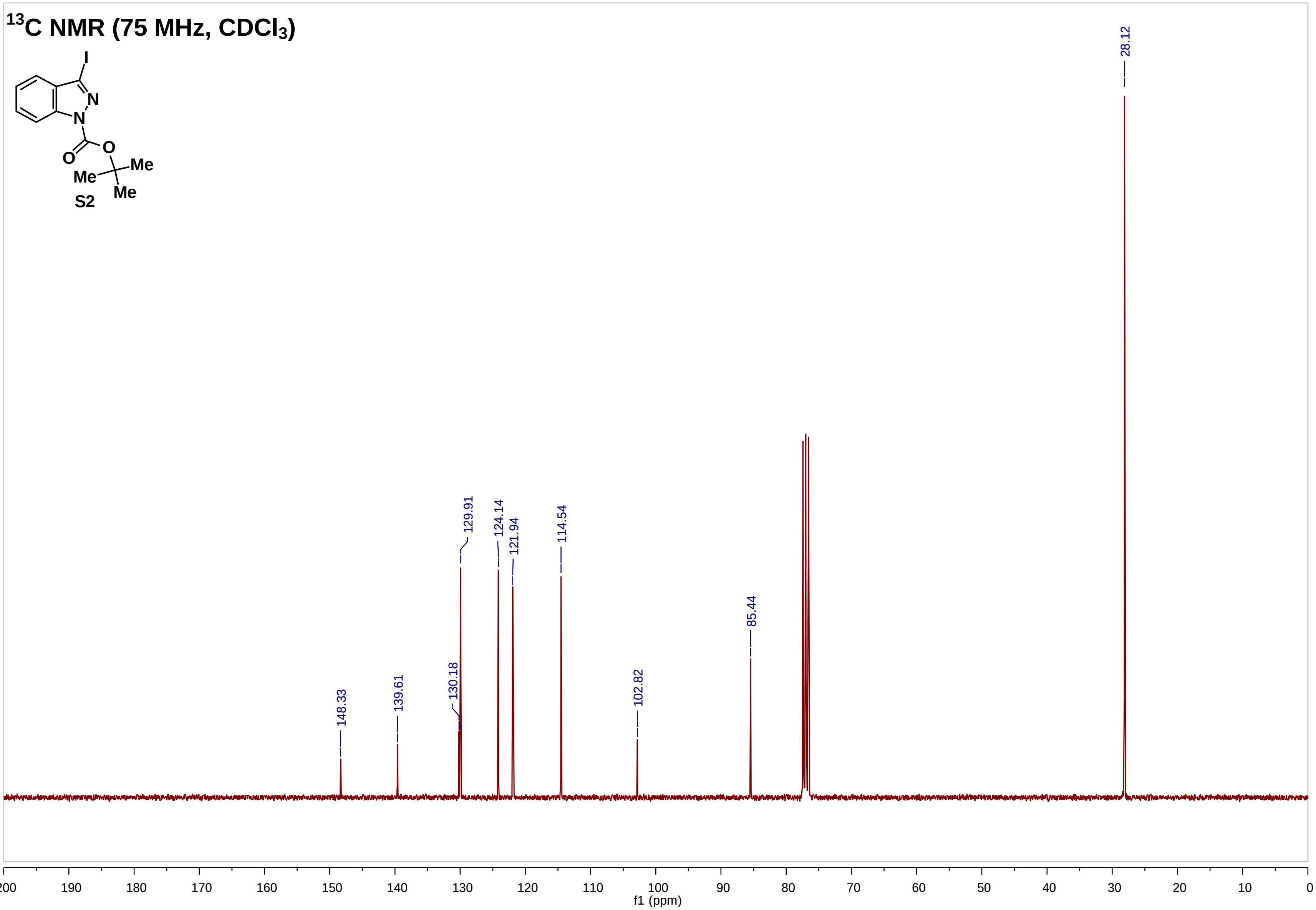

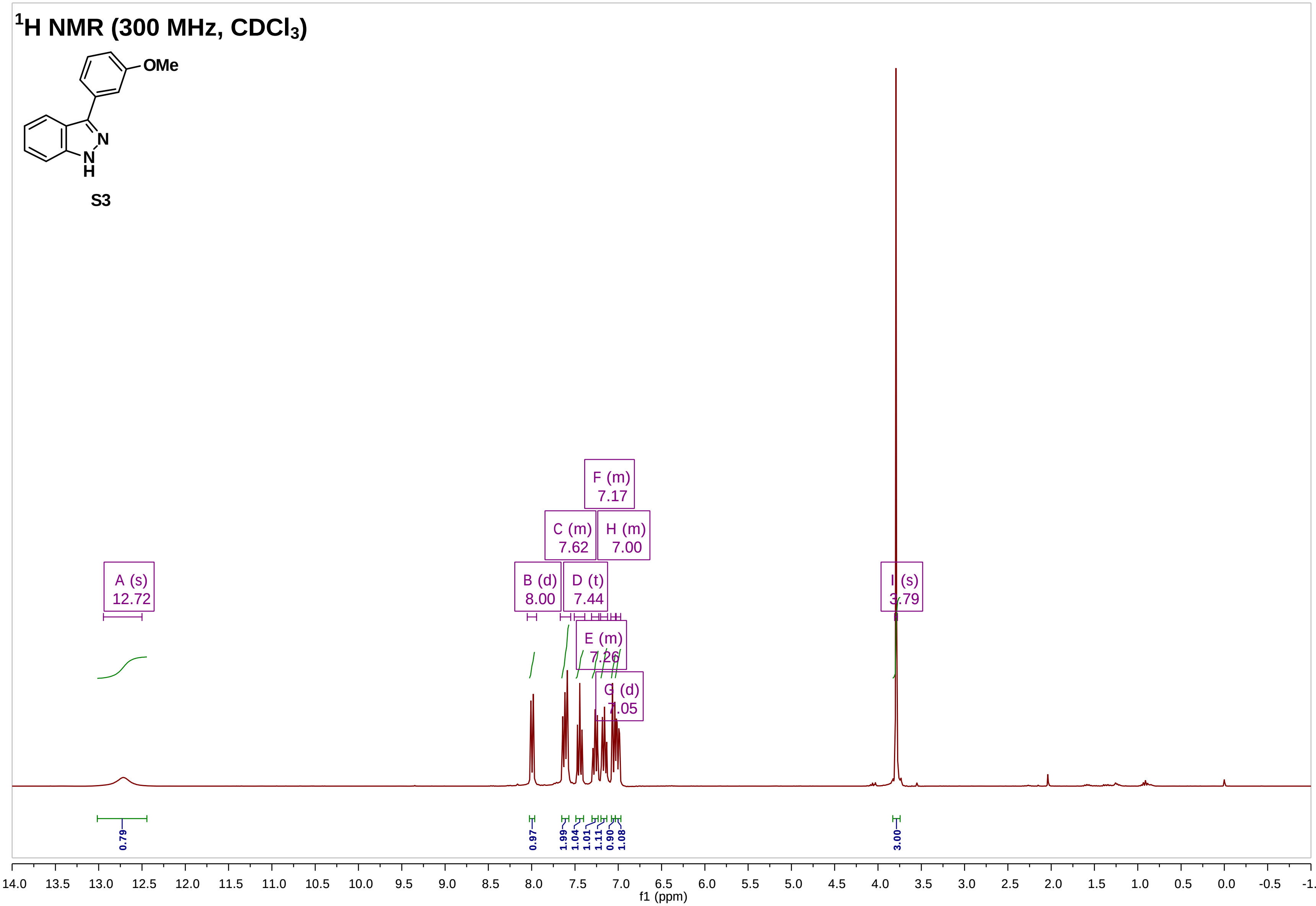

1H NMR (300 MHz, CDCl3)
OMe
N
N
H
S3
A (s)
12.72
B (d)
8.00
C (m)
7.62
D (t)
7.44
E (m)
7.26
F (m)
7.17
G (d)
7.05
H (m)
7.00
I (s)
3.79
0.79
0.97
1.99
1.04
1.01
1.11
0.90
1.08
3.00
f1 (ppm)

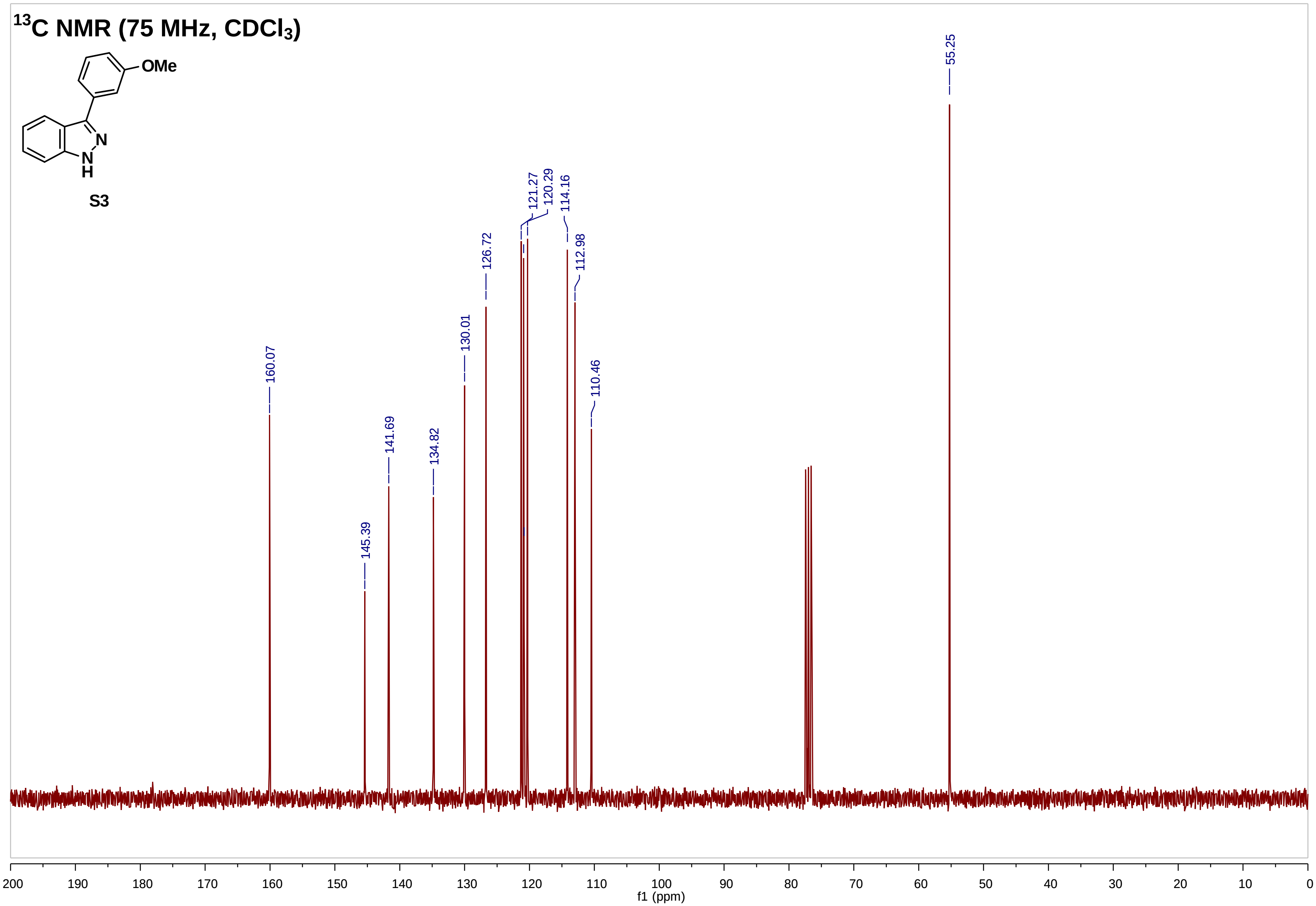

13C NMR (75 MHz, CDCl3)
OMe
N
N
H
S3
160.07
145.39
141.69
134.82
130.01
126.72
121.27
120.29
114.16
112.98
110.46
55.25
200
190
180
170
160
150
140
130
120
110
100
90
80
70
60
50
40
30
20
10
0
f1 (ppm)

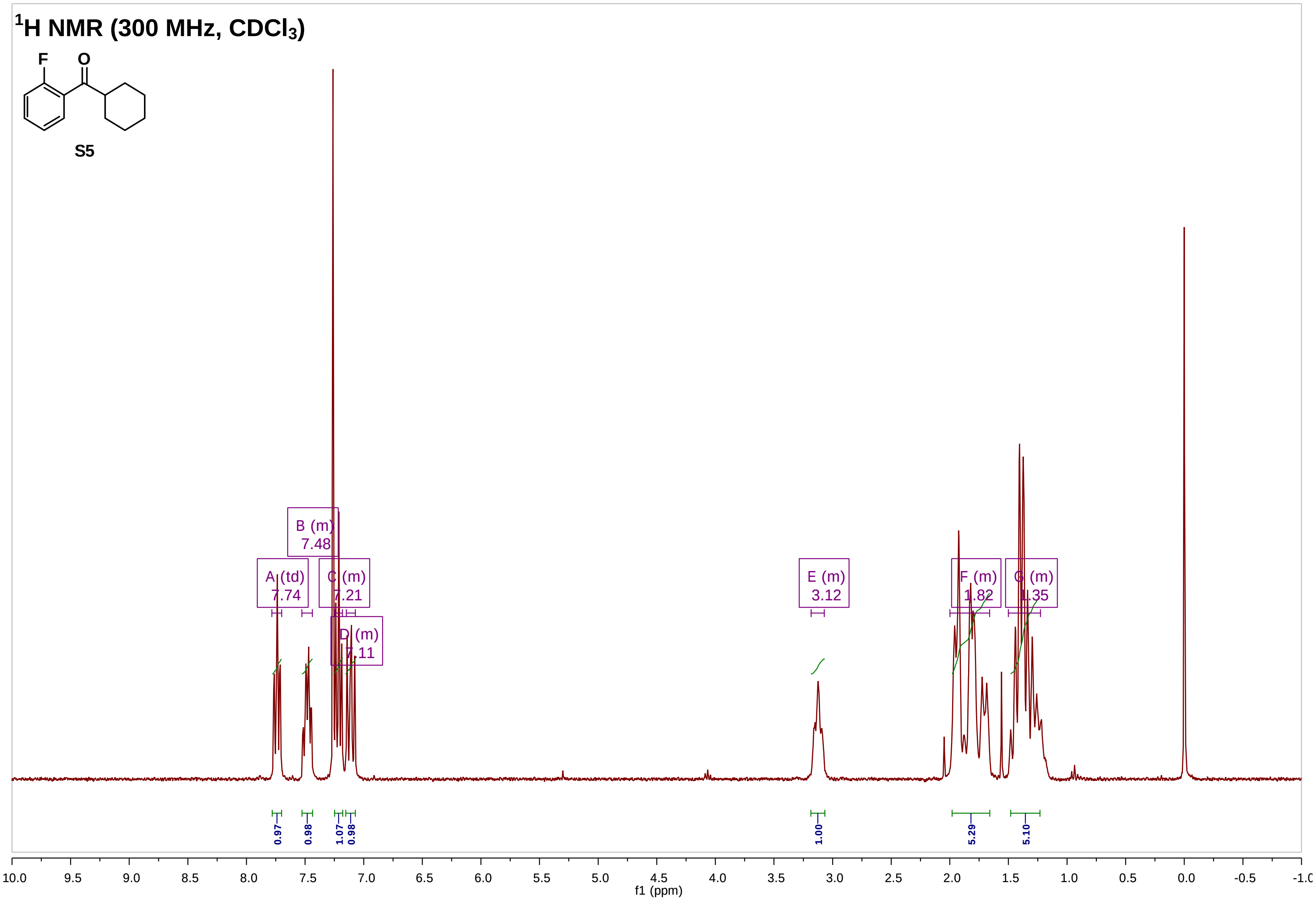

1H NMR (300 MHz, CDCl3)
F
O
S5
A (td)
7.74
B (m)
7.48
C (m)
7.21
D (m)
7.11
E (m)
3.12
F (m)
1.82
G (m)
1.35
0.97
0.98
1.07
0.98
1.00
5.29
5.10
10.0
9.5
9.0
8.5
8.0
7.5
7.0
6.5
6.0
5.5
5.0
4.5
4.0
3.5
3.0
2.5
2.0
1.5
1.0
0.5
0.0
-0.5
f1 (ppm)

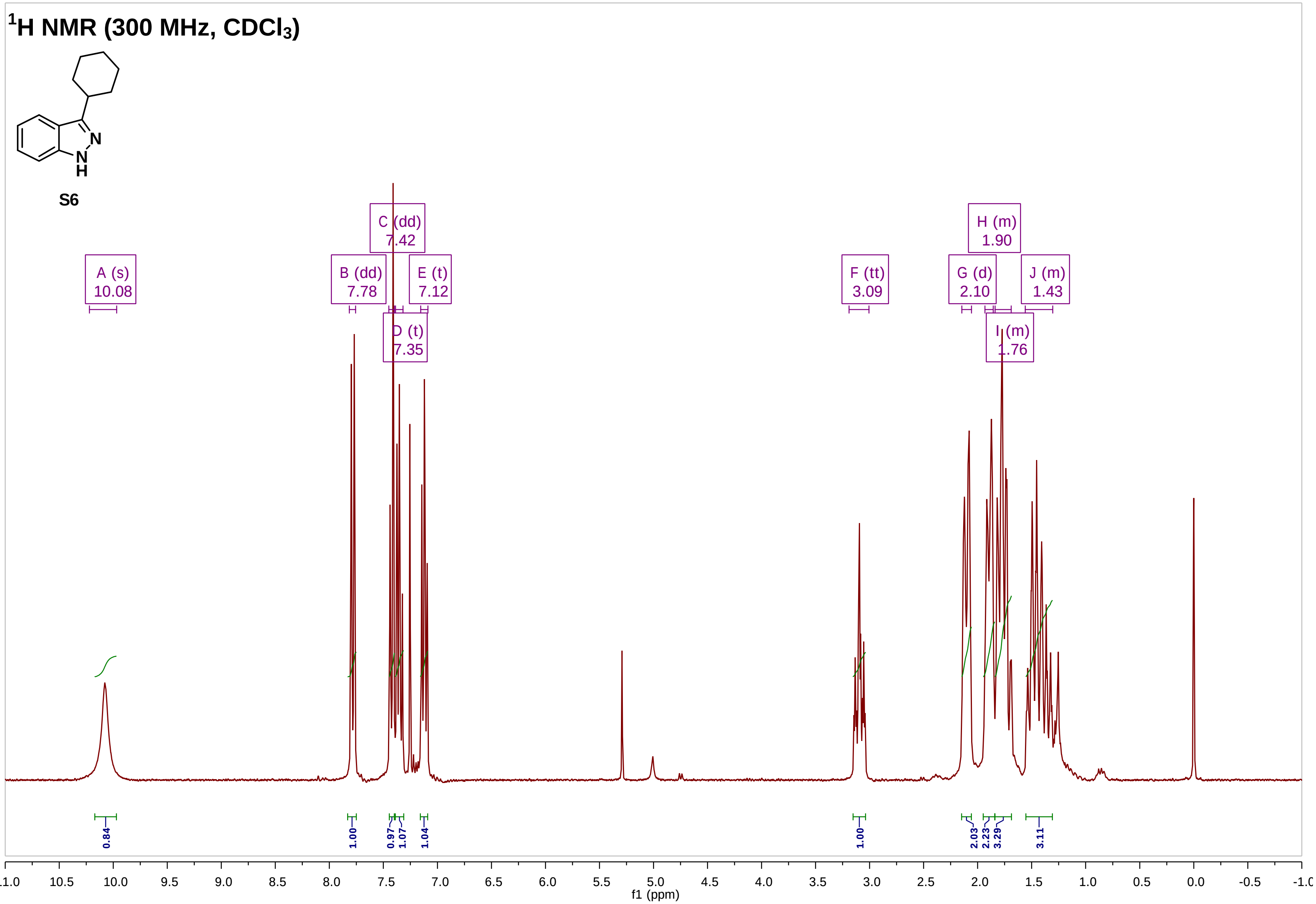

^{1}H NMR (300 MHz, CDCl$_3$)
N
N
H
S6
A (s) 10.08
B (dd) 7.78
C (dd) 7.42
D (t) 7.35
E (t) 7.12
F (tt) 3.09
G (d) 2.10
H (m) 1.90
I (m) 1.76
J (m) 1.43
0.84
1.00
0.97
1.07
1.04
1.00
2.03
2.23
3.29
3.11
11.0 10.5 10.0 9.5 9.0 8.5 8.0 7.5 7.0 6.5 6.0 5.5 5.0 4.5 4.0 3.5 3.0 2.5 2.0 1.5 1.0 0.5 0.0 -0.5
f1 (ppm)
